# Polygenic risk score prediction accuracy convergence

**DOI:** 10.1016/j.xhgg.2025.100457

**Published:** 2025-05-14

**Authors:** Léo Henches, Jihye Kim, Zhiyu Yang, Simone Rubinacci, Gabriel Pires, Clara Albiñana, Christophe Boetto, Hanna Julienne, Arthur Frouin, Antoine Auvergne, Yuka Suzuki, Sarah Djebali, Olivier Delaneau, Andrea Ganna, Bjarni Vilhjálmsson, Florian Privé, Hugues Aschard

**Affiliations:** 1 Institut Pasteur, Université de Paris, Department of Computational Biology, 75015 Paris, France; 2 Department of Epidemiology, Harvard T.H. Chan School of Public Health, Boston, MA, USA; 3 Institute for Molecular Medicine Finland (FIMM), University of Helsinki, Helsinki, Finland; 4 National Centre for Register-Based Research, Aarhus University, 8210 Aarhus, Denmark; 5 IRSD, Université de Toulouse, INSERM, INRAE, ENVT, University Toulouse III - Paul Sabatier (UPS), Toulouse, France; 6 Department of Computational Biology, University of Lausanne, Lausanne, Switzerland; 7 Bioinformatics Research Centre, Aarhus University, 8000 Aarhus, Denmark

**Keywords:** polygenic risk prediction, GWAS, sample size, polygenicity

## Abstract

Polygenic risk scores (PRSs) models trained from genome-wide association study (GWAS) results are set to play a pivotal role in biomedical research addressing multifactorial human diseases. The prospect of using these risk scores in clinical care and public health is generating both enthusiasm and controversy, with varying opinions among experts about their strengths and limitations. The performance of existing polygenic scores is still limited but is expected to improve with increasing GWAS sample sizes and the development of new, more powerful methods. Theoretically, the variance explained by PRS can be as high as the total additive genetic variance, but it is unclear how much of that variance has already been captured by PRS. Here, we conducted a retrospective analysis to assess progress in PRS prediction accuracy since the publication of the first large-scale GWASs, using data from six common human diseases with sufficient GWAS information. We show that although PRS accuracy has grown rapidly over the years, the pace of improvement from recent GWAS has decreased substantially, suggesting that merely increasing GWAS sample sizes may lead to only modest improvements in risk discrimination. We next investigated the factors influencing the maximum achievable prediction using whole-genome sequencing data from 125,000 UK Biobank participants and state-of-the-art modeling of polygenic outcomes. Our analyses suggest that increasing the variant coverage of PRS, using either more imputed variants or sequencing data, is a key component for future improvements in prediction accuracy.

## Teaser

Polygenic risk prediction has the potential to transform the diagnosis, treatment, and prevention of many common human diseases. However, the timescale and breadth of this transformation is partly unknown. This study assesses the progress made in polygenic risk prediction during the past 2 decades and identifies paths toward future improvements.

## Introduction

Most common human diseases exhibit strong polygenic inheritance, characterized by a very large number of genetic variants with small effects. This scattered distribution of risk has severely hampered the initial goal of using genetic association studies for personalized medicine through individualized disease risk predictions, prevention strategies, and treatments.^1^^,^^2^^,^^3^^,^^4^^,^^5^^,^^6^^,^^7^^,^^8^ This issue was recognized early in the genome-wide association study (GWAS) era, and the community developed a strong case for genetic risk profiling based on polygenic risk scores (PRSs) derived from GWAS results.^9^ In its simplest form, a PRS for an individual is the summation of multiple single-nucleotide polymorphisms (SNPs) weighted by their effect sizes estimated from independent GWAS data. Initially, PRSs were constructed from a small number of independent genome-wide significant variants, but they have evolved to include thousands to millions of variants selected from full GWAS results using optimized selection criteria and weighting schemes.^10^^,^^11^ As with the prediction of any highly multifactorial outcome, PRS accuracy largely depends on the sample size of the dataset used to estimate individual predictor effects. GWAS sample sizes—and therefore the predictive performance of PRSs—have increased substantially over time. However, it remains unclear how much predictive accuracy has already been achieved for common human diseases and how much further improvement can be attained through future, larger GWAS.

**Figure 1:**
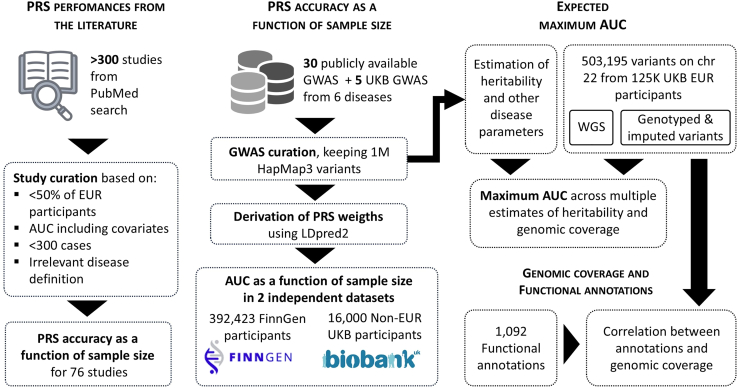
Study flowchart The primary analyses conducted in the study for the six diseases included assessing the accuracy of polygenic risk scores (PRSs) using the area under the receiver operating characteristic curve (AUC) as reported in the literature; estimating PRS performance using genome-wide association study (GWAS) summary statistics and a harmonized pipeline; evaluating the maximum AUC based on estimated disease parameters and UKB sequencing data; and analyzing the enrichment between genomic coverage and functional annotations.

Characterizing the PRS accuracy-sample size relationship in real data is challenging for two reasons. First, this relationship is expected to vary across disease parameters, including prevalence, heritability,^12^^,^^13^^,^^14^ and the distribution of effects at causal variants,^15^^,^^16^ which can be difficult to estimate in practice. Second, there is notable heterogeneity in the implementation of PRSs.^17^^,^^18^^,^^19^ Existing studies have used a variety of methods, models, data sources, and populations, including multi-ancestry^20^^,^^21^^,^^22^^,^^23^^,^^24^ and multi-phenotype approaches.^25^^,^^26^^,^^27^ This heterogeneity limits our ability to determine the key drivers of PRS improvement. A formal evaluation of PRS performance as a function of sample size requires GWASs conducted using populations of similar genetic ancestry, similar statistical tests, comparable numbers of input variants, and consistent modeling (e.g., including the same covariates). Furthermore, the PRS must be derived using the same approach and applied to a single test dataset that is not included in the GWAS used to build the PRS to avoid overfitting. This point is particularly challenging, as most existing disease GWASs are meta-analyses that include all available data. These constraints substantially limit the number of GWAS datasets that can be used. After an extensive literature review, we identified six diseases for which sufficient data meeting these criteria were available: coronary artery disease (CAD), breast cancer, type 2 diabetes, Alzheimer disease (AD), asthma, and obesity. We conducted a retrospective study to examine how PRS prediction accuracy for these six diseases has evolved with increasing GWAS sample sizes over the past 15 years. This evaluation was carried out using two independent datasets: the FinnGen cohort and non-European ancestry participants from the UK Biobank (UKB). We further investigated how existing models, state-of-the-art sequencing data, and functional annotation data may inform potential future improvements. An overview of the study design is presented in Figure 1.

## Material and methods

### GWAS data assembly

We collected publicly available GWAS summary statistics for six outcomes: type 2 diabetes, coronary heart disease, breast cancer, AD, asthma, and body mass index (BMI), for which we used to study obesity. The design of each study was carefully assessed to ensure it met stringent inclusion criteria for our analysis: (1) all studies had to include a majority of individuals of European ancestry; (2) studies with limited genetic coverage, such as exome-wide screening or genotyping chips without imputation (e.g., MetaboChip, ImmunoChip), were excluded; and (3) phenotype definitions had to be relatively homogeneous, although we retained some studies with heterogeneity for comparative purposes (e.g., early-onset asthma, AD proxy defined from parental status). In some meta-analyses, we also accessed GWAS results from individual cohorts, which were used as additional data points. After quality control filtering, 30 GWASs summary statistics with the required baseline data (coded allele, signed statistics, and *p* value) remained for analysis. We completed our panel by conducting five additional GWASs of modest sample size in the UKB using unrelated participants of European ancestry, with cases sampled from the entire cohort for four outcomes: breast cancer, CAD, asthma, and BMI. All 35 GWAS were harmonized and converted to hg38 using the *liftover* package.^28^ As one of the primary goals was to compare the predictive accuracy of PRS scores in the FinnGen cohort, we retained only variants available in that dataset. Unless otherwise specified, we defined the effective sample size of binary GWAS outcomes as $N_{eff}=4/\left[1/N_{case}+1/N_{controls}\right]$, which re-scales cohorts with unequal case-control numbers to a common unit.^29^ The list of GWAS used is presented in Table 1.

**Table 1:** GWASs used to derive polygenic risk scores

Phenotype	Reference	*N_case_*	*N_cont_*	*N*_*eff*_	No. of variants^a^
CAD	UK Biobank (5K)	5,000	50,000	18,182	1,041,959
	C4D et al.^30^	15,420	15,062	30,478	540,233
	Schunkert et al.^31^	22,233	64,762	66,204	2,420,308
	Nikpay et al.^32^	60,801	123,504	162,973	9,455,779
	Nelson et al.^33^	71,602	260,875	224,727	9,020,475
	Harst et al.^34^	34,541	261,984	122,070	7,947,838
	Harst et al.^34^	122,733	424,528	380,832	7,947,838
	Aragam et al.^23^	181,522	984,168	613,021	20,073,070
Type 2 diabetes	Cai et al.^35^	9,978	13,348	22,839	8,924,493
	Morris et al.^36^	12,171	56,862	40,101	2,473,442
	Scott et al.^37^	26,676	132,532	88,825	12,056,347
	Xue et al.^38^	62,892	596,424	227,571	5,053,016
	Mahajan et al.^39^	74,124	824,006	272,026	23,465,133
Breast cancer	UK Biobank (5K)	5,000	50,000	18,182	1,041,959
	Michailidou et al.^40^ (GWAS meta-analysis)	14,910	17,588	32,277	11,792,543
	Michailidou et al.^41^(iCOGS GWAS)	46,785	42,892	89,508	11,792,543
	Michailidou et al.^42^ (OncoArrray GWAS)	61,282	45,494	104,442	11,792,543
	Zhang et al.^43^	133,384	113,789	245,620	11,792,543
Alzheimer disease	Li et al.^44^	753	736	1,489	391,067
	Lambert et al.^45^	17,008	37,154	46,669	7,053,170
	Kunkle et al.^46^	21,982	41,944	57,693	11,480,633
	Jansen et al.^47^	71,880	383,378	242,124	13,367,300
Asthma	UK Biobank (1K)	1,000	10,000	3,636	1,041,959
	Shrine et al.^48^	5,135	25,675	17,117	33,771,859
	Moffatt et al.^49^	10,365	16,110	25,228	567,590
	Zhu et al.^50^	14,085	76,768	47,606	7,488,536
	Demenais et al.^51^	23,948	118,538	79,692	2,001,257
	Han et al.^52^	64,538	329,321	215,851	9,572,557
BMI	UK Biobank (20K)	20,000		20,000	1,041,959
	UK Biobank (80K)	80,000		80,000	1,041,959
	Speliotes et al.^53^	125,865		125,865	2,471,517
	Locke et al.^54^	322,206		322,206	2,554,638
	Yengo et al.^55^	795,640		795,640	2,336,270
	Elsworth et al.^56^	454,884		454,884	9,851,867

a Total number of variants available in the original GWAS.

### Estimation of PRS prediction performances in real data

For each GWAS, we derived a vector of variant-specific PRS weights $\gamma =\left(\gamma_{1},\gamma_{2}\ldots \gamma_{M}\right)$, where $\gamma_{i}$ is the weight for variant i and M is the number of variants available in the GWAS. We used the LDpred2 approach^11^ with the “auto” option, which automatically estimates hyperparameters, including PRS sparsity and SNP heritability. We employed 30 Gibbs sampling chains. Because the GWASs included predominantly individuals of European ancestry and in-sample linkage disequilibrium (LD) was not available, we used European descent participants from the UKB as the reference panel for LD derivation. The use of external LD data matched to the GWAS ancestry is expected to have a negligible impact on PRS accuracy.^57^^,^^58^ We restricted the analysis to GWAS variants overlapping with 1,054,330 HapMap3 variants.^59^ This restriction was due to the computational cost and memory demands of LDpred2 (also existing for other PRS software), which scale quadratically with the number of variants. For each GWAS, we also estimated per-variant effective sample sizes ($N_{eff.SNP}$) and filtered out variants with values lower than 50% or higher than 110% of the expected maximum to avoid miscalibration of regression coefficients.^57^^,^^60^ We then constructed PRSs in independent test datasets using the derived weights. The score for an individual was computed as $PRS=\gamma^{t}X$, where X is a genotype matrix with alleles coded to match the original GWAS. Because most European ancestry cohorts were used in the original GWASs, we focused our application on evaluating relative performance and trends in area under the receiver operating characteristic curve (AUC) using two datasets.

The first dataset included 16,000 unrelated UKB participants of non-European ancestry, categorized into 6 groups (Ashkenazi, Iranian, Indian, Chinese, Caribbean, and Nigerian) as defined by Privé et al.^61^ These data were also used for PRS fine-tuning and averaging weights across Gibbs sampling chains. The second dataset comprised 392,423 Finnish ancestry participants from the FinnGen cohort (see supplementary methods). Although genetic differences between training and test populations reduce absolute prediction performance, PRS portability across populations is expected to be approximately linear. Therefore, GWAS sample size vs. PRS performance trends should remain informative. For instance, the top GWAS hits between FinnGen and European samples showed high concordance (mean squared correlation of effect estimates = 0.56; Figure S1).

We evaluated PRS performance using the AUC. In the non-European UKB samples, AUCs were first computed separately for each ancestry group. Due to small case numbers per group, we meta-analyzed the results across groups using standard inverse-variance meta-analysis: ${AUC}_{combined}=\sum_{i=1\ldots 5}\left[{AUC}_{i}/\sigma_{i}^{2}\right]/\sum_{i=1\ldots 5}\left[1/\sigma_{i}^{2}\right]$, and $SE\left({AUC}_{combined}\right)=1/\sum_{i=1\ldots 5}\left[1/\sigma_{i}^{2}\right]$, where ${AUC}_{i}$ and $\sigma_{i}^{2}$ represent the AUC and variance for population i=1…5. Importantly, no additional conventional risk factors (e.g., clinical, demographic, environmental) were included in the models, so the reported AUCs reflect the marginal predictive power of polygenic models alone.

### Variance captured by genotyped and imputed variants

We examined the variance captured by genotyped and imputed variants using real genetic data and simulated genetic effects. Consider a standardized phenotype Y drawn from a polygenic model and defined as the linear additive effect of M standardized causal variants. Its total variance equals $V_{y}=h^{2}+V_{e}$, where $h^{2}$ is the genetic variance (heritability) and $V_{e}$ is the residual environmental variance. Assuming that genetic effects at causal variants are independent of variant correlations, heritability can be approximated as $h^{2}=\sum_{M}\beta_{i}^{2}$, where $\beta_{i}$ is the effect of a standardized variant i. We estimated the proportion of $h^{2}$ that could be recovered under an infinite GWAS sample size when only a subset S of variants has been genotyped, while the remaining M∉Ss are either imputed or missing. Two key metrics were considered: $h_{I}^{2}$, the additive genetic variance captured by all M variants, both genotyped and imputed using advanced methods,^62^ and $h_{G}^{2}$, the genetic variance captured using only the subset S of genotyped variants but accounting for the contribution of the M∉S untyped variants through LD. The first metric is defined as $h_{I}^{2}=\sum_{M}\beta_{i}^{2}r_{imput}^{2}$, where $r_{imput}^{2}$ is the squared correlation between the sequenced variant i and its imputed value. The second metric is defined as $h_{G}^{2}=\sum_{S}\beta_{i}^{2}+\sum_{M\notin S}\beta_{j}^{2}\rho_{j}^{2}$, where $\rho_{j}^{2}$ represents the squared correlation between the untyped variants j and the set of genotyped variants. In theory, $h_{G}^{2}$ should closely approximate GWAS-based heritability ($h_{GWAS}^{2}$) as estimated by existing software.^63^ The former ($h_{I}^{2}$) is expected to capture additional variance from untyped variants poorly tagged by the genotyped ones.

Both metrics were estimated using a subset of 125,152 UKB participants of European ancestry with both genome-wide genotyping and whole-genome sequencing data. For $h_{I}^{2}$, we used imputed SNP-array data from the UKB. Imputed variants were lifted over to GRCh38, retaining 99.5% of sites. We then computed $r_{imput}^{2}$ between each sequenced variant and its imputed dosage using standard univariate linear regression. For $h_{G}^{2}$, we used genotyped variants available on the UKB Axiom array^64^ as the baseline. The $\rho^{2}$ values were computed using adjusted squared correlation obtained from standard multiple linear regressions, where each non-genotyped variant was predicted from a set Ω of nearby genotyped variants within a ±1.5-Mb window. For simplicity, both $r_{imput}^{2}$ and $\rho^{2}$ were calculated using only chromosome 22, assuming that it is representative of the entire genome. This chromosome includes 12,968 genotyped variants. Of the 659,092 sequenced variants, 503,195 remained after filtering out those with minor allele frequency (MAF) < 0.001%. As expected, the two metrics were highly correlated ($cor\left(\rho^{2},r_{imput}^{2}\right)=0.71$), but the average $r_{imput}^{2}$ was substantially higher than $\rho^{2}$ ($\bar{r_{imput}^{2}}=0.50$, $\bar{\rho^{2}}=0.25$).

In our simulations, the proportion of heritability captured was derived using both $r_{imput}^{2}$ and $\rho^{2}$ and effect size $\beta =\left(\beta_{1}\ldots \beta_{M}\right)$ drawn from a normal distribution under the alpha model, where the expected effect of a variant is proportional to its variance raised to a power α.^65^^,^^66^^,^^67^ This model posits that rare variants have larger per-allele effects than common variants when α < 0. The genetic effect for variant i was drawn from $\beta_{i}|p_{i}∼N\left(0,\sigma_{g,\alpha }^{2}\cdot {\left[2p_{i}\left(1-p_{i}\right)\right]}^{\alpha }\right)$, where $p_{i}$ is the MAF and $\sigma_{g,\alpha }^{2}$ is scaled to match a predefined heritability. In our analysis, we considered α values ranging from −1.5 to 0 and sampled $\sigma_{g,\alpha }^{2}$ uniformly over [0, 1]. Additionally, we explored an attenuated alpha model that reduces the influence of rare variants using an ad hoc iterative weighting function (see supplementary methods).

### Maximum achievable AUC

The expected maximum achievable AUC from a polygenic model is determined primarily by the proportion of genetic variance captured by the variants used to construct the PRS. This maximum can be derived using the approximation proposed by Wray et al.^14^: ${AUC}_{\max}\approx \Phi \left(\left(\left(i-v\right)h^{2}\right)/\sqrt{h^{2}\left[\left(1-h^{2}i\left(i-T\right)\right)+\left(1-h^{2}v\left(v-T\right)\right)\right]}\right)$, where $h^{2}$ is the heritability on the liability scale, Φ is the cumulative density function of the normal distribution, z is the height of the standard normal density at the threshold $T=\Phi^{-1}\left(1-K\right)$ and with i=z/K and v=−z/(1−K), where K is the disease prevalence. We confirmed the validity of this approximation through simulation models involving independent causal variants with linear additive effect $h^{2}$ values in [0.2, 0.7] and prevalence K in [0.01, 0.25] (supplementary methods). Conditional estimates of AUC were derived by substituting $h^{2}$ with either $h_{G}^{2}$ or $h_{I}^{2}$, themselves derived based on alpha drawn in [−1.5, 0] and $\beta =\left(\beta_{1}\ldots \beta_{M}\right)$ coefficients sampled from a normal distribution. For real-data analysis, disease prevalence values were sourced from the Centers for Disease Control and Prevention (CDC) website. We derived three estimates of heritability on the liability scale: (1) the total heritability derived from twins studies for CAD (0.55),^68^ type 2 diabetes (0.72),^69^ breast cancer (0.27),^70^ AD after excluding the effect of *APOE* (0.49),^71^^,^^72^ asthma (0.70),^73^ and BMI (0.75)^74^; (2) $h_{GWAS}^{2}$, the heritability captured by GWAS variants and derived using five alternative approaches: SBayesS,^75^ sumHer,^76^ LD score regression (LDSC) regression,^77^ GENESIS,^15^ and MiXeR^78^ (supplementary methods); and (3) $h_{I}^{2}$, the heritability captured by genotyped and imputed variants, which requires an estimate of the total heritability, the proportion of heritability captured by genotyped and imputed variants given α, and a value of α. For the total heritability, we used the twins studies estimates. For the proportion of heritability captured, we used $r_{imput}^{2}$, the estimate derived using the UKB sequencing data. The choice of α was more challenging, and we ultimately used an *ad hoc* approach. Further details are provided below.

Most real-data estimates of $h_{GWAS}^{2}$ are derived from genotyped variants and a modest subset of imputed variants filtered for high quality (typical info score ≥0.8). As a result, assuming our estimation of the heritability captured by genotyped variants is valid, the previously described $h_{G}^{2}$ is expected to approximate $h_{GWAS}^{2}$ if provided a relevant value of α. We assessed this equality using α derived from various approaches (SBayesS, sumHer, and individual-level data from the UKB), but we found large discrepancies. Ultimately, we adopted an *ad hoc* “best-fit” α approach: for each disease, we selected α so that $h_{G}^{2}$ equals the median of the five GWAS-based heritability estimates. This α was then used to derive $h_{I}^{2}$ and compute AUC predictions accordingly.

### Genetic coverage and functional annotations

We evaluated the association between a range of functional annotations and the imputation quality ($r_{imput}^{2}$, the squared correlation between sequenced and imputed genotypes in the UKB). We used a total of 1,099 functional annotations pulled from mutiple sources: baseline GENCODE annotations, including intron, gene, exon, coding DNA sequence (CDS), transcription start site (TSS), transcription termination site (TTS), and untranslated regions (UTRs); epigenetic features across tissues and cell types: transcription factor binding sites (TFBSs) and functional annotation of the mammalian genome version 5 (FANTOM5); and regulatory elements: promoters, enhancers, dyadic annotations from Roadmap, DNase I hypersensitive sites (DHSs) from two sources, and super-enhancers. The analysis was performed on the same dataset used to derive $h_{I}^{2}$ and $h_{G}^{2}$ (503,195 sequenced chromosome 22 variants from 125,000 UKB participants of European ancestry). Associations between each functional annotation $A_{i}$ and imputation quality were estimated using a standard univariate linear model: $r_{imput}^{2}\;∼\delta_{i}\;A_{i}$. A sensitivity analysis was also conducted using a model adjusted for GENCODE annotations, $r_{imput}^{2}\;∼\delta_{i}\;A_{i}+\sum_{k\in GENCODE}\delta_{k}\;A_{k}$, where $A_{k}$ denotes GENCODE category indicators.

## Results

### PRS prediction accuracy and sample size

**Figure 2:**
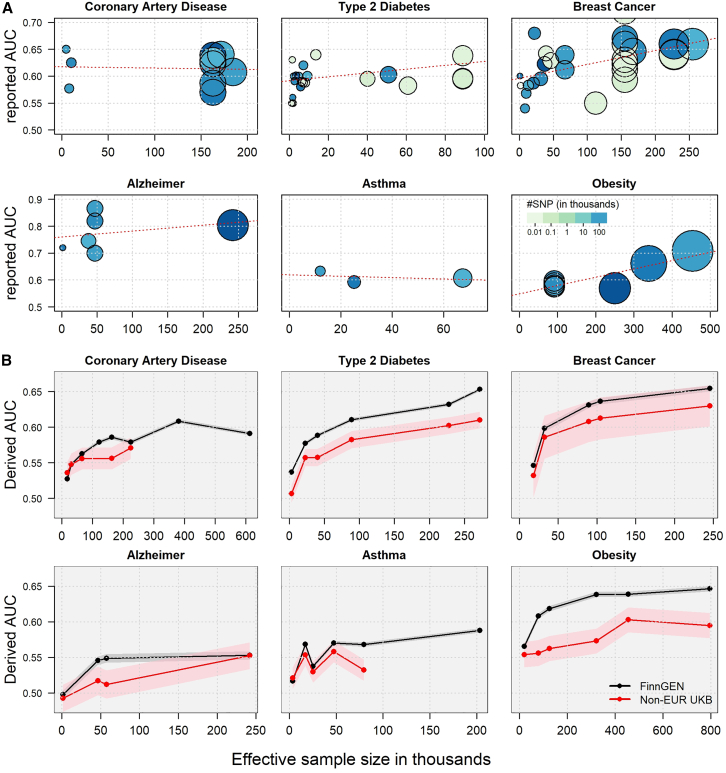
PRSs predictive accuracy as a function of sample size (A) AUC reported in the literature for PRS as a function of the effective sample size across six diseases: coronary artery disease, type 2 diabetes, breast cancer, Alzheimer disease (AD), asthma, and obesity. The color gradient represents the number of variants used, ranging from a few top-associated variants (light green) to millions (dark blue), and the size is proportional to the logarithm of the effective sample size. (B) AUCs for the 6 outcomes derived using a harmonized pipeline. PRSs were trained from 35 GWASs using the LDpred2 approach and tested with individual-level data from the FinnGen cohort (black) and in 6 non-European ancestry UKB populations (red, meta-analysis over 6 populations). The AUCs are plotted against the effective sample size of the corresponding GWAS. Missing values indicate instances where there was a sample overlap between the test and train sets. The 95% confidence intervals are shown as shaded red and gray for the UKB and FinnGen analyses, respectively.

To illustrate the challenge of characterizing the sample size-PRS accuracy relationship in real data, we reviewed the literature and curated previous reports of genetic risk score prediction accuracy, expressed as the AUC, for CAD, breast cancer, type 2 diabetes, AD, asthma, and obesity (Table S1; supplementary methods). The studies spanned from 2006 to 2023 and the effective sample size ($N_{eff}$) ranged from 981 to 453,912. The reported predictive power showed a very modest linear trend with sample size and was instead characterized by substantial heterogeneity (Figure 2A). AUC increases were nominally significant for breast cancer (β = 2.5 × 10^−4^/1,000 increase in sample size, *p* = 0.0096) and obesity (β = 3.1 × 10^−4^, *p* = 0.0038), but were not significant for the other outcomes. For example, there was no clear trend for CAD (β = −1.8 × 10^−5^, *p* = 0.90) despite an effective sample size ranging from $N_{eff}$ = 4,522 to $N_{eff}$ = 184,305. This is likely due to several factors already discussed in the literature and complex to disentangle, including heterogeneity in the population characteristics (age, sex, fine-scale genetic ancestry within European population), disease definition, and the method used to derive the PRS weights.^17^^,^^19^

Prediction accuracy was then assessed using a harmonized pipeline, in which PRSs were derived from 35 curated GWASs and applied to two independent cohorts: FinnGen and non-European UKB participants. It shows a clear, non-linear increasing trend as a function of sample size, starting with a sharp rise for the first few studies, followed by a gradual decline in improvement (Figures 2B and S2; Tables S2–S4). The patterns are highly concordant across the two test datasets, displaying only the expected offset due to genetic ancestry differences between the training and test data.^61^^,^^79^ The flattening of AUC improvement is especially striking for type 2 diabetes, obesity, breast cancer, and CAD. Asthma displays a noisier trend, potentially reflecting challenges in disease definition and diagnosis. AD shows a continuous increase in AUC for the non-European UKB samples, but a negligible increase for the most recent and largest GWAS in FinnGen, despite a 5-fold increase in effective sample size. This may be explained by the specificity of the AD PRS (Figure S3) and, in particular, the use of a proxy for disease status—a score based on the AD status of participants’ parents—in this GWAS, which could introduce variability in heritability estimates of AD^80^ and likely affect disease risk prediction.

Sensitivity analyses yield comparable results. Using alternative approaches to derive the PRS from the same curated GWAS data produces qualitatively similar prediction accuracy, but with higher heterogeneity (Figure S4; Table S5). Trends in prediction accuracy, measured by the coefficient of determination (*R*^*2*^) and the odds ratios of the disease comparing the top 5% and top 1% PRS strata to the remaining population, are fully consistent with those observed for the AUC (Figure S5; Table S5), with additional variability only in the top 1% PRS, likely due to the modest sample size when using this stringent threshold. We also confirm the relevance of our stringent GWAS curation, in which we removed all GWASs displaying potential overlap with our testing samples. As shown in the experiment from Figure S6, even a small overlap between the training and test samples can artificially inflate prediction accuracy.

### Modeling and robustness of AUC convergence

The convergence of prediction accuracy toward its maximum can be demonstrated using simple theoretical models^14^ (Figure S7). Predicting the convergence rate in real data is considerably more challenging and requires estimating multiple parameters of the disease genetic model, including heritability, polygenicity, the distribution of causal genetic effects, and the dependence of those effects on LD, functional annotations, and MAF. We assessed the ability of GENESIS,^15^ an approach that utilizes some of the aforementioned disease parameters, to predict the observed AUC trend using each of the 35 GWASs summary statistics. As shown in Figure S8A, the predictions diverge substantially from the trend observed in FinnGen. This is likely partly due to uncertainty in the estimated disease parameters (Figures S8B and S8C). Estimates of these parameters, obtained from alternative tools,^15^^,^^75^^,^^76^^,^^77^^,^^81^ show similar variability depending on the input GWAS, with confidence intervals often not overlapping across GWASs for the same outcome (Figures S8 and S9; Table S6). The reasons for this variability are unclear, but until progress is made in deriving these estimates, our ability to model the convergence rate of PRS predictive power will likely remain limited, highlighting the importance of conducting retrospective studies.

We next investigated the extent to which the observed decrease in AUC improvement with sample size might be confounded by increasing heterogeneity (e.g., variability in disease definition, clinical and environmental characteristics of the participants) arising from meta-analyzing an increasing number of studies. We first compared the observed AUC trend with the trend derived using GWAS training and test data sampled exclusively from UKB participants of European ancestry, thereby reducing potential heterogeneity. We focused on obesity, one of the few scenarios that allowed for achieving a reasonably large sample size within a single homogeneous cohort. Overall, the AUC from those experiments followed the same trend as those derived in FinnGen (Figure S10) and did not suggest any effect of heterogeneity on the AUC trend in these data. We also assessed the performance of PRSs derived from the CAD GWAS in predicting seven intermediate CAD phenotypes in FinnGen. Although the absolute accuracy increased almost linearly with stricter outcome definitions in the test set, the trends remained highly consistent across all intermediate phenotypes (Figure S11).

Further direct assessment of the impact of heterogeneity is challenging, as it would require repeating the analysis from Figure 2B across homogeneous population strata and refined disease definitions. Such data are typically not available; however, some complementary indirect evaluations are possible. Heterogeneity in GWAS design and population is expected to produce heterogeneity in variant-outcome associations, such that increasing heterogeneity with sample size should lead to reduced heritability and increased polygenicity. As shown in Figures S8 and S9, we did not observe any clear evidence of such effects across the GWAS used, nor any marked difference in the trends of genetic parameter estimates when compared to the aforementioned homogeneous UKB experiment (Figures S10A–S10C).

### Maximal achievable prediction

**Figure 3:**
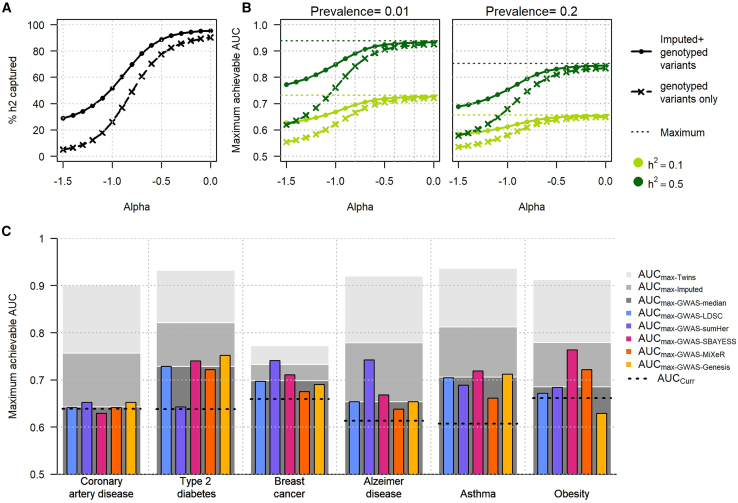
Heritability captured and maximum achievable AUC (A) The expected proportion of heritability captured by genotyped variants only (dashed line) and by both genotyped and all imputed variants, regardless of their imputation quality (solid line), conditional on the minor allele frequency (MAF)-effect size relationship (alpha). Individual-level data from the UKB were used to estimate the squared correlation between sequenced variants and either genotyped variants ($\rho^{2}$) or imputed variants ($r_{imput}^{2}$). Genetic effects were assumed to be distributed following an alpha model. (B) The corresponding maximum achievable AUC for heritability of 0.1 and 0.5 and disease prevalences of 0.01 and 0.2. (C) Estimates of the maximum achievable AUC for six outcomes: coronary artery disease, type 2 diabetes, breast cancer, AD, asthma, and obesity (using body mass index GWAS). These AUCs were derived based on US disease prevalence and various heritability estimates: twins studies (AUC_Twin_), heritability derived using five competitive approaches (LDSC regression, sumHer, SBayesS, GENESIS, and MiXer, labeled AUC_GWAS_) applied to the largest GWAS available for each disease, and twins study heritability captured by imputed variants (AUC_Imputed_). The black dashed lines indicate the most recent AUC estimates from real data in the literature, derived from approximately 1 million HapMap3 variants. For AD, heritability estimates and current AUC estimates exclude the *APOE* region.

The maximum achievable AUC from a polygenic model relies primarily on variant coverage—that is, the proportion of genetic variability—and concurrently, the proportion of heritability, captured by the variants used to derive the PRS. Existing PRSs are built entirely from genome-wide genotyping arrays complemented by imputation, which offer cost-effective coverage of common genetic variants^82^ in very large cohorts. This implies that even with very large GWAS sample sizes, maximum prediction accuracy is still bounded by this sub-sampling of existing variants, as compared to using whole-genome sequencing data.^65^^,^^83^^,^^84^^,^^85^^,^^86^^,^^87^ As shown in the theoretical models from Figure 3A, the proportion of total heritability captured by genotyped variants ($h_{G}^{2}$) can vary substantially depending on the relationship between effect sizes and MAFs, as parametrized by the so-called alpha model^65^^,^^66^ (Figures S12–S14). Using all imputed variants, including those with modest or poor imputation quality—typically filtered out in GWAS studies—can recover a substantial share of total heritability. For example, assuming a random distribution of genetic effects across the genome, $h_{I}^{2}$ varies from 29% for α=−1.5 to 96% for α=0. In comparison, $h_{G}^{2}$ varies from 5% for α=−1.5 to 90% for α=0. Notably, previous studies have argued that the alpha model might overestimate the effect of rare variants.^66^ To address this potential limitation, we devised an attenuated alpha model that implies a reduced contribution of rare variants (Figures S12B–S12D; supplementary methods). However, when comparing these attenuated models to the baseline alpha model using real data, we found no evidence for improved fit (Figures S15 and S16).

The proportion of heritability captured can be translated into maximum achievable AUC. As expected, this maximum increases with higher heritability and lower disease prevalence^14^ (Figure 3B). We compared the expected maximum AUC in real data for each of the six diseases using three heritability estimates: twins study heritability (AUC_max-twin_), GWAS-based heritability derived from five approaches (AUC_max-GWAS_; Table S7), and heritability captured by all imputed variants, regardless of their imputation quality (AUC_max-Imputed_). Figure 3C presents all estimates in a single panel. First, the AUC_max-GWAS_ estimates are relatively close to the AUCs reported in recent studies (AUC_curr_; Table S7) for CAD, breast cancer, AD, and obesity, confirming that increasing GWAS sample size for these outcomes is unlikely to dramatically improve prediction performance. Conversely, AUC_curr_ for T2D and asthma display sizable gaps compared to AUC_max-GWAS_, suggesting that despite the trend observed in Figure 2B, future GWASs with larger sample sizes may still provide a slow but continuous improvement in prediction. Second, the gap between AUC_max-twin_ and AUC_max-GWAS_ is large for all outcomes except for breast cancer, suggesting that increasing variant coverage in future studies could dramatically improve the predictive power of PRSs for the former outcomes. Third, AUC_max-Imputed_ is substantially higher than AUC_max-GWAS_ for all outcomes except breast cancer. Hence, future PRSs using an increasing number of imputed variants, even those with poor imputation quality, have the potential to boost predictive power without requiring costly sequencing data. As a sensitivity analysis, we also re-estimated all maximum achievable AUC values using prevalence data from other sources and found no qualitative impact on the results (Figure S17).

### Effect distributions and predicted performances

In previous analyses, we assumed that causal variants were randomly distributed across the genome and modeled only the relationship between effect sizes and MAFs. However, there is strong evidence that causal variants are highly enriched in certain functionally annotated regions.^88^^,^^89^^,^^90^^,^^91^ Modeling this conditional distribution may affect both the convergence of the AUC and the expected maximum achievable AUC. Nonetheless, the precise relationship between functional annotations and causal variants is not yet fully understood and likely depends on the specific outcome studied. Instead, we estimated the association between the quality of imputation and 1,099 annotations (Figure S18; Tables S8 and S9), including gene elements, DHSs, enhancers, and promoters. These annotations cover 0.001%–64.7% of the whole genome (Table S9).

**Figure 4:**
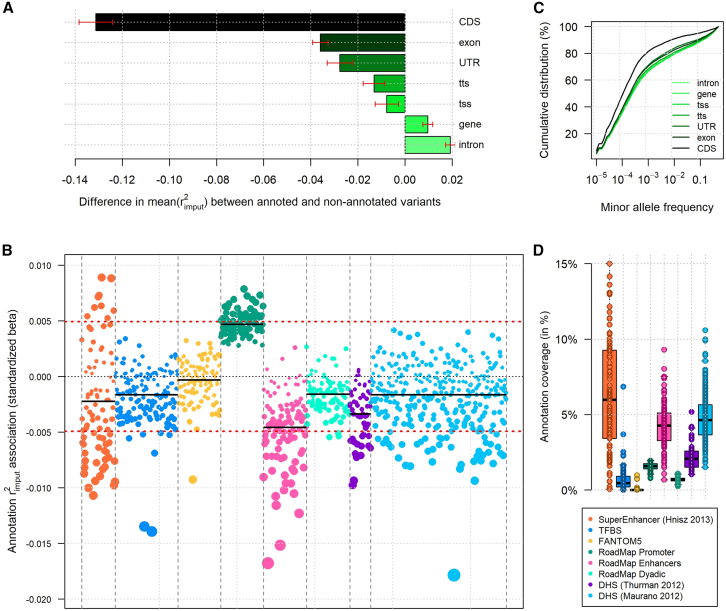
Imputation quality varies across functional annotations (A–D) We computed the relationship between functional annotations and imputation quality, measured as the squared correlation ($r_{imput}^{2}$) between true and imputed genotypes. (A) The difference in the average $r_{imput}^{2}$ for chromosome 22 across GENCODE annotations: introns, genes, exons, CDS (coding DNA sequence), tss (transcription start site), tts (transcription termination site), and UTR (untranslated region). Red bars indicate ±2 SDs from the mean, encompassing 95% of the annotated regions. (B) Standardized regression coefficients from the univariate association between the measured $r_{imput}^{2}$ and each of the 1,092 functional annotations across 8 categories: TFBS (transcription factor binding site), FANTOM5 (functional annotation of the mammalian genome version 5) regulatory regions, promoters, enhancers, and dyadic regions from Roadmap, DHS (DNase I hypersensitive sites) derived from two studies, and super-enhancers. Horizontal black lines represent the average per category, and the red dashed lines indicate the significance threshold after correction for multiple testing. (C) The cumulative distribution of variants for each GENCODE category as a function of the MAF. (D) The distribution of annotation frequencies, grouped by category.

The strongest associations with imputation quality were observed for gene elements (Figures 4A and S19A). CDSs were significantly negatively associated with $r_{imput}^{2}$ (*p* = 1.8 × 10^−307^), with average $r_{imput}^{2}$ values of 0.27 and 0.40 for CDSs and non-CDS variants, respectively. Exonic variants had lower imputation quality (*p* = 2.0 × 10^−131^), with average $r_{imput}^{2}$ values of 0.37 and 0.40 for exonic and non-exonic variants, respectively. Other annotation categories exhibited weaker but statistically significant negative and positive associations (Figure 4B). Enhancers, super-enhancers, DHSs, and TFBSs generally showed reduced imputation quality compared to the average of the genome, while variants within promoters tended to have slightly higher imputation accuracy. No clear enrichment was observed for specific cell types or tissues among other top significant annotations (Figure S20). The observed reduction in imputation quality within certain annotations may partly reflect an enrichment for rare variants. For example, coding regions displayed a significantly higher proportion of rare variants (*p* = 2.5 × 10^−270^; Figure 4C; Table S10). As discussed in prior research, causal variants may have lower LD with neighboring variants because of selective pressure,^86^^,^^90^ an assumption that is now commonly incorporated in disease parameters estimation tools.^92^ Lower LD leads to lower $r_{imput}^{2}$, meaning the observed lower coverage in CDSs, enhancers, DHSs, and TFBSs may indicate an enrichment for rare, poorly tagged causal variants in these regions. In such scenarios, our estimates of the maximum achievable AUC from imputed variants (Figure 3) could be slightly overestimated. These findings further suggest that improving variant coverage, especially in underrepresented functional regions, will be crucial for advancing PRS predictive accuracy in future studies (Figure S20).

## Discussion

Polygenic risk prediction holds significant potential to transform the diagnosis, treatment, and prevention of many common human diseases. However, the timescale and magnitude of this transformation remain uncertain, and concerns persist within the scientific community regarding the practical relevance of the PRS. These concerns are partly due to inconsistencies in reported performances metrics. Many existing studies lack complete documentation, and while guidelines have been proposed,^17^^,^^18^^,^^19^ adherence to these guidelines remains limited. Heterogeneity in methodology, study populations, and included covariates often impedes replication and complicates formal comparisons of PRS performance. As demonstrated in this study using six outcomes, even rigorous curation of published results may not be sufficient to clarify the relationship between prediction performance and study parameters.

Here, we demonstrate that the relationship between PRS prediction accuracy and GWAS sample size is unequivocal and highly replicable when using harmonized data preprocessing and analysis pipelines. Our results show that the accuracy of PRSs derived from existing GWASs has begun to plateau for most studied diseases. As a consequence, expanding current efforts (i.e., solely increasing GWAS sample sizes) may result in only modest improvements in predictive power. For some outcomes, including CAD, breast cancer, and obesity, GWAS-based PRS performance appears to be nearing the expected maximum based on GWAS-derived heritability. For type 2 diabetes and asthma, a larger gap remains between current and theoretical maximum AUC values, suggesting that further gains are still achievable, albeit requiring very large GWAS sample sizes. Our analyses also suggest that without large-scale whole-genome sequencing data, the ceiling for prediction accuracy in GWAS-based PRS will remain constrained by the variant coverage of GWAS array. Importantly, this study supports previous findings^85^ advocating for the inclusion of both rare and common imputed variants with modest imputation quality. These can serve as an effective intermediate strategy to enhance PRS performance, although this will require substantial methodological development.

Several other factors could further improve PRS predictive power and raise the ceiling on maximum achievable AUC. First, the observed plateau in AUC may partly reflect growing heterogeneity in large meta-analyses (e.g., looser disease definitions or more genetically diverse populations), undertaken to allow for a broader inclusion of participants. Although our results did not show strong evidence of such an effect across the studied outcomes, the influence of heterogeneity in genetic ancestry^61^^,^^79^ and phenotype definition^80^^,^^93^ is well documented. Second, we assumed homogeneity of genetic effects across individuals. Future PRSs may benefit from accounting for potential effect heterogeneity linked to demographic characteristics, basic health parameters, and lifestyle.^7^^,^^94^^,^^95^ For instance, heritability has been shown to vary with age and sex.^96^^,^^97^ Third, both the PRSs and their maximum expected AUCs depend on estimates of genetic architecture parameters. As illustrated in the present study, estimates of heritability, polygenicity, and the effect size-MAF relationship (alpha) vary substantially across methods and input GWASs. Resolving these inconsistencies will be key to improving both PRS construction and the modeling of their predictive ceilings. Additionally, we had to perform extensive preprocessing of GWASs summary statistics. Improved quality control and variants filtering^60^^,^^98^ may also yield more reliable parameter estimates.

This study has several limitations in its design. First, we assumed that causal variants are randomly distributed across the genome and only modeled the relationship between MAF and effect size. This assumption is likely an oversimplification of the true underlying model, as prior research has shown enrichment of causal variants in specific LD patterns and functional annotations.^15^^,^^16^^,^^65^^,^^78^^,^^99^^,^^100^ Rather than investigating specific models, we examined the relationship between variant coverage and functional annotations, observing both positive and negative enrichment across features. However, except for CDSs that show substantially poorer coverage, these differences did not qualitatively affect our conclusions. Second, we focused on PRSs derived from standard univariate GWASs and did not consider more recent, potentially more powerful methods, such as multi-ancestry^20^^,^^21^^,^^22^^,^^23^^,^^24^ or multi-phenotype approaches.^25^^,^^26^^,^^27^ These methods may increase power by improving effect size estimation, potentially mimicking an effective sample size increase. However, they are not likely to alter the fundamental convergence pattern toward the theoretical maximum AUC. Third, our models included PRSs only and did not incorporate additional non-genetic risk factors. Thus, the reported AUCs reflect only the marginal predictive power of polygenic risk models. In practice, the incremental value of a PRS depends on how much predictive power is already captured by conventional clinical risk factors and their correlation with the PRS.^101^^,^^102^ Fourth, for real-data heritability estimates, we relied on twins studies as proxies for total additive genetic variance. These estimates may be biased due to shared environmental effects and non-additive interactions, such as gene-gene and gene-environment effects.^103^ Fifth, we used prevalent cases for our evaluations. Recent research suggests that PRS accuracy may differ when predicting incident cases across various follow-up periods, potentially affecting PRS performance trends.^104^

This study also has limitations in scope. It includes only six diseases. Although we considered additional conditions (e.g., chronic obstructive pulmonary disease, Crohn disease, hypertension), data availability was insufficient to support rigorous analysis. Future works could investigate whether the trends observed here generalize to other diseases and quantitative traits. Finally, our analysis focused on PRSs derived from GWASs that included predominantly European ancestry participants. This limitation was dictated by the current imbalance in genetic study data across populations.^105^ The extent to which our results apply to other ancestries remains uncertain. However, our analysis of UKB participants of non-European genetic ancestry (Figure 2B), along with recent work on PRS portability,^61^^,^^79^ suggests that similar trends are likely. Nonetheless, assembling GWASs of large sample sizes in non-European populations remains essential. PRS performance is currently much lower in these groups, and increasing their representation is crucial for improving prediction.^105^

## Data and code availability

Individual-level data from the UKB were accessed from the UKB Resource under application nos. 42260 and 66995. Individual-level data from FinnGen were conducted by coauthors from the University of Helsinki with privileged access.

All GWAS summary statistics have been downloaded from publicly available websites including dedicated page from consortia, the NHGRI-EBI Catalog of human genome-wide association studies, and the FinnGen GWAS repository.

CDC disease prevalence: https://www.cdc.gov/datastatistics/index.html

FinnGen results: https://risteys.finngen.fi

Functional annotations: https://github.com/gkichaev/PAINTOR_V3.0/wiki/2b.-Overlapping-annotations

GWAS catalog: https://www.ebi.ac.uk/gwas

HapMap3: https://www.sanger.ac.uk/resources/downloads/human/hapmap3.html

All analyses were conducted using existing open-source software programs, which are freely available from the following URLs:

GCTA: https://yanglab.westlake.edu.cn/software/gcta/

GENESIS: https://github.com/yandorazhang/GENESIS

LDpred2: https://privefl.github.io/bigsnpr/articles/LDpred2.html

LDSC regression: https://github.com/bulik/ldsc

MiXeR: https://github.com/precimed/mixer

SBayesS: https://cnsgenomics.com/software/gctb/

sumHer: https://dougspeed.com/sumher

During the preparation of this work, the authors used GPT-4o to correct grammatical and syntax errors. After using this tool, the authors reviewed and edited the content as needed and take full responsibility for the content of the publication.

## Acknowledgments

We want to acknowledge the participants and investigators of the FinnGen study and the UKB cohort. This research has been conducted using the UKB Resource under application nos. 42260 and 66995. We also would like to thank the authors of GENESIS, sumHer, and SBayesS for their helpful recommendations. This work has been conducted as part of the INCEPTION program (Investissement d’Avenir grant ANR-16-CONV-0005). This research was supported by the 10.13039/501100001665Agence Nationale de la Recherche (grant nos. ANR-20-CE36-0009-02 and ANR-20-CE15-0012-01).

## Author contributions

H.A. and L.H. conceived and supervised the project. L.H., J.K., Z.Y., S.R., and G.P. carried out the primary analyses. C.A., C.B., H.J., A.F., and Y.S. conducted the secondary analyses. Z.Y. and A.G. led the analyses involving individual-level data from FinnGen. S.R., O.D., and L.H. led the analysis involving individual-level sequencing data from the UKB. L.H., F.P., B.V., C.A., and H.A. designed the LDpred2 pipeline for PRS analysis. H.A. and L.H. co-wrote the manuscript, with input from all other authors. All authors contributed to discussions.

## Declaration of interests

B.V. serves on Allelica’s international advisory board.

## Supplemental information

Data S1. Tables S1–S10

Document S2. Article plus supplemental information
