# Supplementary material for: Polygenic risk score prediction accuracy convergence
Source: HGG Adv. 2025 May 14;6(3):100457. doi: 10.1016/j.xhgg.2025.100457 (PMC12167061; doi:10.1016/j.xhgg.2025.100457)
Supplement: Document S1. Supplementary methods and Figures S1–S20 [file mmc1.pdf]

HGGA, Volume *6*

Supplemental information

Polygenic risk score prediction accuracy convergence

Léo Henches, Jihye Kim, Zhiyu Yang, Simone Rubinacci, Gabriel Pires, Clara Albiñana, Christophe Boetto, Hanna Julienne, Arthur Frouin, Antoine Auvergne, Yuka Suzuki, Sarah Djebali, Olivier Delaneau, Andrea Ganna, Bjarni Vilhjálmsson, Florian Privé, and Hugues Aschard

Contents

Supplementary Methods 2
- Existing AUC from the literature 2
- Additional GWAS within UK Biobank 2
- Phenotype selection in UK Biobank and FinnGen 2
- Correlation between European and FinGenn GWAS 3
- Specificity of the genetic model for obesity 3
- Specificity of the genetic model for Alzheimer disease 3
- Parameters estimation from GWAS using existing tools 4
- Simulations to study the expected convergence of PRS 5
- Estimation of alpha for common diseases using UK Biobank individual-level data 6
- Attenuated alpha model 6

Supplementary Figures 7
- Figure S1. European and FinnGen GWAS 7
- Figure S2. AUC as a function of sample size for 6 UK Biobank populations 8
- Figure S3. Variability in Alzheimer prediction conditional on APOE 9
- Figure S4. AUC based on PRS derived from alternative methods 10
- Figure S5. Prediction accuracy measured by alternative metrics 11
- Figure S6. Sample overlap and overfitting 12
- Figure S7. AUC convergence using simulated causal variants 13
- Figure S8. AUC increase, heritability and number of causal variants derived from GENESIS 14
- Figure S9. SBayesS disease parameters estimates across the six outcomes and GWAS 15
- Figure S10. Assessing the impact of GWAS heterogeneity on disease parameter estimation and AUC . 16
- Figure S11. AUC across CAD phenotypes 17
- Figure S12. Baseline and attenuated alpha models and proportion of heritability captured 18
- Figure S13. Variance of untyped variants captured by genotyped variants 19
- Figure S14. Variance explained by MAF bins 20
- Figure S15. Estimation of alpha using UK Biobank individual-level data 21
- Figure S16. Validation of the GRM-GCTA pipeline using simulated data 22
- Figure S17. Impact of prevalence on estimated maximum achievable AUC 23
- Figure S18. Correlation between functional annotations 24
- Figure S19. Distribution of imputation quality across GENCODE annotations 25
- Figure S20. Top annotations associated with imputation quality 26

References 27

Supplementary Methods

Existing AUC from the literature

We conducted in October 2023 a *PubMed* search for studies reporting the prediction accuracy of genetic risk score for six outcomes, coronary artery disease (CAD), breast cancer (BRCA), type 2 diabetes (T2D), Alzheimer disease (AD), asthma, and obesity using the disease name and the terms "*AUC*" and "*genetic risk score*", which we completed with *ad hoc* search terms. We conducted a first screening and filtered out irrelevant studies not reporting AUC. For the remaining ones, we carefully checked the analysis pipeline, and kept studies using i) at least 300 cases (the number of controls was always larger than the number of cases), ii) using mostly participants of European ancestry, and iii) where the AUC was derived using the genetic risk score only, excluding other covariates (*e.g.,* age, sex, etc). After this careful quality control, a total of 10, 23, 26, 6, 3 and 8 studies remained for CAD, T2D, BRCA, AD, asthma, and obesity, respectively. For each study we extracted the following parameters: the number of cases and controls of the training set used to derive the polygenic risk score, the number of genetic variants used, the reported AUC in the validation set, and the publication year. All collected information are reported in **Table S1**. Trends in AUC increase as a function of sample size was tested using a weighted linear regression as implemented in the R *lm()* function, where weights were defined as the logarithm of the effective sample size ($N_{eff} = 4\,N_{cases} \times N_{controls}\,/(N_{cases} + N_{controls})$).

Additional GWAS within UK Biobank

Consortium-based GWAS provided results for large sample size for each disease considered in this study (**Table S2**). However, we found only limited publicly available GWAS results with intermediate sample size for multiple outcomes. To complete our prediction accuracy analyses, we conducted five additional GWAS in the UK Biobank using unrelated participants of European ancestry, and cases sampled from the entire cohort: breast cancer (N_{cases}=5,000; $N_{controls}$=50,000); coronary artery disease (N_{cases}=5,000; $N_{controls}$=50,000); asthma (N_{cases}=1,000; $N_{controls}$=10,000); and BMI (N=20,000 and N=80,000). We also conducted a GWAS for obesity from the whole sample, where obesity was defined as BMI>30 (N_{cases}= 74,660 ; $N_{controls}$=238,048) for comparison purposes. All analyses were conducted using Plink2.0's --glm option and adjusted for the top 15 principal components[1], age, and sex. The GWAS only included the ~1M variants from HapMap3 used in the construction of the PRS scores. For breast cancer, we selected female cases and controls and adjusted for 15 PCs and age. For each study, we conducted a QC filtering using the default value of LDpred2 (https://privefl.github.io/bigsnpr/reference/snp_plinkQC.html). We removed all variants with a minor allele frequency (MAF) below 0.01, or Hardy-Weinberg equilibrium exact test p-value below 10^{-50}, and filtered out all SNPs and individuals with missing data over 10%.s

Phenotype selection in UK Biobank and FinnGen

For all analyses involving the UK Biobank and the FinnGen cohorts, we had to select a specific variable from all available fields for each of the six outcomes considered: type 2 diabetes (T2D), breast cancer (BRCA), coronary artery disease (CAD), Alzheimer disease (AD), asthma (AS), and body mass index (BMI)/obesity. In the FinnGen (https://risteys.finngen.fi/), we used the broadest definition of prevalent disease cases available. For type 2 diabetes, we used *Type 2 diabetes, definitions combined* (ID=T2D). For breast cancer, we used *Malignant neoplasm of breast* (ID=C3_BREAST). For CAD, we used *Major coronary heart disease event* (ID=I9_CHD). For Alzheimer, we used *Alzheimer disease* (ID=G6_ALZHEIMER). For asthma, we used *Asthma* (ID=J10_ASTHMA). For obesity, we used *Obesity* (ID=E4_OBESITY). In the UK Biobank (https://biobank.ndph.ox.ac.uk/), phenotypes were defined based on clinical ICD10 diagnosis codes (variable ID=41270) for five outcomes: breast cancer (ICD10 code=C50), coronary artery diseases (ICD10 codes=I21-I25), obesity (ICD10 code=E66), T2D (ICD10 codes=E10-E14), and asthma (ICD10 codes=E10-E14). For Alzheimer disease, we used AD-by-proxy (code 10 for variable ID=20110 from the mother and variable ID=20107 from the father), as proposed by Janssen et al[2], because the sample size for the Alzheimer disease

status was too small. Counts of cases and controls for each outcome and each population considered are provided in **Table S3.**

Correlation between European and FinGenn GWAS

The GWASs we used to derive the PRS include a vast majority of individuals of European descent (**Table S2**). Although the Finns population from the FinGenn cohort are also of European descent, they have a slight north Asian admixture[3]. To explore possible PRS portability issues[4], we compared top association results from the largest GWAS[5-8] from **Table S2** with those from the FinnGen GWAS, publicly available on their website (https://www.finngen.fi/en/access_results). For the latter, we used summary statistics from release 5, using IDs listed in the previous section. For each outcome we extracted from the corresponding European and Finns GWAS pair, all SNPs with a *p*-value below 1e-6 in either study. We created a merged list of variants and kept only those for which a *p*-value was available in both GWAS. We next clumped this subset using the *ld_clump()* function from the R package "ieugwasr" (https://rdrr.io/github/MRCIEU/ieugwasr/man/ld_clump.html) with an R^2 threshold of 0.05 and default options, and the 1000 genomes[9] as a reference panel. Using either the European *p*-values or the FinnGen *p*-values for clumping produced qualitatively similar results. Overall, when plotting the estimated regression coefficient from each pair of studies against each other, we found highly consistent results between FinnGen and European GWAS (**Figure S1**).

Specificity of the genetic model for obesity

Along the study we use body mass index (BMI) GWAS to build polygenic risk scores and estimate disease parameters for obesity (defined as BMI ≥ 30 kg/m2), because the sample size available for this phenotype is substantially higher than for GWAS of obesity. In theory, the heritability of BMI should equal the heritability of obesity on the liability scale, and the disease parameters should be fairly similar. Indeed, obesity follows the definition of the liability threshold model (LTM) that is commonly used to report the heritability of binary outcomes[10], with BMI being the liability. Briefly, the liability describes the combined risk of genetic and environmental factors that contribute to the development of a disease, with the disease status being determined by the liability reaching a threshold. We did not conduct a comparison between the two outcomes because of the lack of data for obesity, however, we did notice differences in some parameter estimations when using either BMI or obesity (e.g. **Fig. S15**), that might be investigated in future studies.

Note that for the derivation of the predicted AUC of obesity using GENESIS (see next section), we used the BMI GWAS, while the approach is expecting z-score derived from a logistic regression. We investigated the possible impact of using BMI GWAS as a proxy for obesity GWAS through simulations. We generated series of replicates where a normally distributed outcome Y depends on a single predictor X, and applied for each replicate a linear model $Y \sim \beta_0 + \beta_Y X$ and a logistic regression $logit(\Pr(D)) \sim \alpha + \beta_D X$, where D is a dichotomized version of Y, defined as $D = 1$ if Y is larger than the 70th percentile of Y and $D = 0$ otherwise (mimicking the BMI-obesity relationship). We compared the z-scores from the two models $z_Y = \hat{\beta}_Y / \sigma_{\hat{\beta}_Y}$ and $z_D = \hat{\beta}_D / \sigma_{\hat{\beta}_D}$ and found strong correlation ($r^2 > 0.9$ across the scenarios considered), but with z_D being systematically smaller than z_Y, with on average $z_D = 0.73\, z_Y$, corresponding approximately to a two-fold decrease in sample size for the dichotomized outcome. We used this *ad hoc* correction factors when plotting the obesity GENESIS results (**Fig. S8**).

Specificity of the genetic model for Alzheimer disease

Genetic variants within the Apolipoprotein E (ApoE) gene encode for three haplotypes, ε2, ε3 and ε4 that are critical determinants of Alzheimer disease risk[11]. Those variants with large effect deviate from the standard polygenic model and are commonly assessed separately from the effect of other variants. The causal APOE variants were available in the three largest Alzheimer disease GWAS (Lambert et al. 2013, Kunkle et al. 2019 and Jansen et al. 2019), but absent from the smallest one (Li et al. 2008), thus potentially impacting the comparison of the predictive performance. Moreover, the LDpred2[12] approach used to derive the polygenic risk score (PRS) can be sensitive to such deviation of the polygenic model. To address this possible limitation,

in complement to the standard pipeline, we derived three additional predictive models: a PRS excluding the APOE region (PRS.noAPOE), a risk score based on APOE ε2 and ε4 allele genotypes (rs429358 and rs7412) with effect estimates pulled from Kunkle et al[13] (the largest GWAS for AD status) (PRS.APOE), and a PRS including PRS.noAPOE and PRS.APOE as two distinct components. The predictive performances from these alternative PRSs are presented in **Figure S3**. Also, note that when using the PRS excluding APOE in **Figure 3**, we used the twin heritability estimates after subtracting the contribution of APOE variants, which has been reported to be approximately 9% of the total phenotypic variance[14].

Another challenge in the analysis of Alzheimer disease is the use of the so-called AD-by-proxy instead of the AD status in the largest GWAS (Jansen et al[2]). AD-by-proxy is a score determined by the Alzheimer status of the parents' participants. It has been proposed to virtually increase the available sample size in AD GWAS and therefore increase statistical power. In our estimation of the AUC in the FinnGen cohort, we use the true AD status. This might potentially explain the limited increase in AUC in that cohort when using the Jansen et al[2] PRS. Conversely, in the estimation of the AUC in the non-European UKB participants, we used the AD-by-proxy as the primary outcome because of a limited sample size for the true AD status (N=961 for AD-by-proxy cases, and N=65 for AD case). For that cohort, the AUC show a substantial increase for the Jansen et al PRS, likely due to the outcome correspondence. While a full investigation of the impact of using AD-b-proxy is out of the scope of this study, we derived the predictive power of the APOE variants (PRS.APOE) in the six non-European UK Biobank populations as a qualitative marker of this proxy phenotype. We obtained AUC=0.65 (SD=0.58) and AUC=0.54 (SD=0.52), for the AD status and the AD-by-proxy status, respectively. This is in line with the expectation that a loose disease definition can impact the predictive power of genetic risk score.

Parameters estimation from GWAS using existing tools

We used multiple tools to estimate disease genetic parameters from GWAS summary statistics. We applied GENESIS[15], SBayesS[16], sumHer[17], LDSC[18], and MiXeR[19,20] to all GWAS from **Table S2** except for Saxena et al 2007 and Li et al 2008 because of modest sample size (N_{eff}=2,931 and 1,489, respectively) (**Table S6** and **Figs. S8-S10**). All methods estimate heritability. GENESIS, SBayesS, and MiXeR additionally estimate the number of causal variant and the polygenicity. SBayesS and sumHer also provide estimates of α, the MAF-effect size relationship parameter. GENESIS output was also used to derive the expected trend of the AUC as a function of sample size (**Fig. S8**). We parametrized the method following the available tutorials and direct recommendations from the authors. Note that those methods sometimes refer to parameters that have different definition or labels (e.g. α is sometimes referred to as S, effective sample size is defined differently across methods, the output heritability scale is heterogeneous, etc). When possible, we harmonized the output estimates to allow for a direct comparison across methods. Below is a brief description of the overall analysis pipeline and specific parametrization of each method.

All analyses were conducted using GWAS variants overlapping with a reference set of 1,054,330 variants from HapMap3[21]. Most of these methods assume the original GWAS have not been corrected for chi-squared inflation using genomic control (GC)[22]. We carefully read each GWAS paper to assess whether GC correction was applied. The correction strategy across GWASs was heterogeneous (**Table S2**), sometimes applied at the study level (by each study, before the meta-analysis), or at the meta-analysis stage, at both, or neither. The information was also sometimes missing, or partial (a correction was reported, but the actual correction factor was not provided). We identified three studies were the inflation factor λ_{GC} use for correction was fairly large (Nikpay et al. 2015, Speliotes et al. 2010, and Locke et al. 2015) and we back-corrected the GWAS results before the analysis.

<u>GENESIS</u> produces estimates of the SNP heritability, the number of causal variants, and allows to generate expected AUC as a function of the sample size. Following recommendations from the authors, we ran a three-component mixture model, which assumes that the effect sizes for susceptibility variants can be described by two distinct normal distributions (small and large effects) and a third component corresponding to non-associated variants. We use as input the effective sample size derived as $N_{eff} = N_{cases} \times N_{controls}$ / $(N_{cases} + N_{controls})$, but report the results using the alternative derivation $N_{eff} = 4\, N_{cases} \times N_{controls}$ /

($N_{cases} + N_{controls}$). The heritability estimated in GENESIS is on the log-odds scale and was transformed to the liability scale using the formula $h_l^2 = h_{log}^2 K^2(1-K)^2/z^2$, where K is the disease prevalence in the population and z the height of the standard normal probability density function at the liability threshold[10].

SBayesS produces estimates of the number of causal variants, the SNP heritability and α. We used the shrunk LD matrices proposed along the software. It requires the frequency of the coded allele, which was not available for several GWAS. Instead, we used frequencies derived from participants of European ancestry in the 1KG reference panel[9]. For all analyses we used the options *--exclude-mhc* to remove the MHC region from the derivation, and *--impute-n* that re-derive the sample size, as the per-variant sample size is likely heterogeneous for many of the GWAS analysed. This option also filters out variants that have sample size 3 standard deviation away from the expected. For the sample size, we used the total sample: $N = N_{cases} + N_{controls}$. Heritability on the liability scale was derived in a second step using the formula[10]: $h_l^2 = h_{obs}^2 \left(K(1-K)\right)^2/(P(1-P)z^2)$, where K and P are the population and in-sample prevalence, respectively.

MiXeR[19,20] produces estimates of the fraction and number of causal SNPs and the SNP heritability, based on GWAS summary statistics and assuming a Gaussian mixture model. MiXeR takes into account SNP heterozygosity, LD structure, and residual inflation of z-scores due to variance distortion (which can arise from cryptic relatedness in the sample). We used the default reference panel, the 1000 Genomes Phase3 data and the MHC region was excluded from the analysis. As recommended by the authors, we used the standard definition of the effective sample size: $N_{eff} = 4\, N_{cases} \times N_{controls}/(N_{cases} + N_{controls})$. Heritability on the liability scale was derived in a second step using the formula[10]: $h_l^2 = h_{obs}^2 \left(K(1-K)\right)^2/(P(1-P)z^2)$, where K and P are the population and in-sample prevalence, respectively.

LDSC regression[18] was used to derive estimates of SNP heritability. Here we used the original model proposed in 2015 by Bulik-Sulivan et al[18], which has been widely used in the field. Heritability is derived from a regression between the chi-squared for association and the LDscore, a metric that quantifies the strength of the correlation between each variant and its neighbours. The standard model implicitly assumes an infinitesimal model with an equal per-variant contribution to the heritability. For the sample size, we used the total sample: $N = N_{cases} + N_{controls}$. Heritability on the liability scale was derived in a second step using the formula[10]: $h_l^2 = h_{obs}^2 \left(K(1-K)\right)^2/(P(1-P)z^2)$, where K and P are the population and in-sample prevalence, respectively.

sumHer[17] is an extension of the LDAK model[23] for summary statistics. As compared to LDSC, sumHer uses a model were per-SNP heritability varies with both linkage disequilibrium (LD) and minor allele frequency (MAF). We used the options *--cutoff* 0.01 to remove variants that explain more than 1% of phenotypic variance, and specified the in-sample and population prevalence with *--prevalence* and *--ascertainment*, so that the heritability is directly derived on the liability scale. For the sample size we used $N = N_{cases} + N_{controls}$. For the estimation of α we used the solution proposed within sumHer that consists in testing a range of α, and estimating the best fit (i.e. the highest log-Likelihood) using the *--find-gaussian* option.

Simulations to study the expected convergence of PRS

To characterize the convergence of PRS prediction as a function of the disease parameters and sample size, we simulated series of matched case/control (i.e. $n = n_{case} = n_{control}$) replicates varying the disease prevalence (K = [0.01; 0.25], the disease heritability (h^2 = [0.2; 0.7], the number of causal genetic variants (M = [100, 1000, 5000, 10,000], and the sample size used in the GWAS (n = [250; 2,500; 25,000; 250,000]) (**Fig. S7**). Independent causal single nucleotide polymorphisms (SNP) were generated under a binomial distribution with minor allele frequency randomly drawn in [0.05, 0.5]. The disease status was generated under a liability threshold model. The effect of the SNPs on the liability were drawn from a gaussian distribution $\beta \sim \mathcal{N}(0, h^2/M)$, and the threshold defined as $\mathrm{T} = \Phi^{-1}(1-k)$ where Φ is the cumulative distribution function of the normal distribution. Each replicate was split in two subsets. The first subset was used to estimate genetic effect $\hat{\beta}$ for each of the $G_{i=1\ldots M}$ variants using univariate logistic regression. A PRS was then computed in the second subset as: $PRS = \sum_{i=1\ldots M} \hat{\beta}_i\, G_i$. The predictive performances of the PRS for each

model was derived as the average over 10 simulations of the Area under the ROC (AUC) curve in this second subset.

Estimation of alpha for common diseases using UK Biobank individual-level data

We derived the α parameter for the six outcomes using individual-level genetic and phenotypic data from the UK Biobank using the approach described by Schoech et al[24] (**Fig. S15-S16**). In brief, we derived $\mathbf{A}_\alpha$, the genetic correlation matrices (GRM) conditional on a pre-specific α values in [-1, 0], and defined as $\mathbf{A}_\alpha = \mathbf{X}\mathbf{D}_\alpha\mathbf{X}^t$, where $\mathbf{X}$ is the genotype matrix and $\mathbf{D}_\alpha$ is a diagonal matrix with element $D_{ii} = [2p_i(1-p_i)]^\alpha$. For each outcome Y, profile likelihoods $L(\alpha)$ of the multivariate normal model $Y \sim \mathcal{N}\left(0, \mathbf{A}_\alpha\sigma^2_{g,\alpha} + \mathbf{I}\sigma^2_\varepsilon\right)$, where σ^2_ε is the residual variance, were derived for a range of α value using the GCTA software[25]. The final $\hat{\alpha}$ estimate was derived as $\hat{\alpha} = argmax(L(\alpha))$. Estimation was done using up to 261,028 unrelated self-reported "white British" participants with genetic data for 17,211,988 variants with minor allele frequency (MAF) equal or larger than 0.01%, and *info_score* larger than 0.8.

In practice, the estimation of this parameter from individual-level data poses severe computational challenges, and the GRM-MAF-LD package proposed by Schoech et al[24] was not runnable in these data. We followed the same procedure, but re-implemented each step and optimized the parameters for computational purposes. For the derivation of $\mathbf{A}_\alpha$, we modified the GRM function from PLINK 2.0[26], to include the α weighting. Due to memory constraints, GCTA could not be run using a GRM of several hundred thousand individuals at once. To address this limitation, we divided the dataset into eight equally sized random sub-samples, and estimated $L(\alpha)_s$ for each subsample s using GCTA. The overall $L(\alpha)$ was then derived as the sum of the resulting log likelihoods: $L(\alpha) = \sum_{s=1\ldots8} L(\alpha)_s$. We considered a range of α values in [-1.3 ; 0], with 0.1 steps. Data points were then interpolated using a Locally Weighted Least Squares Regression as implemented in the R *loess()* function to obtain the final estimate of $\hat{\alpha}$. We validated the procedure using real genotype data from the UK Biobank cohort and simulated phenotypes (**Fig. S16a**). We also attempted to estimate alpha across the MAF strata. However, this estimation was unreliable, displaying slow convergence because of the limited variance within each MAF bin (**Fig. S16b**).

Attenuated alpha model

The alpha model is defined as $\beta_i | p_i \sim \mathcal{N}\left(0, \sigma^2_{g,\alpha} \cdot [2p_i(1-p_i)]^\alpha\right)$, where p_i is the minor allele frequency of variant i, and $\sigma^2_{g,\alpha}$ is a constant constraining the outcome heritability. For small value of alpha, it implies the per-allele effect of rare variants can be several orders of magnitude larger than for common variants (**Fig. S12a**). As noted in Schoech et al[24], the fit of the alpha model in evolutionary forward simulations is expected to hold above a fairly low MAF threshold (MAF>0.6% in the reported example), but might overestimate the effect of variants with MAF below that threshold. Let's denote θ the scaling factor of effect size coefficient, so that $\theta_i = [2p_i(1-p_i)]^\alpha$. To account for possible deviations of the alpha model, we considered attenuated effect size θ^*_i for very rare variants. We defined the attenuation using an inverse logit function with a tipping point at the aforementioned MAF threshold T=0.6%: $\pi = 1/[1 + exp(\log(MAF) - \log(T))]$. The attenuation was applied recursively to the beta coefficient ordered by decreasing MAF: $\theta^*_i = \theta^*_{i-1} + (\theta_i - \theta_{i-1})(1 - \pi w)$, where w is a weight in [0, 1] to set the strength of the attenuation. **Figure S12b** illustrates the effect of the proposed attenuation on the MAF-effect size relationship for $\alpha = -0.3$. We varied the weight w over [0.1, 0.3, 0.5, 0.7, 0.85, 0.95, 0.99] and repeated the experiment from **Figure 3a**, deriving the expected proportion of heritability captured across this range of attenuated alpha model using either r^2, the squared correlation between the sequence and imputed variants (**Fig. S13b**), or ρ^2, the squared correlation between the untyped variants j and the genotyped ones (**Fig. S13a**). When comparing those attenuated models against the baseline alpha model in real data, we did not find any evidence for an increased fit (**Fig. S15**).

Supplementary Figures

Figure S1. European and FinnGen GWAS

Beta coefficients at top associated independent variants from FinnGen GWASs and European ancestry GWASs for six outcomes: obesity (a), breast cancer (b), asthma (c), type 2 diabetes (d), Alzheimer disease (e), and coronary artery disease (f). Across all variants, squared-correlation equal 0.43 (obesity), 0.56 (breast cancer), 0.50 (asthma), 0.67 (type 2 diabetes), 0.55 (Alzheimer disease), and 0.66 (coronary artery disease). Note that for obesity the European GWAS results were extracted from a BMI GWAS, explaining the scaling difference in regression coefficient. For Alzheimer disease (AD), the European GWAS was derived using AD-by-proxy, inducing a larger variance in the outcome, and again difference in the scaling of the regression coefficient.

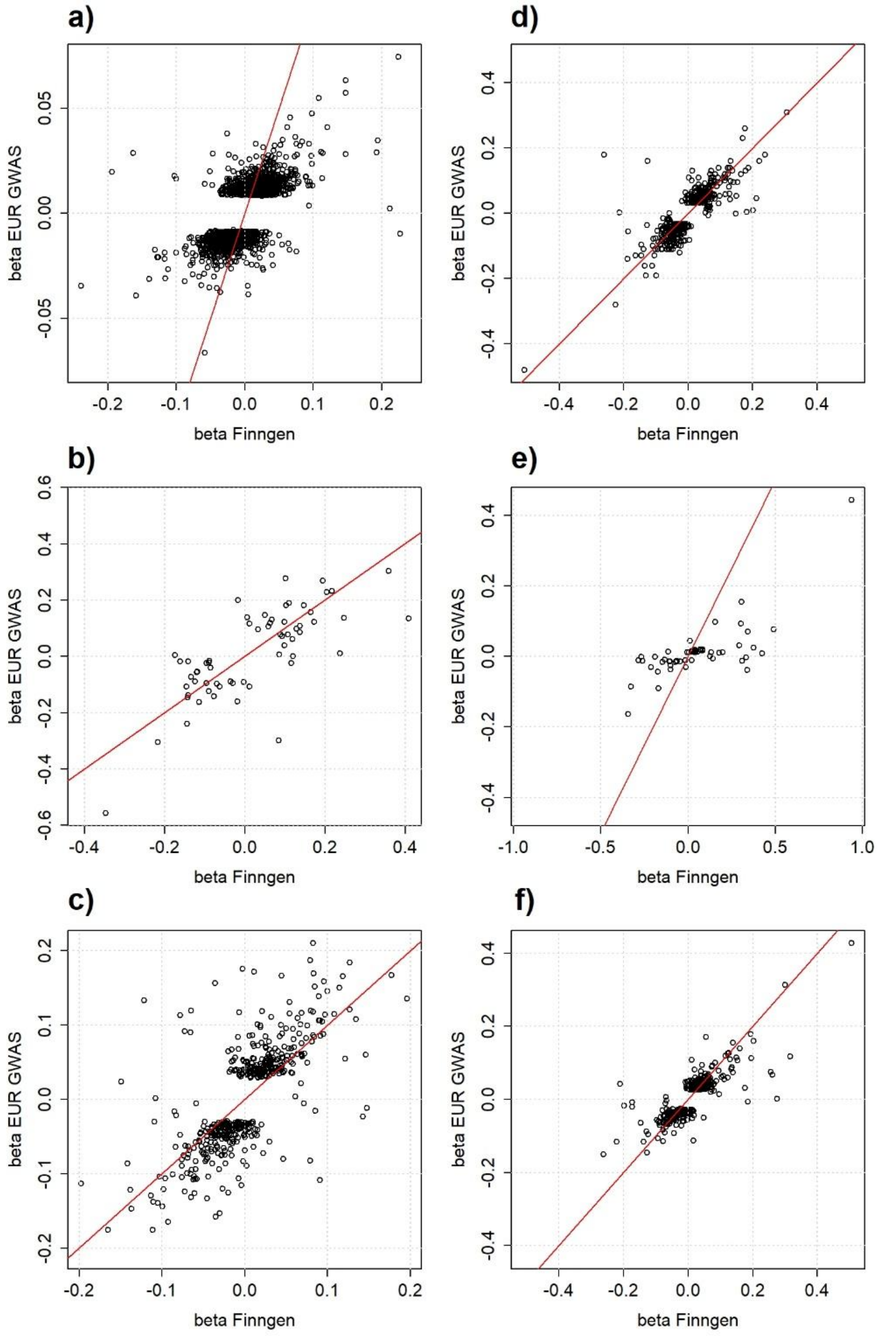

Figure S2. AUC as a function of sample size for 6 UK Biobank populations

AUC as a function of the effective sample size for each of the 6 ancestries analysed in the *UK Biobank* (Ashkenazi, Iranian, Indian, Chinese, Caribbean, and Nigerian). Grey areas correspond to the 95% confidence interval. AUC was derived for 6 outcomes: coronary artery disease (CAD), type 2 diabetes (T2D), breast cancer (BRCA), Alzheimer disease (AD), asthma, and obesity. Obesity was predicted using BMI GWASs. For AD, the outcome predicted was AD-by-proxy because of a limited number of AD cases within each ancestry. The PRS for this outcome was derived after excluding the APOE region.

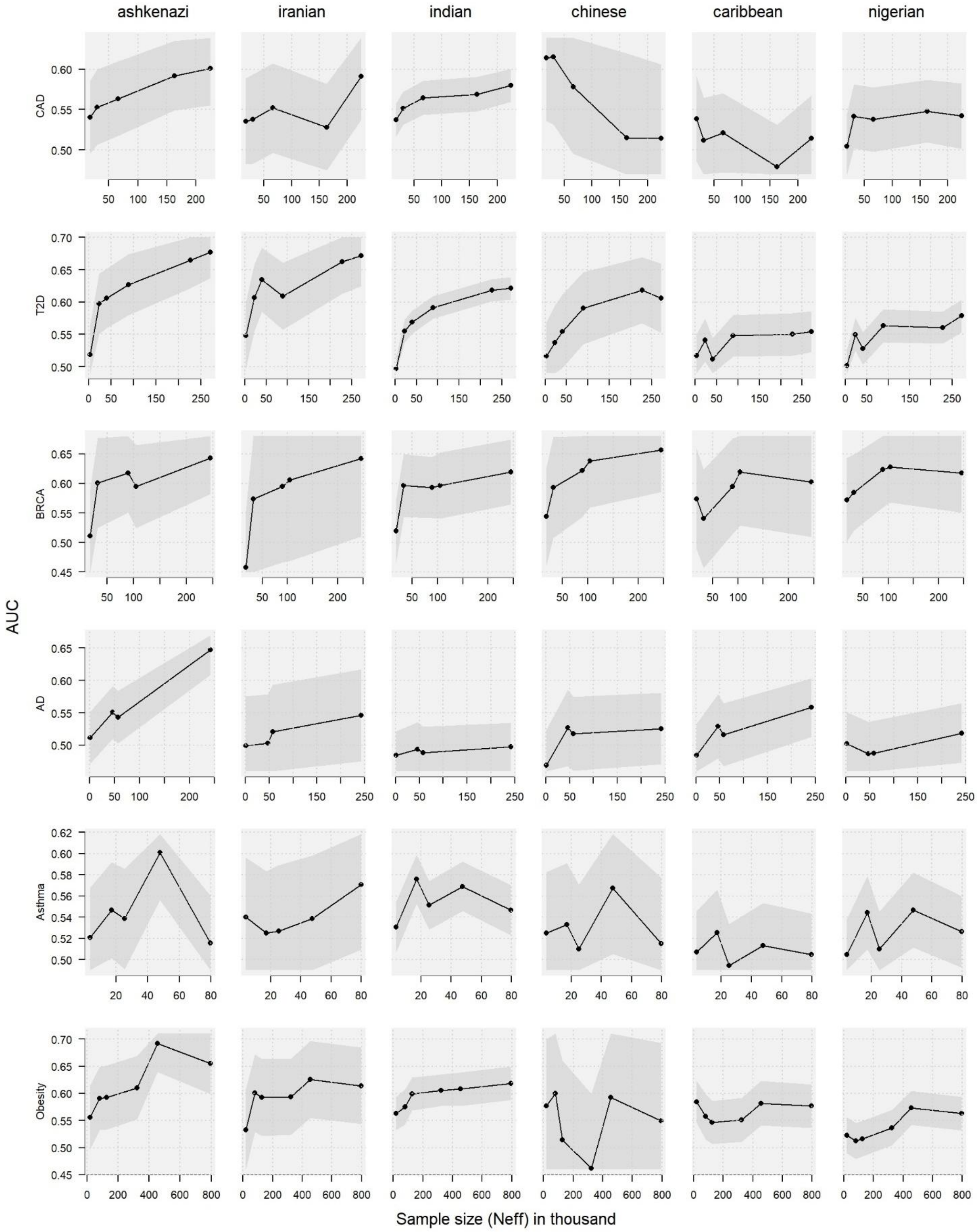

Figure S3. Variability in Alzheimer prediction conditional on APOE

For Alzheimer disease we considered three alternative genetic models conditional on the APOE region: i) a PRS derived after excluding the APOE region ("PRS excluding APOE", plain line), which was used as the main analysis, ii) a PRS derived using the same pipeline as for other diseases treating the APOE region as the rest of the genome ("*PRS including APOE region*", dash line), and iii) the combination of a PRS derived excluding the APOE region and score for the ε_1 and ε_2 variants as a distinct predictors ("*PRS and APOE treated separately and merged*", dotted line). The panels present the results of each of the three genetic scores in the FinnGen participants, and the non-European UK biobank participants per population and after a meta-analysis ("*UKB Combined*").

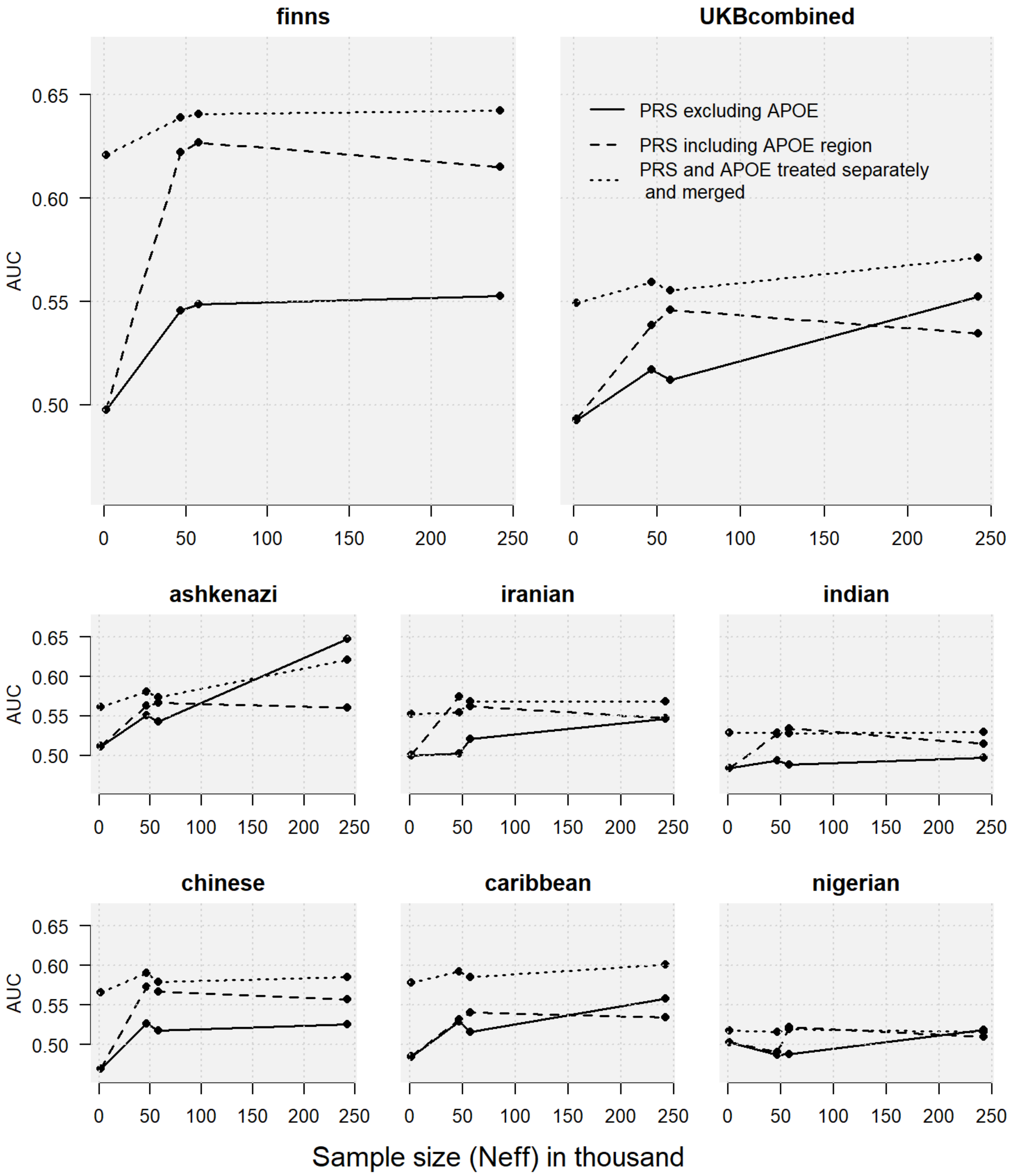

Figure S4. AUC based on PRS derived from alternative methods

AUC for the six outcomes derived using the same pipeline as in Figure 1b and alternative polygenic risk scores (PRS). PRS were trained from the 35 genome-wide association study (GWAS) using LDpred2, SBayesR, and a clumping + thresholding using a P-value threshold of 0.05, 10^{-4} and 10^{-8}. The PRS were tested in individual-level data from the FinnGen cohort. The AUCs are plotted as a function of the effective sample size of the corresponding GWAS.

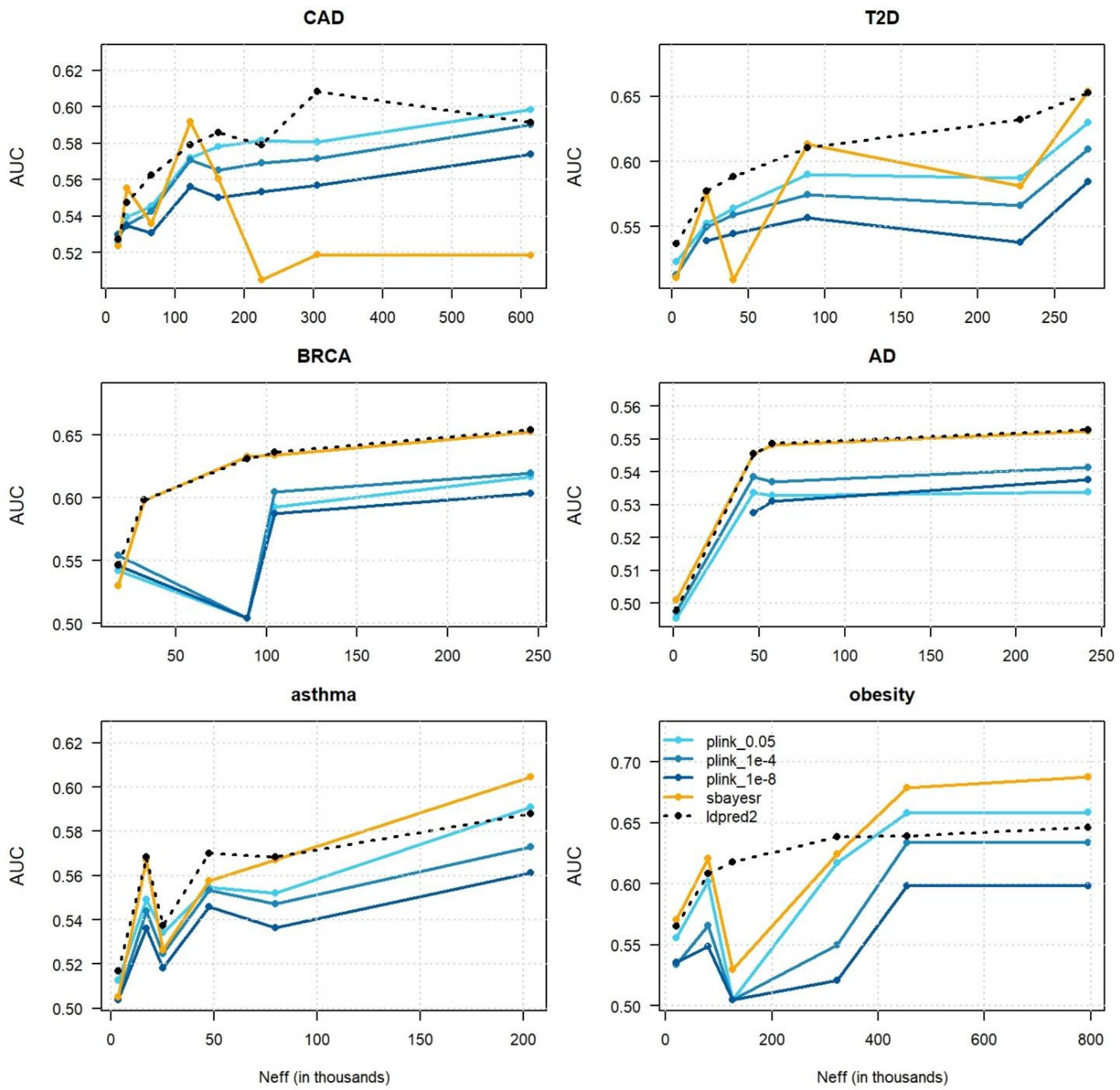

Figure S5. Prediction accuracy measured by alternative metrics

Prediction accuracy for the six outcomes measured by AUC, R2, and odds ratios (OR) of the disease comparing the top 5% and top 1% PRS stratum and the general population. Polygenic risk scores were trained from 35 genome-wide association study (GWAS) using LDpred2 and tested in individual-level data from the FinnGen cohort. All accuracy metrics are plotted as a function of the effective sample size of the corresponding GWAS. To facilitate the comparison of the trend across metrics, within each panel, we aligned the scale of each metric in order to match the maximum and minimum values. The three corresponding ordinates are presented for AUC (black), R2 (yellow), and OR (blue).

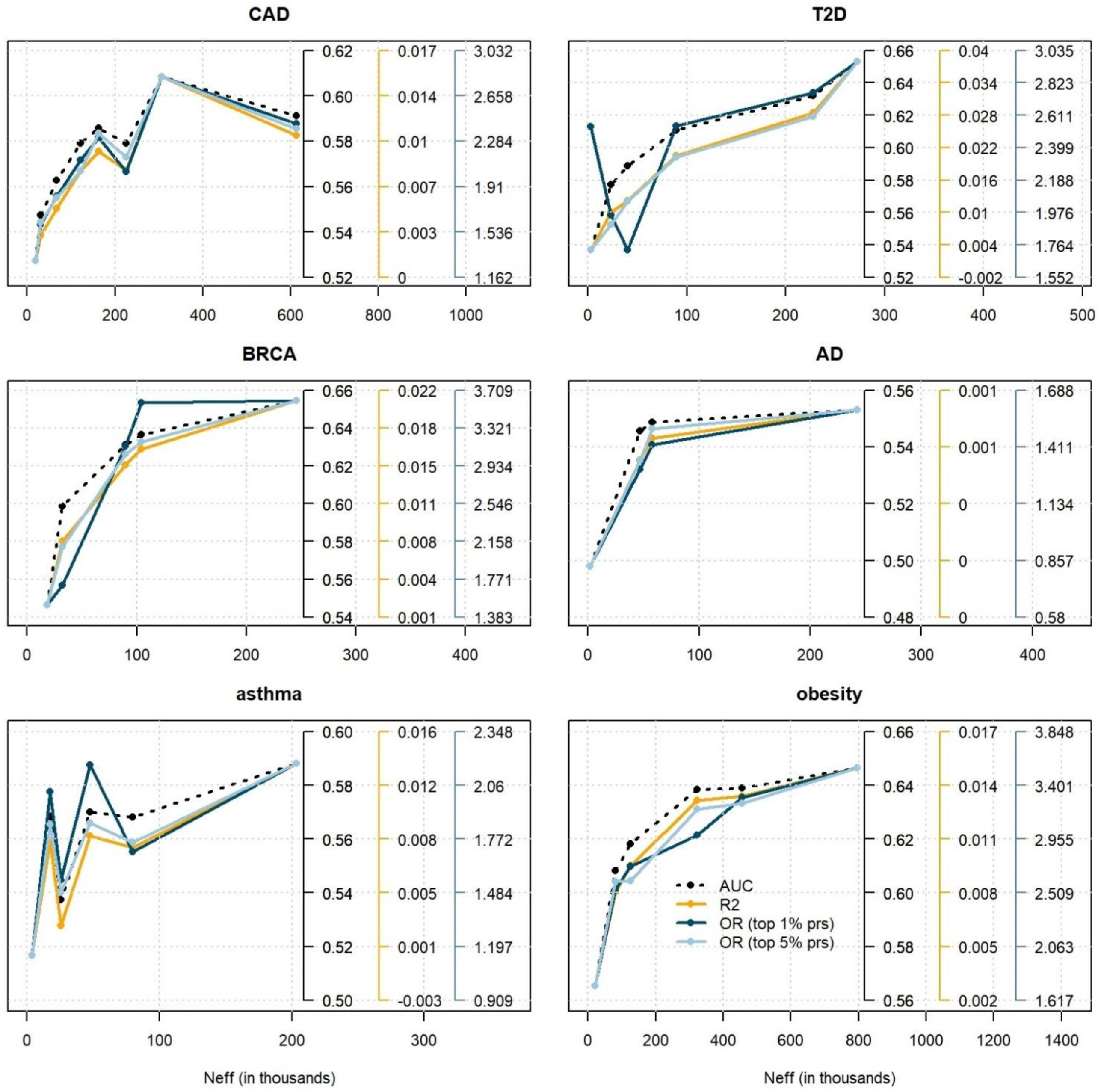

Figure S6. Sample overlap and overfitting

We investigated the impact of sample overlap in the prediction accuracy of PRS, as measured by the AUC, using data from the UK Biobank and BMI. We used a training set of 200,000 unrelated participants of European ancestry to build a polygenic risk score for BMI using LDpred2. We applied this PRS to predict obesity in 10,000 unrelated participants of European ancestry, while sampling the test set either independently from the training set, or considering a partial overlap with the training (20%, 40%, 60%, 80 and 100%). The left panel presents the AUC derived in the test set as a function of the sample overlap. The grey area represents the 95% confidence interval of the AUC. The right panel illustrates the sample overlap.

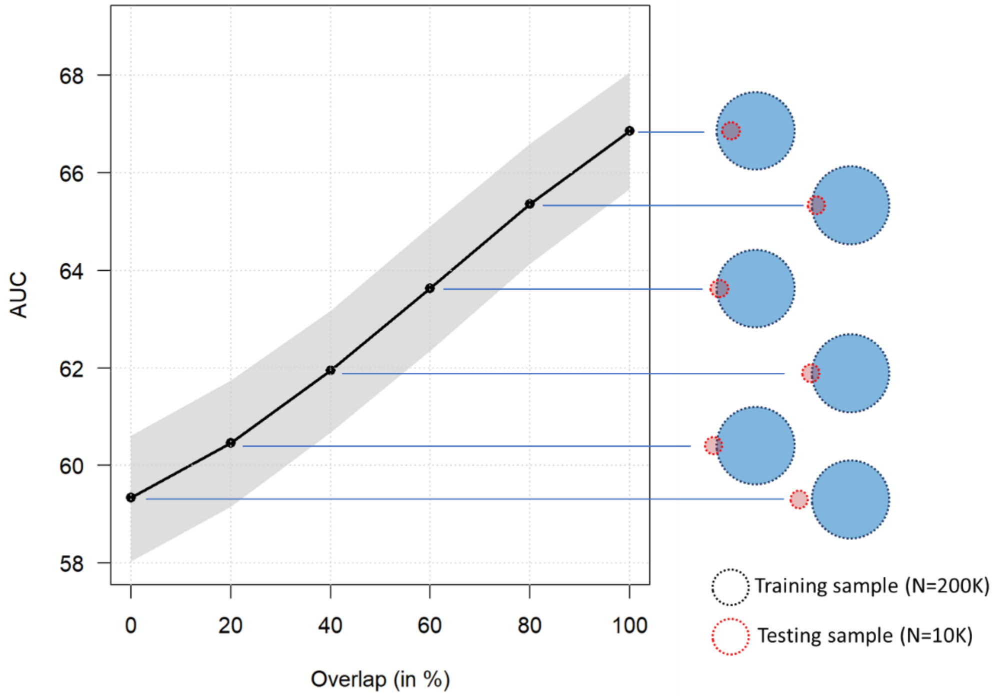

Figure S7. AUC convergence using simulated causal variants

Average predictive power as measured by the Area under the ROC (AUC) in simulated matched case/control replicates. Disease status was drawn under a liability threshold model using 100 to 10K independent causal variants. For each replicate, the effect of causal variants was estimated using univariate logistic regression using an increasing sample size (n = [250; 2,500; 25,000; 250,000]). A PRS was then computed in an independent subset using those estimates and its performance derived as the average AUC over 10 simulations. Results across low and high heritability (h_A^2) and disease prevalence (K) are presented in panel a) (h_A^2=0.2, K=0.25), b) (h_A^2=0.2, K=0.01), c) (h_A^2=0.7, K=0.25), and d) (h_A^2=0.7, K=0.01). The black dash line represents the expected maximum achievable AUC derived based on heritability and prevalence.

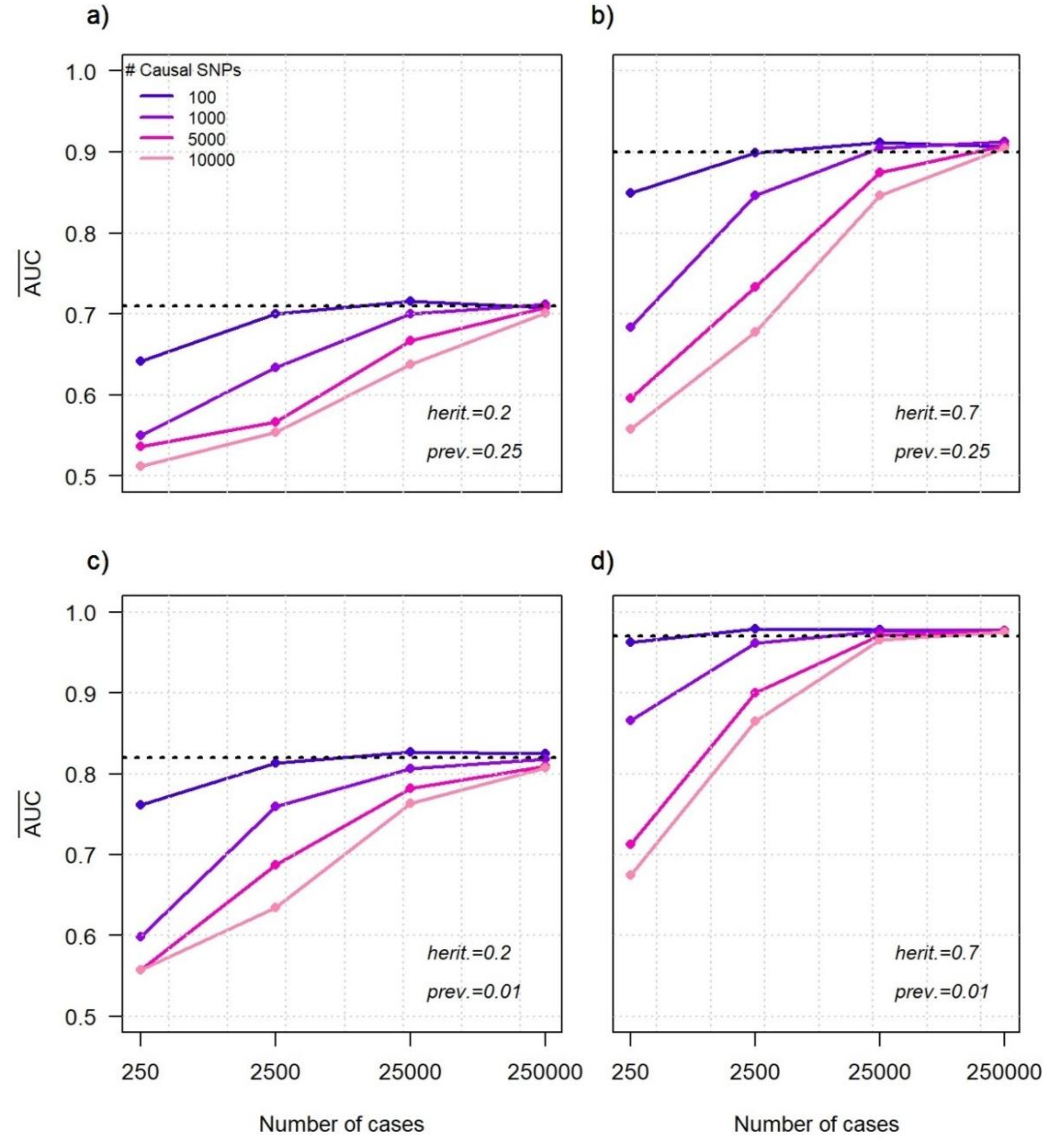

Figure S8. AUC increase, heritability and number of causal variants derived from GENESIS

Predicted AUC as a function of sample size derived from the GENESIS package assuming a 3-components genetic model, where variants are classified as either non-causal, causal with low effect, or causal with large effect. GENESIS was applied to all GWAS data form each of the six outcomes, coronary artery disease, breast cancer, type 2 diabetes, Alzheimer disease, asthma, and obesity. Note that for obesity, we used body mass index (BMI) GWAS and applied an *ad hoc* correction of the sample size, expected to match power of a binarized version of BMI. AUC derived in FinnGen and the UK Biobank are indicated in black and red, respectively(a). The predicted AUC are based on estimates of heritability (b) and the number of causal variants (c) estimated within GENESIS. Error bars indicate the 95% confidence interval. The shape of the predicted AUC as a function of sample size is reasonably close to the ones observed in FinnGen and the UK Biobank**,** however, the increase rate and expected maximum AUC varied substantially conditional on the input GWAS used.

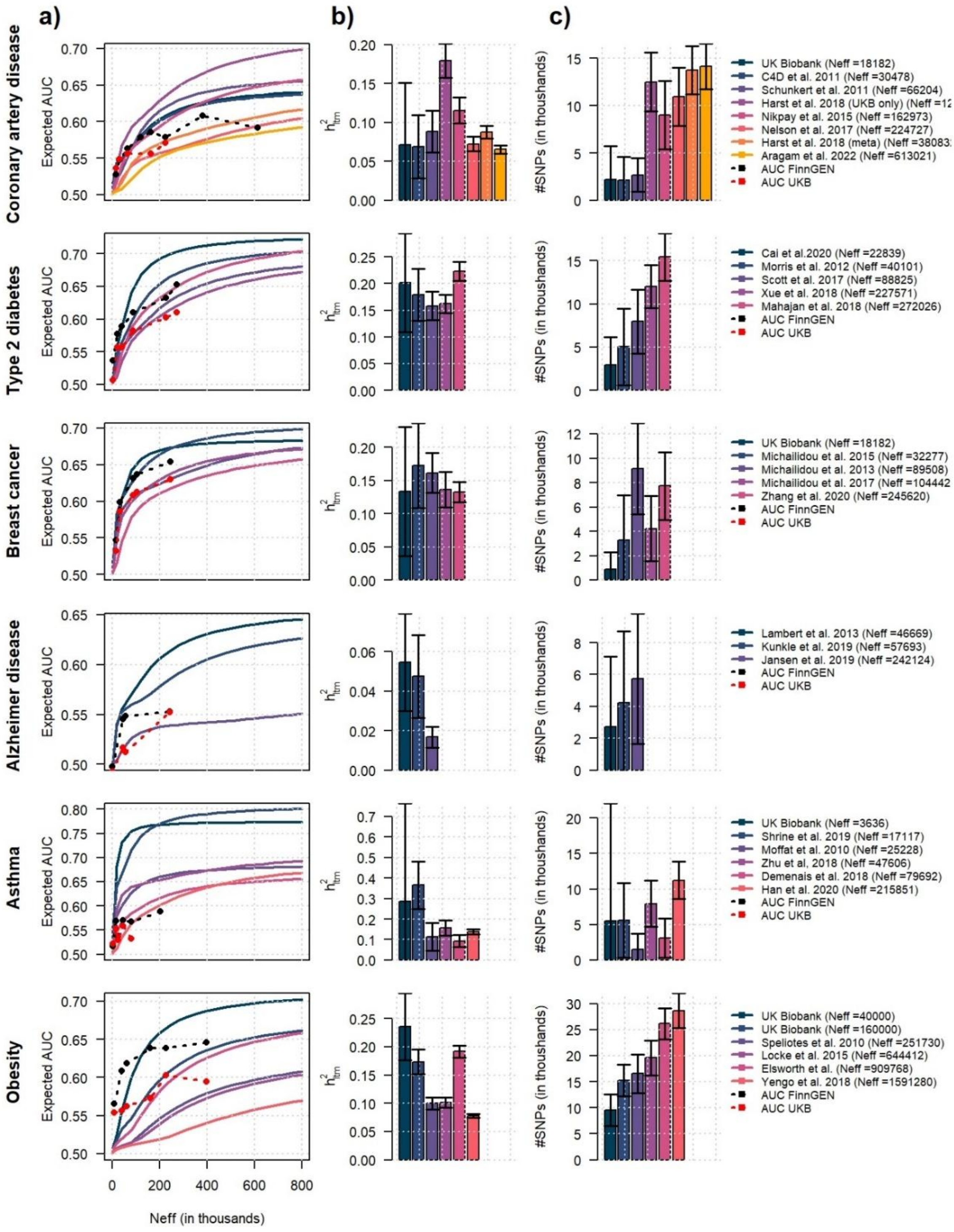

Figure S9. SBayesS disease parameters estimates across the six outcomes and GWAS

Estimates and 95% confidence interval of polygenicity (the proportion of causal variants), heritability and alpha derived using SBayesS across 31 GWAS summary statistics from six outcomes: coronary artery disease (CAD), breast cancer (BRCA), type 2 diabetes (T2D), Alzheimer disease (AD), asthma (AS), and body mass index (BMI). We only include GWAS with an effective sample size larger than 5,000. All analysis were conducted using a subset of approximately 1M hapmap3 variants.

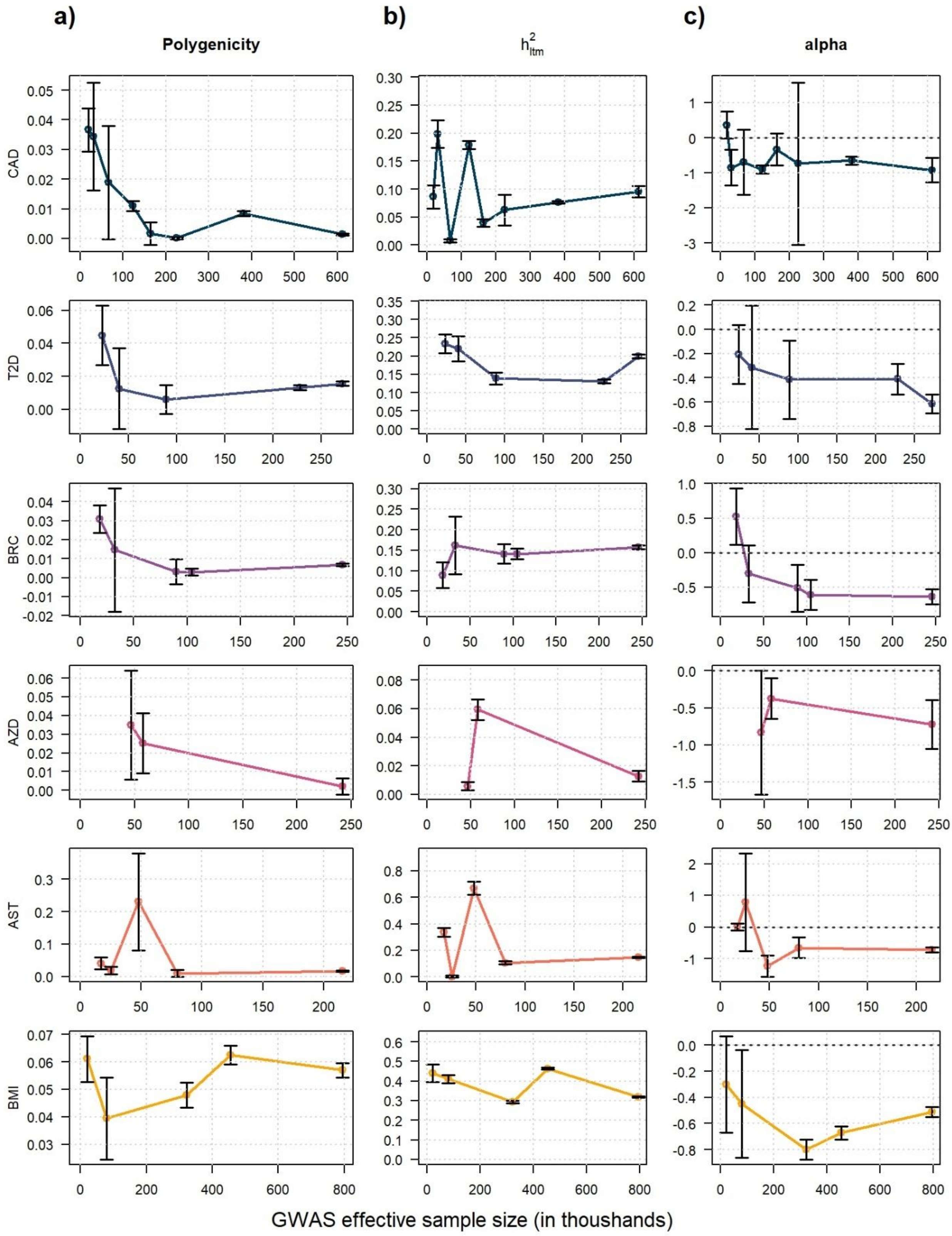

Figure S10. Assessing the impact of GWAS heterogeneity on disease parameter estimation and AUC

We conducted GWAS for body mass index (BMI) in random subsamples of the UK biobank with sample size N increasing from 20K to 300K. For each GWAS, we applied SBayesS to estimate polygenicity (the percentage of causal variants), alpha, and heritability under the liability threshold model. For each resulting GWAS, we applied LDpred2 to derived PRS following the same pipeline as for Figure 1, and applied these PRSs to derive the AUC in a holdout test dataset from the UK Biobank not used in the GWAS. We repeated the analysis five times with a different holdout subset for each analysis. Panel a), b) and c) present estimates of polygenicity, alpha and heritability across the five experiments as a function of sample size. Panel d) presents the average AUC derived in the UK Biobank holdout (blue). Results AUC from Figure 1b (obesity), derived in FinnGen ("Finns external") and in non-European participants from UKB ("UKB external") using PRS from external GWAS are presented in red and black, respectively.

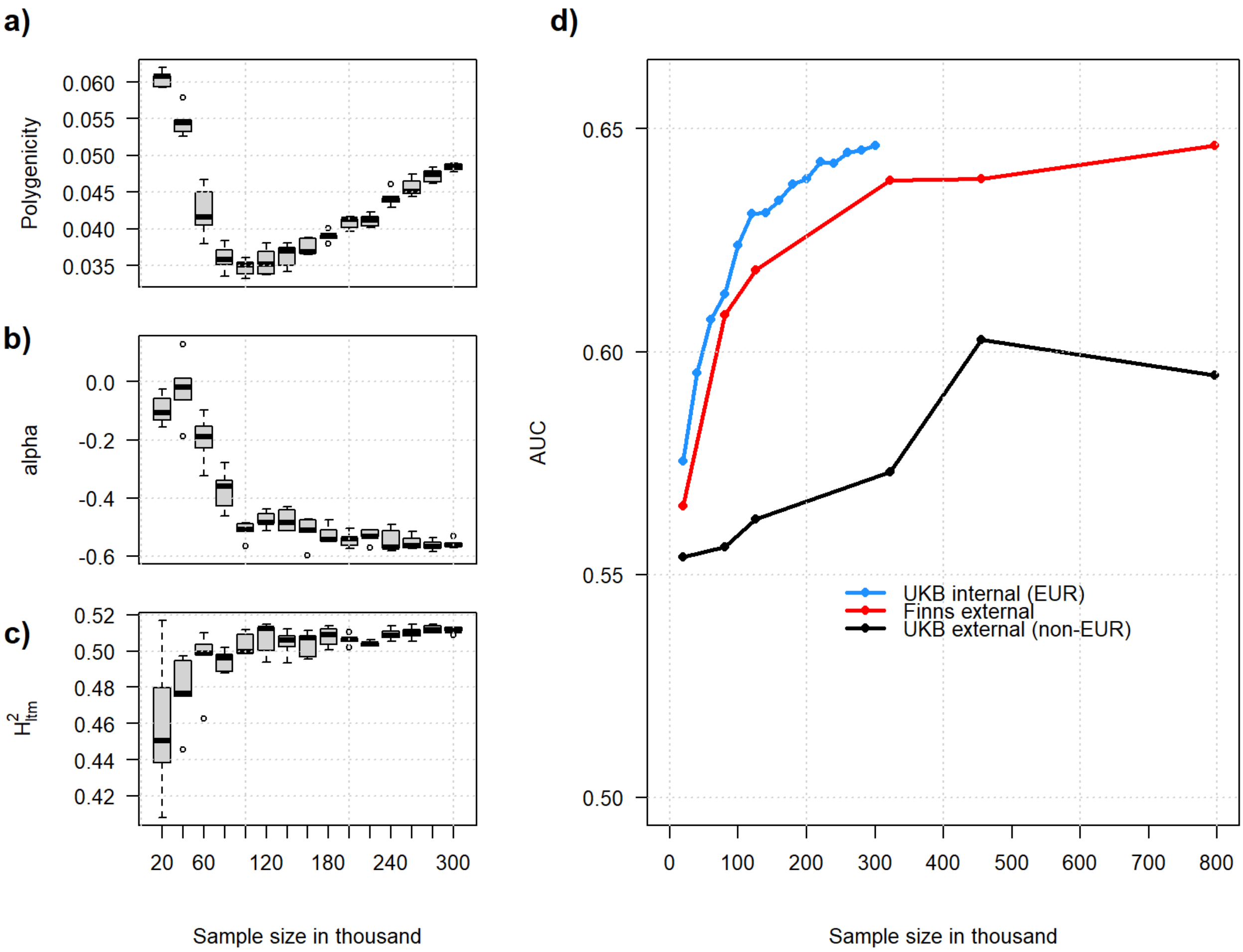

Figure S11. AUC across CAD phenotypes

AUC for seven CAD phenotypes derived using the CAD PRS in FinnGens: myocardial infarction (I9_MI), myocardial infarction, strict (I9_MI_STRICT), coronary revascularization (I9_REVASC), ischemic heart diseases (I9_ISCHHEART), coronary atherosclerosis (I9_CORATHER), coronary angioplasty (I9_ANGIO), angina pectoris (I9_ANGINA). Right panel presents the number of cases in the FinnGen cohort for each phenotype

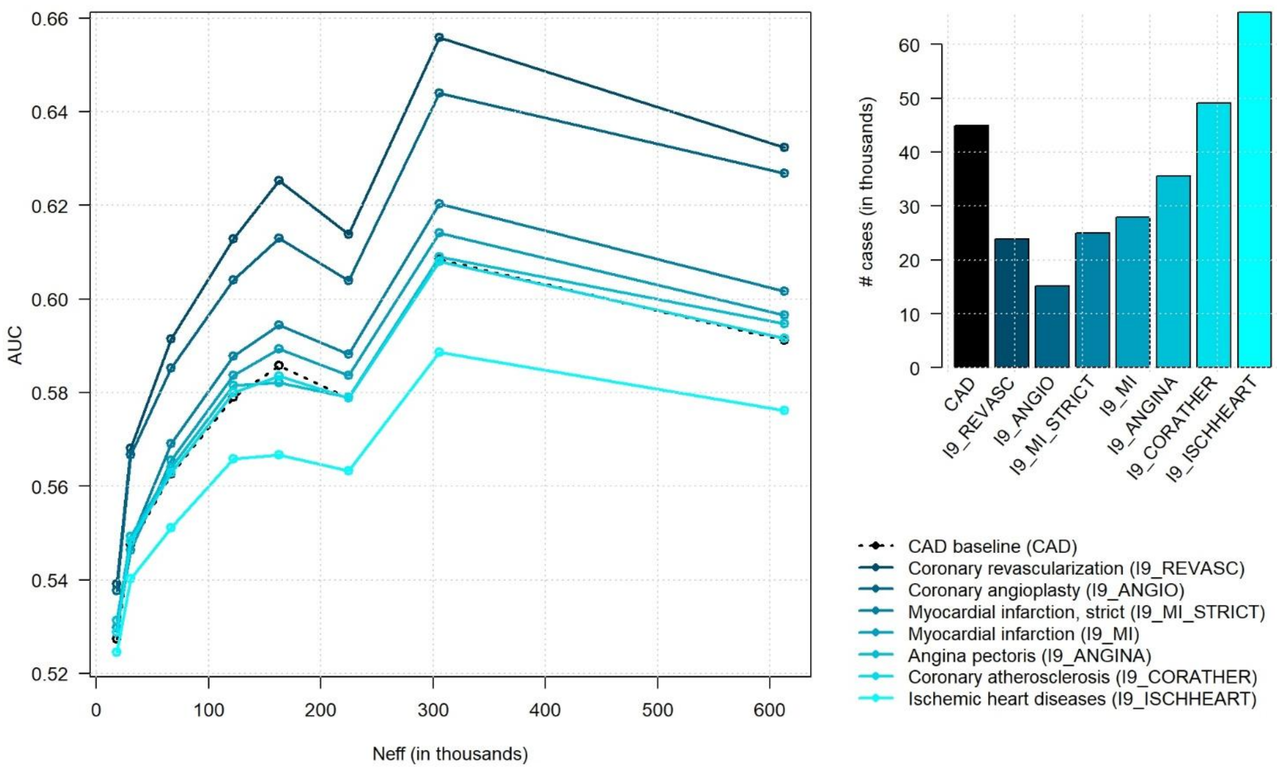

Figure S12. Baseline and attenuated alpha models and proportion of heritability captured

Panel a) shows the relationship between the minor allele frequency (MAF) and the per-allele effect relative to a base of 1 for a MAF of 0.5 as defined in the α model. Panel b) shows the same relationship for $\alpha = -0.3$ after applying the proposed *ad hoc* attenuated model and using various weights. Panel c) shows the heritability captured by imputed variants and derived based on r^2_{imput}, the squared-correlation between the sequenced variants and the imputed ones, as a function of the value of alpha. Panel d) shows the heritability captured by genotyped variants and derived based on ρ^2, the variance of sequenced variants captured by genotyped variants through linkage disequilibrium as a function of the value of alpha. For both estimations we assumed the genetic effect are distributed randomly on the genome based on the α model after applying the attenuation described in panel b.

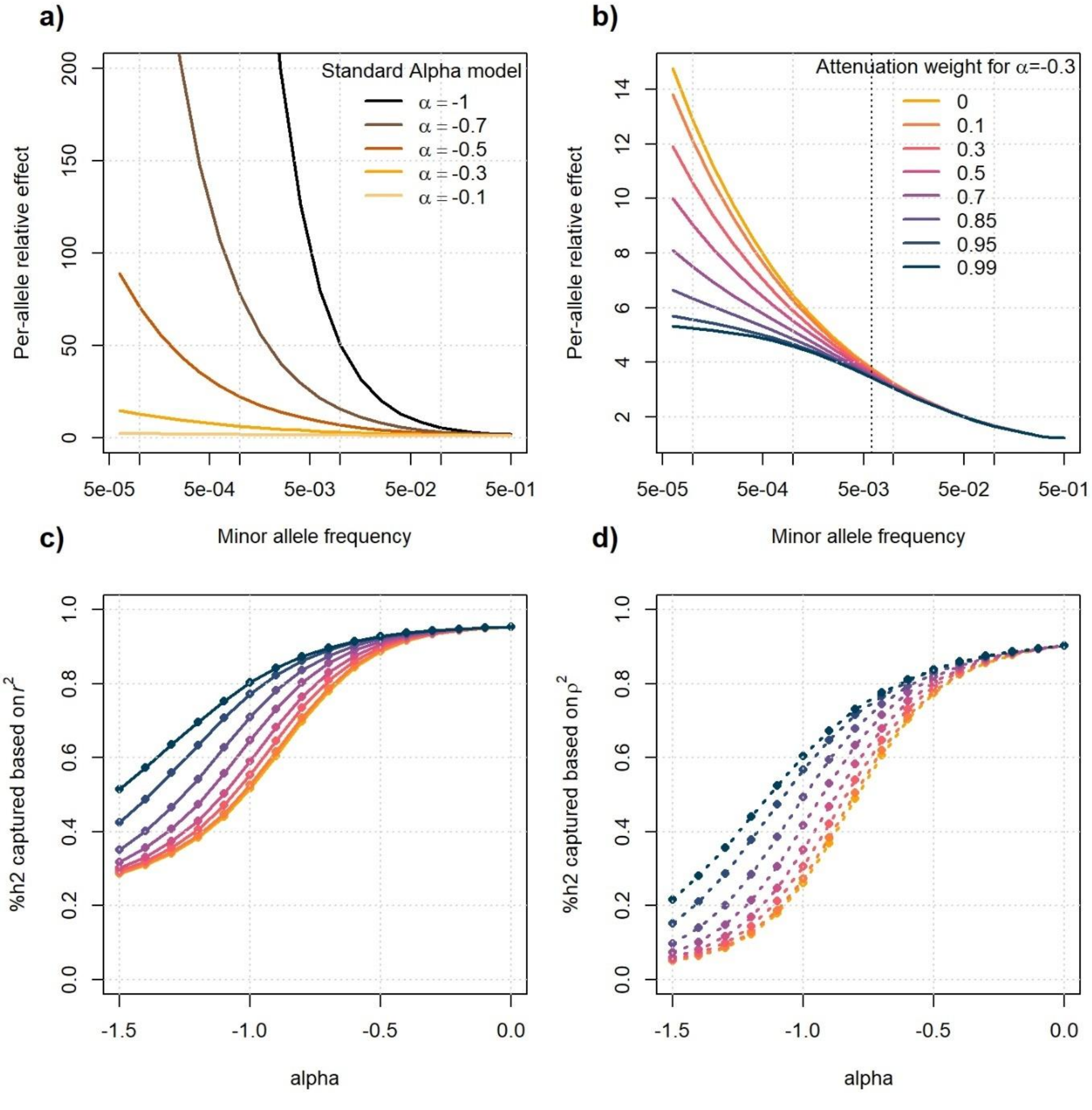

Figure S13. Variance of untyped variants captured by genotyped variants

We derived ρ_j^2 the variance of each sequenced variant j from the UK Biobank dataset captured by genotyped variants it its vicinity. We first conducted a pilot analysis used genetic data from chromosome 22 to determine the size of the windows around each variant j to be used for an optimal estimation of ρ_j^2. For this pilot, we predicted the best guess value from imputed genotypes (referred as the index variant) using genotyped variants as predictors while varying the size of the window in ±[100Kb, 3Mb] around the index. For each index we derived: i) the adjusted r-squared from a multiple regression derived in the entire sample; and ii) the squared-correlation between the predicted index and the true index using a train-test approach, were 66% (N=100K) of the sample was used as a train set and the remaining 33% (N=50K) as a test set. Panel a) shows the average ρ_j^2 over all variants of the two metrics for each windows size considered in the pilot study, highlighting an optimal at ±1.5Mb. This optimal window was used in the sequenced data to derive ρ^2. Panel b) compares the ρ^2 against r_{imput}^2, the squared-correlation between the sequenced variants and the imputed ones in the full chromosome 22 data. The density of the data points is highlighted by a gradient of colours.

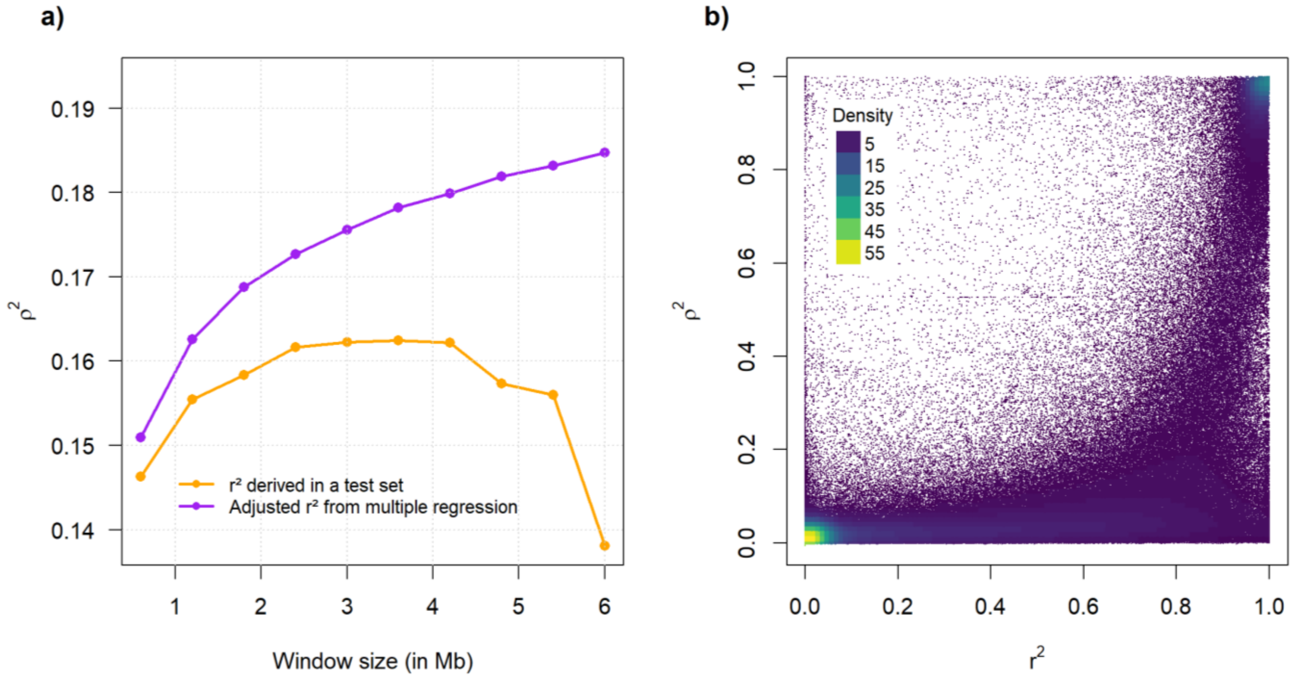

Figure S14. Variance explained by MAF bins

Expected variance explained across minor allele frequency (MAF) bins assuming the genetic effects are distributed following an α model, with α equals -1.3 (panel a)), -0.9 (panel b)), and -0.5 (panel c)). Simulations were conducted using MAF and estimated squared correlation between imputed and sequenced variants derived from 503,195 variants on chromosome 22 measured in 125,152 participants of European ancestry from the UK Biobank study. MAF was split into 20 percentiles bins. Upper panel shows the proportion of heritability per bin under the α model from the full set of 503,195 variants (grey bar), the percentage of heritability captured by the 12,968 genotyped variants only (red bars), and the percentage of heritability captured by the genotyped variants and the imputed variants (blue bars). Lower panels show the cumulative proportion of heritability.

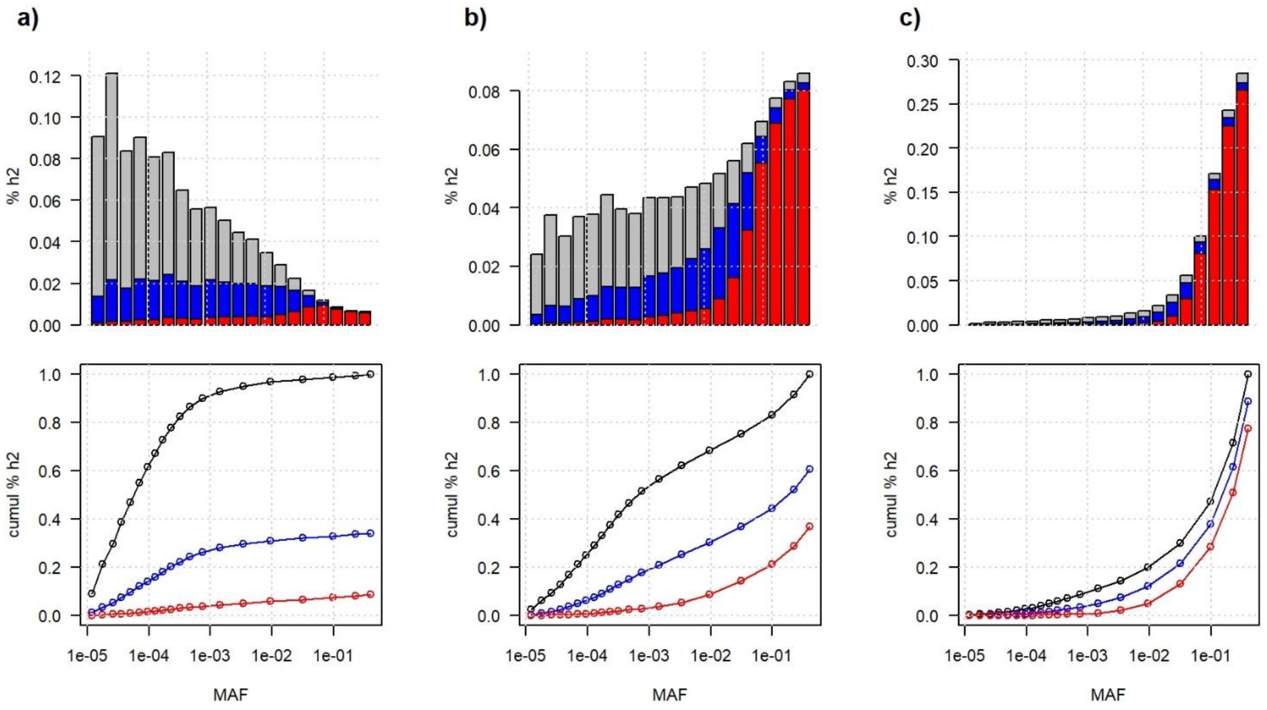

Figure S15. Estimation of alpha using UK Biobank individual-level data

Alpha was estimated using individual-level data from UK Biobank participants for the six outcomes: coronary artery disease (CAD), type 2 diabetes (T2D), breast cancer (BRCA), Alzheimer disease (AZ), asthma (AS), and obesity, but also for body mass index for comparison purposes. We considered three models: the standard alpha model (blue plots), the alpha model after applying an attenuation with a weight factor of $w = 0.6$ (red plots), and $w = 0.95$ (green plots), as described in the method section and Figure S9. The panels present the -log(likelihood) as a function of the alpha use to build the GRM (genetic relationship matrix). Panel a) shows the full data, and panel b) a zoom on the y-abscise to highlight the maximum after applying the R *loss()* smoothing function.

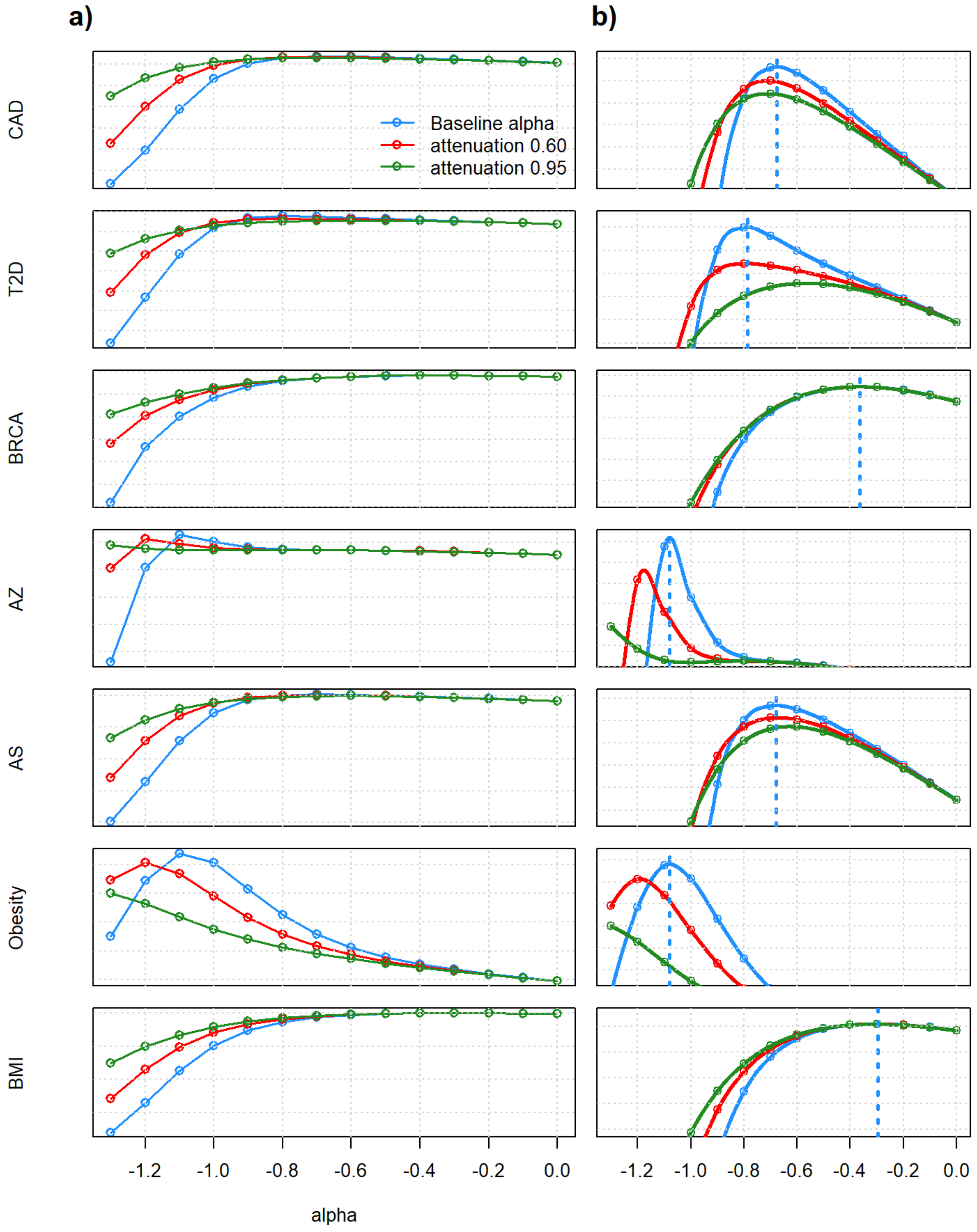

Figure S16. Validation of the GRM-GCTA pipeline using simulated data

Estimate of alpha in simulated data using restricted maximum likelihood method as implemented in GCTA. Using 10,000 UK Biobank participants and variants with MAF over 0.1%, we simulated phenotypes with heritability of 0.5, assuming 1% of the variants to be causal, and drawing effect using alpha values in the range [-1,0] with a step of 0.1. For each simulated phenotype, we computed the log-likelihood of 11 candidate alpha values in [-1,0] and retain the alpha displaying the maximum likelihood. Panel a) presents alpha estimates from models where causal variants were drawn from all variants with a MAF > 0.1%. Panel b) presents alpha estimated from models where causal variants were drawn from variants with a MAF in [0.1% and 1%] and using the corresponding genetic relationship matrix.

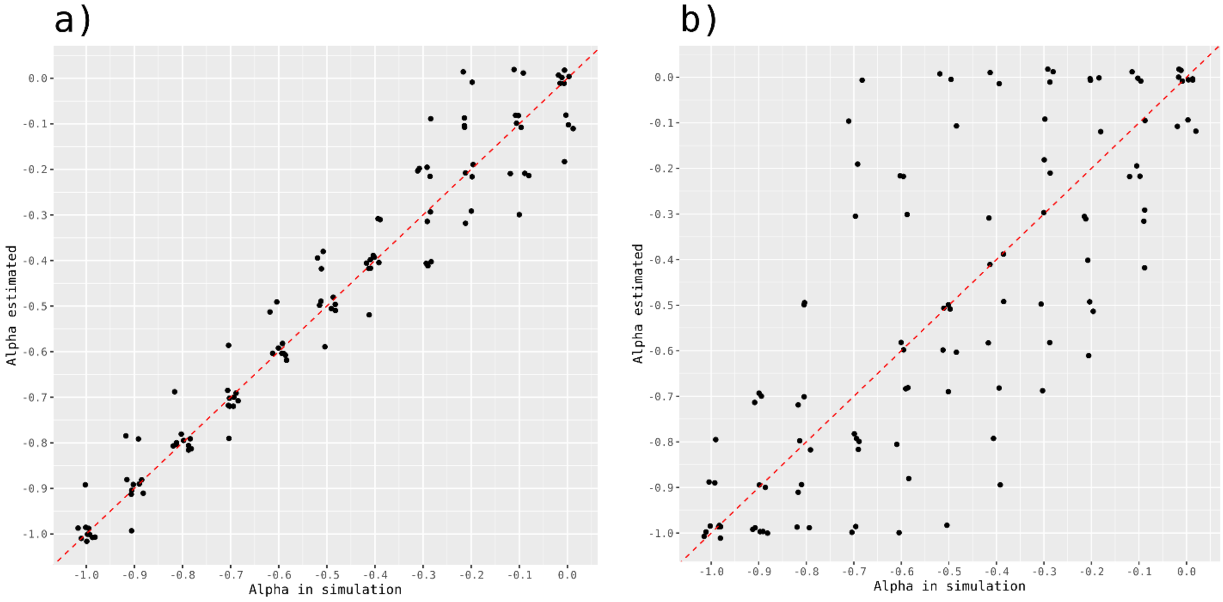

Figure S17. Impact of prevalence on estimated maximum achievable AUC

Estimates of the maximum achievable AUC for six outcomes: coronary artery disease, type 2 diabetes, breast cancer, Alzheimer disease, asthma, and obesity (using body mass index GWAS). Those AUCs were derived based on various estimates of heritability: twin studies (AUC_{Twin}), heritability derived using five competitive approaches: LDSC regression, sumHer, SBayesS, Genesis, and MiXer (AUC_{GWAS}) applied to the largest GWAS available for each disease, and twin study heritability captured by imputed variants ($AUC_{Imputed}$). For each disease, the four columns correspond to four prevalence estimates: US prevalence pulled from the CDC database (used in Figure 3c), and prevalence for US, the Uk and Finland extracted from other sources. Prevalence for asthma, CAD, T2D and Alzheimer disease were extracted from the Institute for Health Metrics and Evaluation (IHME) website (https://vizhub.healthdata.org/gbd-results/?params=gbd-api-2021-permalink/da654804ed46ce98fa58705f8713e236). Prevalence for breast cancer were extracted from Zheng et al 2023[27]. Prevalence for obesity were extracted from the World Health Organisation (WHO) website (https://apps.who.int/gho/data/node.main.A900A). The black dashed lines correspond to the most recent estimates of AUC in real data from the literature, derived based on approximately 1M hapmap3 variants.

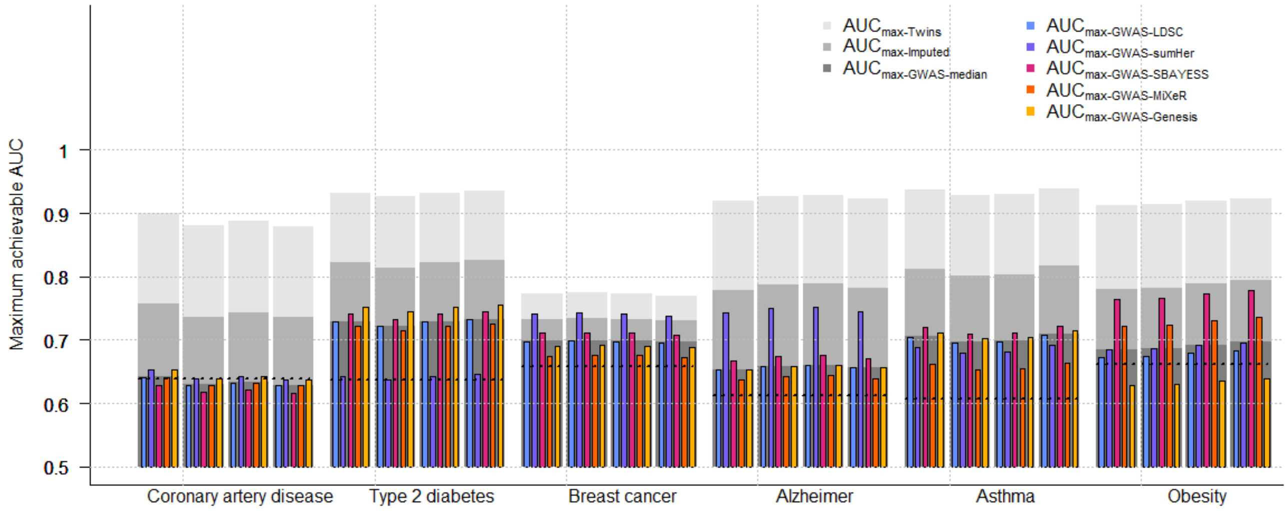

Figure S18. Correlation between functional annotations

Correlation between 1,099 functional annotations from nine categories: GENCODE, TFBS (transcription factor binding site), FANTOM5 (functional annotation of the mammalian genome version 5), promoters, enhancers, and dyadic from Roadmap, DHS (DNase I hypersensitive sites) derived from two studies[28], and super enhancer.

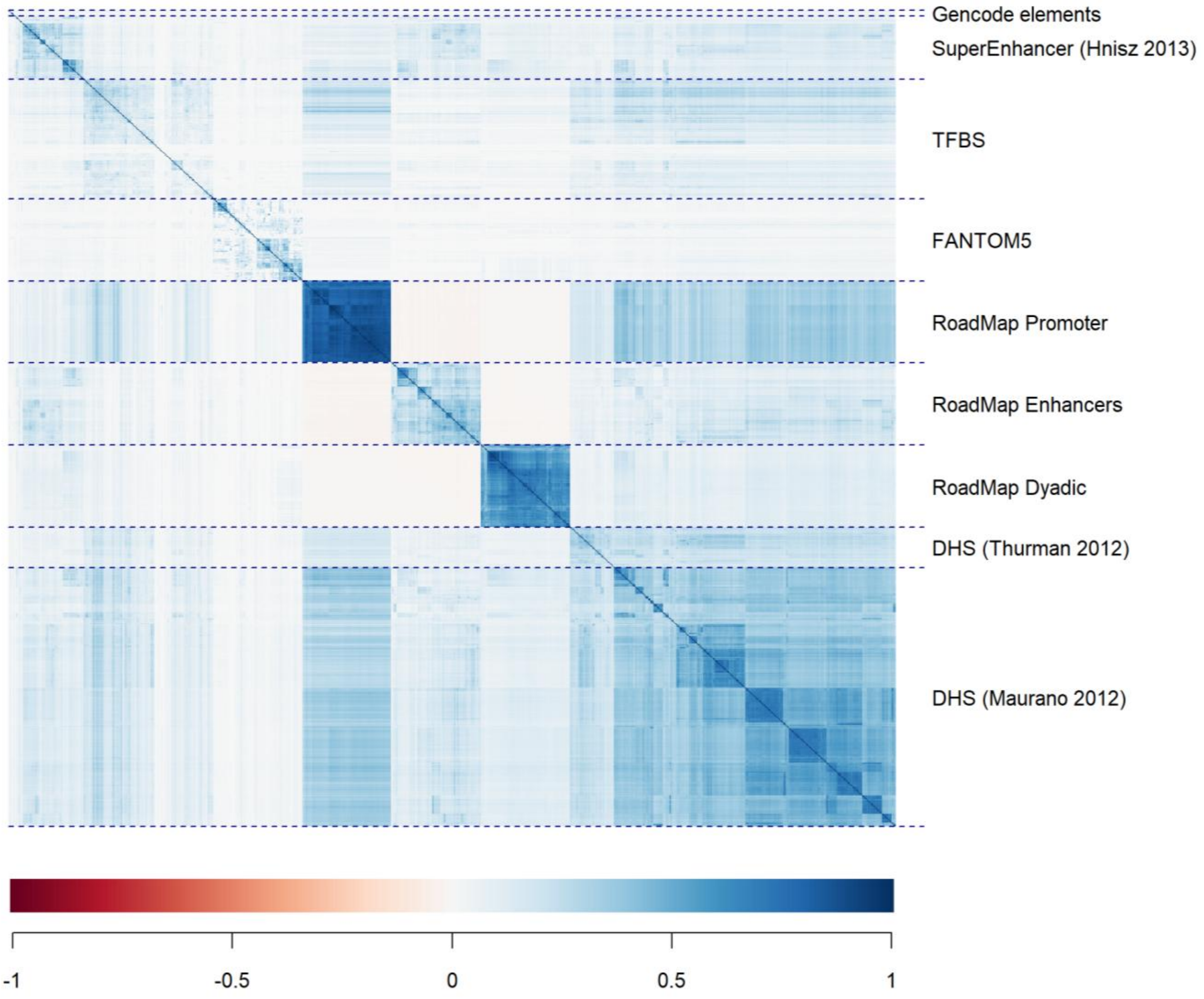

Figure S19. Distribution of imputation quality across GENCODE annotations

Panel a) shows the averaged quality of imputation measured as the squared-correlation (r^2_{imput}) derived between true and imputed genotypes in the UK Biobank for each of the seven GENCODE annotations: intron, gene, exon, CDS (coding DNA sequence), tss (transcription start site), tts (transcription termination site), and UTR (untranslated regions). Panel b) shows the proportion of heritability captured by imputed variants based on r^2_{imput} and assuming genetic effects are distributed following an alpha model. The relationship was derived assuming the causal variants are randomly distributed (red curve), or assuming the causal variants fall only into each of the seven annotations (green to black gradient).

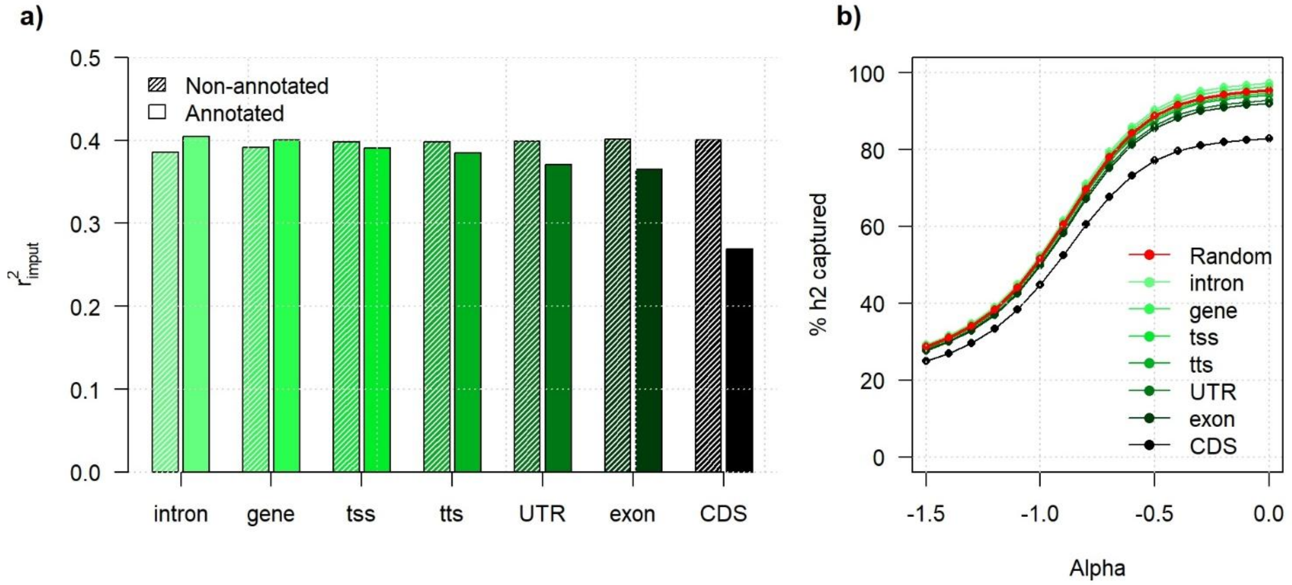

Figure S20. Top annotations associated with imputation quality

Relationship between top functional annotations and the quality of imputation measured as the squared-correlation (r^2_{imput}) between true and imputed genotypes. The barplot displays the change in the average r^2_{imput} between annotated and non-annotated variants for annotation reaching a significance p-value of 1 x10^{-8}. The category of each annotation is indicated by a color code: TFBS (transcription factor binding site), FANTOM5 (functional annotation of the mammalian genome version 5), promoters, enhancers, and dyadic from Roadmap, DHS (DNase I hypersensitive sites) derived from two studies, and super enhancer.

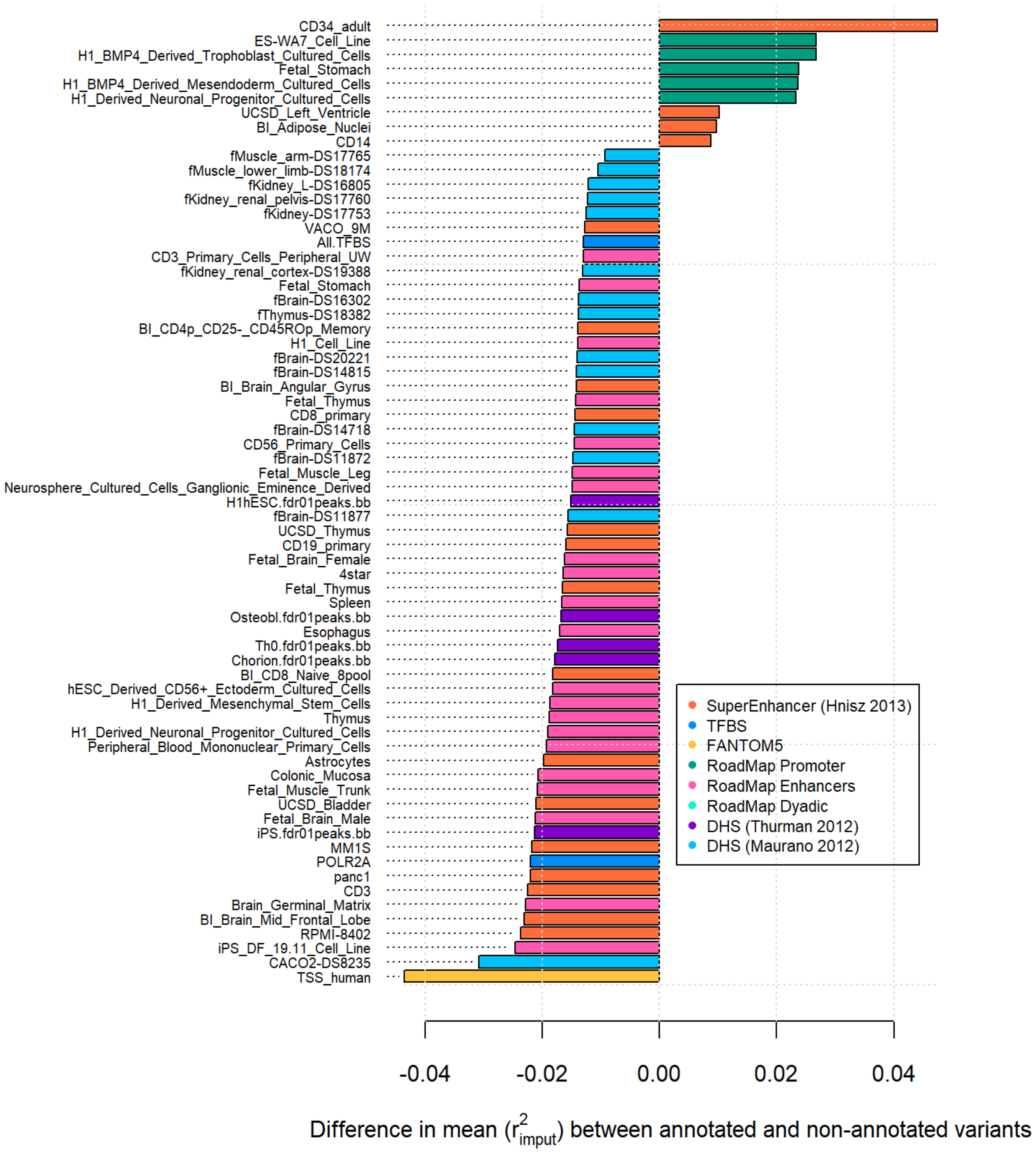

References

1. Prive, F., Luu, K., Blum, M.G.B., McGrath, J.J. & Vilhjalmsson, B.J. Efficient toolkit implementing best practices for principal component analysis of population genetic data. *Bioinformatics* **36**, 4449-4457 (2020).
2. Jansen, I.E. *et al.* Genome-wide meta-analysis identifies new loci and functional pathways influencing Alzheimer's disease risk. *Nat Genet* **51**, 404-413 (2019).
3. Lamnidis, T.C. *et al.* Ancient Fennoscandian genomes reveal origin and spread of Siberian ancestry in Europe. *Nat Commun* **9**, 5018 (2018).
4. Prive, F. *et al.* Portability of 245 polygenic scores when derived from the UK Biobank and applied to 9 ancestry groups from the same cohort. *Am J Hum Genet* **109**, 373 (2022).
5. Han, Y. *et al.* Genome-wide analysis highlights contribution of immune system pathways to the genetic architecture of asthma. *Nat Commun* **11**, 1776 (2020).
6. Mahajan, A. *et al.* Fine-mapping type 2 diabetes loci to single-variant resolution using high-density imputation and islet-specific epigenome maps. *Nat Genet* **50**, 1505-1513 (2018).
7. Zhang, H. *et al.* Genome-wide association study identifies 32 novel breast cancer susceptibility loci from overall and subtype-specific analyses. *Nat Genet* **52**, 572-581 (2020).
8. Yengo, L. *et al.* Meta-analysis of genome-wide association studies for height and body mass index in approximately 700000 individuals of European ancestry. *Hum Mol Genet* **27**, 3641-3649 (2018).
9. Genomes Project, C. *et al.* A global reference for human genetic variation. *Nature* **526**, 68-74 (2015).
10. Lee, S.H., Wray, N.R., Goddard, M.E. & Visscher, P.M. Estimating missing heritability for disease from genome-wide association studies. *Am J Hum Genet* **88**, 294-305 (2011).
11. Liu, C.C., Liu, C.C., Kanekiyo, T., Xu, H. & Bu, G. Apolipoprotein E and Alzheimer disease: risk, mechanisms and therapy. *Nat Rev Neurol* **9**, 106-18 (2013).
12. Prive, F., Arbel, J. & Vilhjalmsson, B.J. LDpred2: better, faster, stronger. *Bioinformatics* (2020).
13. Kunkle, B.W. *et al.* Genetic meta-analysis of diagnosed Alzheimer's disease identifies new risk loci and implicates Abeta, tau, immunity and lipid processing. *Nat Genet* **51**, 414-430 (2019).
14. Karlsson, I.K. *et al.* Measuring heritable contributions to Alzheimer's disease: polygenic risk score analysis with twins. *Brain Commun* **4**, fcab308 (2022).
15. Zhang, Y., Qi, G., Park, J.H. & Chatterjee, N. Estimation of complex effect-size distributions using summary-level statistics from genome-wide association studies across 32 complex traits. *Nat Genet* **50**, 1318-1326 (2018).
16. Zeng, J. *et al.* Widespread signatures of natural selection across human complex traits and functional genomic categories. *Nat Commun* **12**, 1164 (2021).
17. Speed, D. & Balding, D.J. SumHer better estimates the SNP heritability of complex traits from summary statistics. *Nat Genet* **51**, 277-284 (2019).
18. Bulik-Sullivan, B.K. *et al.* LD Score regression distinguishes confounding from polygenicity in genome-wide association studies. *Nat Genet* **47**, 291-5 (2015).
19. Holland, D. *et al.* Beyond SNP heritability: Polygenicity and discoverability of phenotypes estimated with a univariate Gaussian mixture model. *PLoS Genet* **16**, e1008612 (2020).
20. Frei, O. *et al.* Bivariate causal mixture model quantifies polygenic overlap between complex traits beyond genetic correlation. *Nat Commun* **10**, 2417 (2019).
21. International HapMap, C. *et al.* Integrating common and rare genetic variation in diverse human populations. *Nature* **467**, 52-8 (2010).
22. Devlin, B. & Roeder, K. Genomic control for association studies. *Biometrics* **55**, 997-1004 (1999).
23. Speed, D., Hemani, G., Johnson, M.R. & Balding, D.J. Improved heritability estimation from genome-wide SNPs. *Am J Hum Genet* **91**, 1011-21 (2012).
24. Schoech, A.P. *et al.* Quantification of frequency-dependent genetic architectures in 25 UK Biobank traits reveals action of negative selection. *Nat Commun* **10**, 790 (2019).
25. Yang, J., Lee, S.H., Goddard, M.E. & Visscher, P.M. GCTA: a tool for genome-wide complex trait analysis. *Am J Hum Genet* **88**, 76-82 (2011).
26. Chang, C.C. *et al.* Second-generation PLINK: rising to the challenge of larger and richer datasets. *Gigascience* **4**, 7 (2015).

27. Zheng, R. *et al.* Global, regional, and national lifetime probabilities of developing cancer in 2020. *Sci Bull (Beijing)* **68**, 2620-2628 (2023).
28. Thurman, R.E. *et al.* The accessible chromatin landscape of the human genome. *Nature* **489**, 75-82 (2012).
