# Supplementary material for: Polygenic risk score prediction accuracy convergence
Source: HGG Adv. 2025 May 14;6(3):100457. doi: 10.1016/j.xhgg.2025.100457 (PMC12167061; doi:10.1016/j.xhgg.2025.100457)
Supplement: Document S2. Article plus supplemental information [file mmc3.pdf]

HGG
Advances

ARTICLE

Polygenic risk score prediction accuracy convergence

Léo Henches,[1] Jihye Kim,[2] Zhiyu Yang,[3] Simone Rubinacci,[3] Gabriel Pires,[1] Clara Albiñana,[4] Christophe Boetto,[1] Hanna Julienne,[1] Arthur Frouin,[1] Antoine Auvergne,[1] Yuka Suzuki,[1] Sarah Djebali,[5] Olivier Delaneau,[6] Andrea Ganna,[3] Bjarni Vilhjálmsson,[4,7] Florian Privé,[4] and Hugues Aschard[1,2,8,*]

Summary

Polygenic risk scores (PRSs) models trained from genome-wide association study (GWAS) results are set to play a pivotal role in biomedical research addressing multifactorial human diseases. The prospect of using these risk scores in clinical care and public health is generating both enthusiasm and controversy, with varying opinions among experts about their strengths and limitations. The performance of existing polygenic scores is still limited but is expected to improve with increasing GWAS sample sizes and the development of new, more powerful methods. Theoretically, the variance explained by PRS can be as high as the total additive genetic variance, but it is unclear how much of that variance has already been captured by PRS. Here, we conducted a retrospective analysis to assess progress in PRS prediction accuracy since the publication of the first large-scale GWASs, using data from six common human diseases with sufficient GWAS information. We show that although PRS accuracy has grown rapidly over the years, the pace of improvement from recent GWAS has decreased substantially, suggesting that merely increasing GWAS sample sizes may lead to only modest improvements in risk discrimination. We next investigated the factors influencing the maximum achievable prediction using whole-genome sequencing data from 125,000 UK Biobank participants and state-of-the-art modeling of polygenic outcomes. Our analyses suggest that increasing the variant coverage of PRS, using either more imputed variants or sequencing data, is a key component for future improvements in prediction accuracy.

Introduction

Most common human diseases exhibit strong polygenic inheritance, characterized by a very large number of genetic variants with small effects. This scattered distribution of risk has severely hampered the initial goal of using genetic association studies for personalized medicine through individualized disease risk predictions, prevention strategies, and treatments.[1–8] This issue was recognized early in the genome-wide association study (GWAS) era, and the community developed a strong case for genetic risk profiling based on polygenic risk scores (PRSs) derived from GWAS results.[9] In its simplest form, a PRS for an individual is the summation of multiple single-nucleotide polymorphisms (SNPs) weighted by their effect sizes estimated from independent GWAS data. Initially, PRSs were constructed from a small number of independent genome-wide significant variants, but they have evolved to include thousands to millions of variants selected from full GWAS results using optimized selection criteria and weighting schemes.[10,11] As with the prediction of any highly multifactorial outcome, PRS accuracy largely depends on the sample size of the dataset used to estimate individual predictor effects. GWAS sample sizes—and therefore the predictive performance of PRSs—have increased substantially over time. However, it remains unclear how much predictive accuracy has already been achieved for common human diseases and how much further improvement can be attained through future, larger GWAS.

Characterizing the PRS accuracy-sample size relationship in real data is challenging for two reasons. First, this relationship is expected to vary across disease parameters, including prevalence, heritability,[12–14] and the distribution of effects at causal variants,[15,16] which can be difficult to estimate in practice. Second, there is notable heterogeneity in the implementation of PRSs.[17–19] Existing studies have used a variety of methods, models, data sources, and populations, including multi-ancestry[20–24] and multi-phenotype approaches.[25–27] This heterogeneity limits our ability to determine the key drivers of PRS improvement. A formal evaluation of PRS performance as a function of sample size requires GWASs conducted using populations of similar genetic ancestry, similar statistical tests, comparable numbers of input variants, and consistent modeling (e.g., including the same covariates). Furthermore, the PRS must be derived using the same approach and applied to a single test dataset that is not included in the GWAS used to build the PRS to avoid overfitting. This point is particularly challenging, as most existing disease GWASs

[1]Institut Pasteur, Université de Paris, Department of Computational Biology, 75015 Paris, France; [2]Department of Epidemiology, Harvard T.H. Chan School of Public Health, Boston, MA, USA; [3]Institute for Molecular Medicine Finland (FIMM), University of Helsinki, Helsinki, Finland; [4]National Centre for Register-Based Research, Aarhus University, 8210 Aarhus, Denmark; [5]IRSD, Université de Toulouse, INSERM, INRAE, ENVT, University Toulouse III - Paul Sabatier (UPS), Toulouse, France; [6]Department of Computational Biology, University of Lausanne, Lausanne, Switzerland; [7]Bioinformatics Research Centre, Aarhus University, 8000 Aarhus, Denmark
[8]Lead contact
*Correspondence: hugues.aschard@pasteur.fr
https://doi.org/10.1016/j.xhgg.2025.100457.
© 2025 The Authors. Published by Elsevier Inc. on behalf of American Society of Human Genetics.
This is an open access article under the CC BY license (http://creativecommons.org/licenses/by/4.0/).

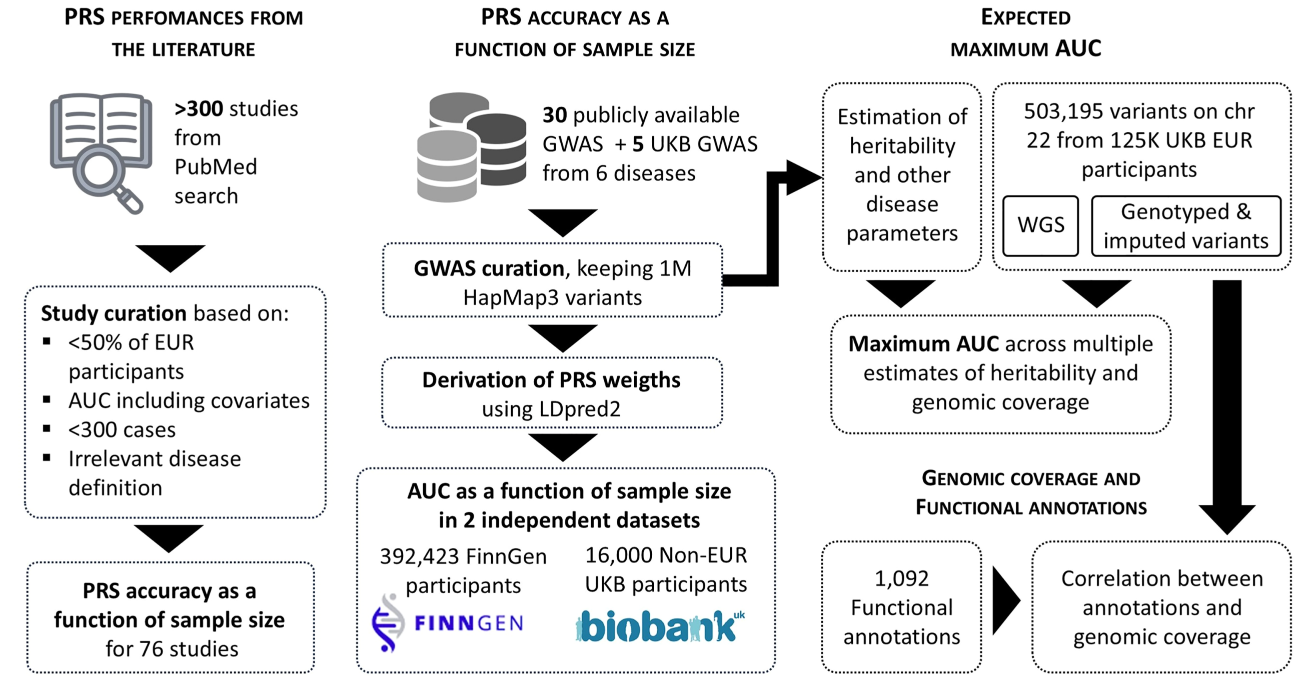

Figure 1. Study flowchart
The primary analyses conducted in the study for the six diseases included assessing the accuracy of polygenic risk scores (PRSs) using the area under the receiver operating characteristic curve (AUC) as reported in the literature; estimating PRS performance using genome-wide association study (GWAS) summary statistics and a harmonized pipeline; evaluating the maximum AUC based on estimated disease parameters and UKB sequencing data; and analyzing the enrichment between genomic coverage and functional annotations.

are meta-analyses that include all available data. These constraints substantially limit the number of GWAS datasets that can be used. After an extensive literature review, we identified six diseases for which sufficient data meeting these criteria were available: coronary artery disease (CAD), breast cancer, type 2 diabetes, Alzheimer disease (AD), asthma, and obesity. We conducted a retrospective study to examine how PRS prediction accuracy for these six diseases has evolved with increasing GWAS sample sizes over the past 15 years. This evaluation was carried out using two independent datasets: the FinnGen cohort and non-European ancestry participants from the UK Biobank (UKB). We further investigated how existing models, state-of-the-art sequencing data, and functional annotation data may inform potential future improvements. An overview of the study design is presented in Figure 1.

Material and methods

GWAS data assembly

We collected publicly available GWAS summary statistics for six outcomes: type 2 diabetes, coronary heart disease, breast cancer, AD, asthma, and body mass index (BMI), for which we used to study obesity. The design of each study was carefully assessed to ensure it met stringent inclusion criteria for our analysis: (1) all studies had to include a majority of individuals of European ancestry; (2) studies with limited genetic coverage, such as exome-wide screening or genotyping chips without imputation (e.g., MetaboChip, ImmunoChip), were excluded; and (3) phenotype definitions had to be relatively homogeneous, although we retained some studies with heterogeneity for comparative purposes (e.g., early-onset asthma, AD proxy defined from parental status). In some meta-analyses, we also accessed GWAS results from individual cohorts, which were used as additional data points. After quality control filtering, 30 GWASs summary statistics with the required baseline data (coded allele, signed statistics, and p value) remained for analysis. We completed our panel by conducting five additional GWASs of modest sample size in the UKB using unrelated participants of European ancestry, with cases sampled from the entire cohort for four outcomes: breast cancer, CAD, asthma, and BMI. All 35 GWAS were harmonized and converted to hg38 using the *liftover* package.[28] As one of the primary goals was to compare the predictive accuracy of PRS scores in the FinnGen cohort, we retained only variants available in that dataset. Unless otherwise specified, we defined the effective sample size of binary GWAS outcomes as $N_{eff} = 4/[1/N_{case} + 1/N_{controls}]$, which re-scales cohorts with unequal case-control numbers to a common unit.[29] The list of GWAS used is presented in Table 1.

Estimation of PRS prediction performances in real data

For each GWAS, we derived a vector of variant-specific PRS weights $\boldsymbol{\gamma} = (\gamma_1, \gamma_2 \ldots \gamma_M)$, where γ_i is the weight for variant i and M is the number of variants available in the GWAS. We used the LDpred2 approach[11] with the "auto" option, which automatically estimates hyperparameters, including PRS sparsity and SNP heritability. We employed 30 Gibbs sampling chains. Because the GWASs included predominantly individuals of European ancestry and in-sample linkage disequilibrium (LD) was not

Table 1. GWASs used to derive polygenic risk scores

Phenotype	Reference	N_{case}	N_{cont}	N_{eff}	No. of variants[a]
CAD	UK Biobank (5K)	5,000	50,000	18,182	1,041,959
	C4D et al.[30]	15,420	15,062	30,478	540,233
	Schunkert et al.[31]	22,233	64,762	66,204	2,420,308
	Nikpay et al.[32]	60,801	123,504	162,973	9,455,779
	Nelson et al.[33]	71,602	260,875	224,727	9,020,475
	Harst et al.[34]	34,541	261,984	122,070	7,947,838
	Harst et al.[34]	122,733	424,528	380,832	7,947,838
	Aragam et al.[23]	181,522	984,168	613,021	20,073,070
Type 2 diabetes	Cai et al.[35]	9,978	13,348	22,839	8,924,493
	Morris et al.[36]	12,171	56,862	40,101	2,473,442
	Scott et al.[37]	26,676	132,532	88,825	12,056,347
	Xue et al.[38]	62,892	596,424	227,571	5,053,016
	Mahajan et al.[39]	74,124	824,006	272,026	23,465,133
Breast cancer	UK Biobank (5K)	5,000	50,000	18,182	1,041,959
	Michailidou et al.[40] (GWAS meta-analysis)	14,910	17,588	32,277	11,792,543
	Michailidou et al.[41](iCOGS GWAS)	46,785	42,892	89,508	11,792,543
	Michailidou et al.[42] (OncoArrray GWAS)	61,282	45,494	104,442	11,792,543
	Zhang et al.[43]	133,384	113,789	245,620	11,792,543
Alzheimer disease	Li et al.[44]	753	736	1,489	391,067
	Lambert et al.[45]	17,008	37,154	46,669	7,053,170
	Kunkle et al.[46]	21,982	41,944	57,693	11,480,633
	Jansen et al.[47]	71,880	383,378	242,124	13,367,300
Asthma	UK Biobank (1K)	1,000	10,000	3,636	1,041,959
	Shrine et al.[48]	5,135	25,675	17,117	33,771,859
	Moffatt et al.[49]	10,365	16,110	25,228	567,590
	Zhu et al.[50]	14,085	76,768	47,606	7,488,536
	Demenais et al.[51]	23,948	118,538	79,692	2,001,257
	Han et al.[52]	64,538	329,321	215,851	9,572,557
BMI	UK Biobank (20K)	20,000		20,000	1,041,959
	UK Biobank (80K)	80,000		80,000	1,041,959
	Speliotes et al.[53]	125,865		125,865	2,471,517
	Locke et al.[54]	322,206		322,206	2,554,638
	Yengo et al.[55]	795,640		795,640	2,336,270
	Elsworth et al.[56]	454,884		454,884	9,851,867

[a]Total number of variants available in the original GWAS.

available, we used European descent participants from the UKB as the reference panel for LD derivation. The use of external LD data matched to the GWAS ancestry is expected to have a negligible impact on PRS accuracy.[57,58] We restricted the analysis to GWAS variants overlapping with 1,054,330 HapMap3 variants.[59] This restriction was due to the computational cost and memory demands of LDpred2 (also existing for other PRS software), which scale quadratically with the number of variants. For each GWAS, we also estimated per-variant effective sample sizes (N_{eff_SNP}) and filtered out variants with values lower than 50% or higher than 110% of the expected maximum to avoid miscalibration of regression coefficients.[57,60] We then constructed PRSs in independent test datasets using the derived weights. The score for an individual was computed as $PRS = \boldsymbol{\gamma}^t\mathbf{X}$, where $\mathbf{X}$ is a genotype matrix with alleles coded to match the original GWAS. Because most European ancestry cohorts were used in the original GWASs, we

focused our application on evaluating relative performance and trends in area under the receiver operating characteristic curve (AUC) using two datasets.

The first dataset included 16,000 unrelated UKB participants of non-European ancestry, categorized into 6 groups (Ashkenazi, Iranian, Indian, Chinese, Caribbean, and Nigerian) as defined by Privé et al.[61] These data were also used for PRS fine-tuning and averaging weights across Gibbs sampling chains. The second dataset comprised 392,423 Finnish ancestry participants from the FinnGen cohort (see supplementary methods). Although genetic differences between training and test populations reduce absolute prediction performance, PRS portability across populations is expected to be approximately linear. Therefore, GWAS sample size vs. PRS performance trends should remain informative. For instance, the top GWAS hits between FinnGen and European samples showed high concordance (mean squared correlation of effect estimates = 0.56; Figure S1).

We evaluated PRS performance using the AUC. In the non-European UKB samples, AUCs were first computed separately for each ancestry group. Due to small case numbers per group, we meta-analyzed the results across groups using standard inverse-variance meta-analysis: $AUC_{combined} = \sum_{i=1...5}[AUC_i/\sigma_i^2]/\sum_{i=1...5}[1/\sigma_i^2]$, and $SE(AUC_{combined}) = 1/\sum_{i=1...5}[1/\sigma_i^2]$, where AUC_i and σ_i^2 represent the AUC and variance for population $i = 1...5$. Importantly, no additional conventional risk factors (e.g., clinical, demographic, environmental) were included in the models, so the reported AUCs reflect the marginal predictive power of polygenic models alone.

Variance captured by genotyped and imputed variants

We examined the variance captured by genotyped and imputed variants using real genetic data and simulated genetic effects. Consider a standardized phenotype Y drawn from a polygenic model and defined as the linear additive effect of M standardized causal variants. Its total variance equals $V_y = h^2 + V_e$, where h^2 is the genetic variance (heritability) and V_e is the residual environmental variance. Assuming that genetic effects at causal variants are independent of variant correlations, heritability can be approximated as $h^2 = \sum_M \beta_i^2$, where β_i is the effect of a standardized variant i. We estimated the proportion of h^2 that could be recovered under an infinite GWAS sample size when only a subset S of variants has been genotyped, while the remaining $M \notin S$s are either imputed or missing. Two key metrics were considered: h_I^2, the additive genetic variance captured by all M variants, both genotyped and imputed using advanced methods,[62] and h_G^2, the genetic variance captured using only the subset S of genotyped variants but accounting for the contribution of the $M \notin S$ untyped variants through LD. The first metric is defined as $h_I^2 = \sum_M \beta_i^2 r_{imput}^2$, where r_{imput}^2 is the squared correlation between the sequenced variant i and its imputed value. The second metric is defined as $h_G^2 = \sum_S \beta_i^2 + \sum_{M \notin S} \beta_j^2 \rho_j^2$, where ρ_j^2 represents the squared correlation between the untyped variants j and the set of genotyped variants. In theory, h_G^2 should closely approximate GWAS-based heritability (h_{GWAS}^2) as estimated by existing software.[63] The former (h_I^2) is expected to capture additional variance from untyped variants poorly tagged by the genotyped ones.

Both metrics were estimated using a subset of 125,152 UKB participants of European ancestry with both genome-wide genotyping and whole-genome sequencing data. For h_I^2, we used imputed SNP-array data from the UKB. Imputed variants were lifted over to GRCh38, retaining 99.5% of sites. We then computed r_{imput}^2 between each sequenced variant and its imputed dosage using standard univariate linear regression. For h_G^2, we used genotyped variants available on the UKB Axiom array[64] as the baseline. The ρ^2 values were computed using adjusted squared correlation obtained from standard multiple linear regressions, where each non-genotyped variant was predicted from a set Ω of nearby genotyped variants within a ± 1.5-Mb window. For simplicity, both r_{imput}^2 and ρ^2 were calculated using only chromosome 22, assuming that it is representative of the entire genome. This chromosome includes 12,968 genotyped variants. Of the 659,092 sequenced variants, 503,195 remained after filtering out those with minor allele frequency (MAF) < 0.001%. As expected, the two metrics were highly correlated ($cor(\rho^2, r_{imput}^2) = 0.71$), but the average r_{imput}^2 was substantially higher than ρ^2 ($\overline{r_{imput}^2} = 0.50$, $\overline{\rho^2} = 0.25$).

In our simulations, the proportion of heritability captured was derived using both r_{imput}^2 and ρ^2 and effect size $\beta = (\beta_1 ... \beta_M)$ drawn from a normal distribution under the alpha model, where the expected effect of a variant is proportional to its variance raised to a power α.[65–67] This model posits that rare variants have larger per-allele effects than common variants when $\alpha < 0$. The genetic effect for variant i was drawn from $\beta_i | p_i \sim \mathcal{N}(0, \sigma_{g,\alpha}^2 \cdot [2p_i(1-p_i)]^{\alpha})$, where p_i is the MAF and $\sigma_{g,\alpha}^2$ is scaled to match a predefined heritability. In our analysis, we considered α values ranging from -1.5 to 0 and sampled $\sigma_{g,\alpha}^2$ uniformly over [0, 1]. Additionally, we explored an attenuated alpha model that reduces the influence of rare variants using an ad hoc iterative weighting function (see supplementary methods).

Maximum achievable AUC

The expected maximum achievable AUC from a polygenic model is determined primarily by the proportion of genetic variance captured by the variants used to construct the PRS. This maximum can be derived using the approximation proposed by Wray et al.[14]: $AUC_{max} \approx \Phi(((i - v)h^2)/\sqrt{h^2[(1-h^2 i(i-T)) + (1-h^2 v(v-T))]})$, where h^2 is the heritability on the liability scale, Φ is the cumulative density function of the normal distribution, z is the height of the standard normal density at the threshold $T = \Phi^{-1}(1-K)$ and with $i = z/K$ and $v = -z/(1-K)$, where K is the disease prevalence. We confirmed the validity of this approximation through simulation models involving independent causal variants with linear additive effect h^2 values in [0.2, 0.7] and prevalence K in [0.01, 0.25] (supplementary methods). Conditional estimates of AUC were derived by substituting h^2 with either h_G^2 or h_I^2, themselves derived based on alpha drawn in [−1.5, 0] and $\beta = (\beta_1 ... \beta_M)$ coefficients sampled from a normal distribution. For real-data analysis, disease prevalence values were sourced from the Centers for Disease Control and Prevention (CDC) website. We derived three estimates of heritability on the liability scale: (1) the total heritability derived from twins studies for CAD (0.55),[68] type 2 diabetes (0.72),[69] breast cancer (0.27),[70] AD after excluding the effect of *APOE* (0.49),[71,72] asthma (0.70),[73] and BMI (0.75)[74]; (2) h_{GWAS}^2, the heritability captured by GWAS variants and derived using five alternative approaches: SBayesS,[75] sumHer,[76] LD score regression (LDSC) regression,[77] GENESIS,[15] and MiXeR[78] (supplementary methods); and (3) h_I^2, the heritability captured by genotyped and imputed variants, which requires an estimate of the total heritability, the proportion of heritability captured by genotyped and imputed variants given α, and a value of α.

For the total heritability, we used the twins studies estimates. For the proportion of heritability captured, we used r^2_{imput}, the estimate derived using the UKB sequencing data. The choice of α was more challenging, and we ultimately used an *ad hoc* approach. Further details are provided below.

Most real-data estimates of h^2_{GWAS} are derived from genotyped variants and a modest subset of imputed variants filtered for high quality (typical info score $\geq$0.8). As a result, assuming our estimation of the heritability captured by genotyped variants is valid, the previously described h^2_G is expected to approximate h^2_{GWAS} if provided a relevant value of α. We assessed this equality using α derived from various approaches (SBayesS, sumHer, and individual-level data from the UKB), but we found large discrepancies. Ultimately, we adopted an *ad hoc* "best-fit" α approach: for each disease, we selected α so that h^2_G equals the median of the five GWAS-based heritability estimates. This α was then used to derive h^2_I and compute AUC predictions accordingly.

Genetic coverage and functional annotations

We evaluated the association between a range of functional annotations and the imputation quality (r^2_{imput}, the squared correlation between sequenced and imputed genotypes in the UKB). We used a total of 1,099 functional annotations pulled from mutiple sources: baseline GENCODE annotations, including intron, gene, exon, coding DNA sequence (CDS), transcription start site (TSS), transcription termination site (TTS), and untranslated regions (UTRs); epigenetic features across tissues and cell types: transcription factor binding sites (TFBSs) and functional annotation of the mammalian genome version 5 (FANTOM5); and regulatory elements: promoters, enhancers, dyadic annotations from Roadmap, DNase I hypersensitive sites (DHSs) from two sources, and super-enhancers. The analysis was performed on the same dataset used to derive h^2_I and h^2_G (503,195 sequenced chromosome 22 variants from 125,000 UKB participants of European ancestry). Associations between each functional annotation A_i and imputation quality were estimated using a standard univariate linear model: $r^2_{imput} \sim \delta_i A_i$. A sensitivity analysis was also conducted using a model adjusted for GENCODE annotations, $r^2_{imput} \sim \delta_i A_i + \sum_{k \in GENCODE} \delta_k A_k$, where A_k denotes GENCODE category indicators.

Results

PRS prediction accuracy and sample size

To illustrate the challenge of characterizing the sample size-PRS accuracy relationship in real data, we reviewed the literature and curated previous reports of genetic risk score prediction accuracy, expressed as the AUC, for CAD, breast cancer, type 2 diabetes, AD, asthma, and obesity (Table S1; supplementary methods). The studies spanned from 2006 to 2023 and the effective sample size (N_{eff}) ranged from 981 to 453,912. The reported predictive power showed a very modest linear trend with sample size and was instead characterized by substantial heterogeneity (Figure 2A). AUC increases were nominally significant for breast cancer ($\beta = 2.5 \times 10^{-4}$/1,000 increase in sample size, $p = 0.0096$) and obesity ($\beta = 3.1 \times 10^{-4}$, $p = 0.0038$), but were not significant for the other outcomes. For example, there was no clear trend for CAD ($\beta = -1.8 \times 10^{-5}$, $p = 0.90$) despite an effective sample size ranging from $N_{eff} = 4{,}522$ to $N_{eff} = 184{,}305$. This is likely due to several factors already discussed in the literature and complex to disentangle, including heterogeneity in the population characteristics (age, sex, fine-scale genetic ancestry within European population), disease definition, and the method used to derive the PRS weights.[17,19]

Prediction accuracy was then assessed using a harmonized pipeline, in which PRSs were derived from 35 curated GWASs and applied to two independent cohorts: FinnGen and non-European UKB participants. It shows a clear, non-linear increasing trend as a function of sample size, starting with a sharp rise for the first few studies, followed by a gradual decline in improvement (Figures 2B and S2; Tables S2–S4). The patterns are highly concordant across the two test datasets, displaying only the expected offset due to genetic ancestry differences between the training and test data.[61,79] The flattening of AUC improvement is especially striking for type 2 diabetes, obesity, breast cancer, and CAD. Asthma displays a noisier trend, potentially reflecting challenges in disease definition and diagnosis. AD shows a continuous increase in AUC for the non-European UKB samples, but a negligible increase for the most recent and largest GWAS in FinnGen, despite a 5-fold increase in effective sample size. This may be explained by the specificity of the AD PRS (Figure S3) and, in particular, the use of a proxy for disease status—a score based on the AD status of participants' parents—in this GWAS, which could introduce variability in heritability estimates of AD[80] and likely affect disease risk prediction.

Sensitivity analyses yield comparable results. Using alternative approaches to derive the PRS from the same curated GWAS data produces qualitatively similar prediction accuracy, but with higher heterogeneity (Figure S4; Table S5). Trends in prediction accuracy, measured by the coefficient of determination (R^2) and the odds ratios of the disease comparing the top 5% and top 1% PRS strata to the remaining population, are fully consistent with those observed for the AUC (Figure S5; Table S5), with additional variability only in the top 1% PRS, likely due to the modest sample size when using this stringent threshold. We also confirm the relevance of our stringent GWAS curation, in which we removed all GWASs displaying potential overlap with our testing samples. As shown in the experiment from Figure S6, even a small overlap between the training and test samples can artificially inflate prediction accuracy.

Modeling and robustness of AUC convergence

The convergence of prediction accuracy toward its maximum can be demonstrated using simple theoretical models[14] (Figure S7). Predicting the convergence rate in real data is considerably more challenging and requires estimating multiple parameters of the disease genetic model, including heritability, polygenicity, the distribution of

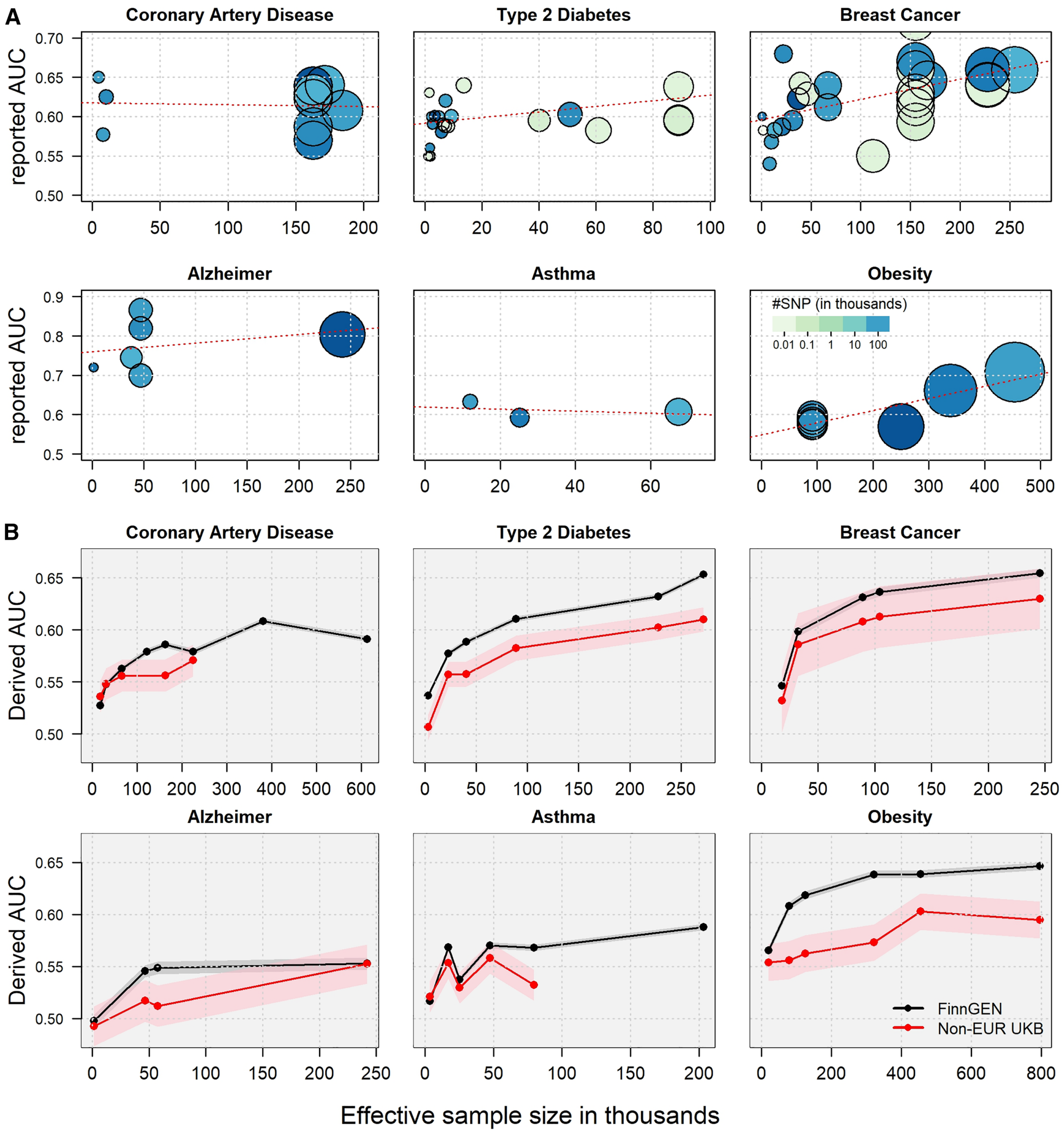

Figure 2. PRSs predictive accuracy as a function of sample size

(A) AUC reported in the literature for PRS as a function of the effective sample size across six diseases: coronary artery disease, type 2 diabetes, breast cancer, Alzheimer disease (AD), asthma, and obesity. The color gradient represents the number of variants used, ranging from a few top-associated variants (light green) to millions (dark blue), and the size is proportional to the logarithm of the effective sample size.

(B) AUCs for the 6 outcomes derived using a harmonized pipeline. PRSs were trained from 35 GWASs using the LDpred2 approach and tested with individual-level data from the FinnGen cohort (black) and in 6 non-European ancestry UKB populations (red, meta-analysis over 6 populations). The AUCs are plotted against the effective sample size of the corresponding GWAS. Missing values indicate instances where there was a sample overlap between the test and train sets. The 95% confidence intervals are shown as shaded red and gray for the UKB and FinnGen analyses, respectively.

causal genetic effects, and the dependence of those effects on LD, functional annotations, and MAF. We assessed the ability of GENESIS,[15] an approach that utilizes some of the aforementioned disease parameters, to predict the observed AUC trend using each of the 35 GWASs summary statistics. As shown in Figure S8A, the predictions diverge substantially from the trend observed in FinnGen. This is likely partly due to uncertainty in the estimated disease parameters (Figures S8B and S8C). Estimates of these parameters, obtained from alternative tools,[15,75–77,81] show similar variability depending on the input GWAS, with confidence intervals often not overlapping across GWASs for the same outcome (Figures S8 and S9; Table S6). The reasons for this variability are unclear, but until progress is made in deriving these estimates, our ability to model the convergence rate of PRS predictive power will likely remain limited, highlighting the importance of conducting retrospective studies.

We next investigated the extent to which the observed decrease in AUC improvement with sample size might be confounded by increasing heterogeneity (e.g., variability in disease definition, clinical and environmental characteristics of the participants) arising from meta-analyzing an increasing number of studies. We first compared the observed AUC trend with the trend derived using GWAS training and test data sampled exclusively from UKB participants of European ancestry, thereby reducing potential heterogeneity. We focused on obesity, one of the few scenarios that allowed for achieving a reasonably large sample size within a single homogeneous cohort. Overall, the AUC from those experiments followed the same trend as those derived in FinnGen (Figure S10) and did not suggest any effect of heterogeneity on the AUC trend in these data. We also assessed the performance of PRSs derived from the CAD GWAS in predicting seven intermediate CAD phenotypes in FinnGen. Although the absolute accuracy increased almost linearly with stricter outcome definitions in the test set, the trends remained highly consistent across all intermediate phenotypes (Figure S11).

Further direct assessment of the impact of heterogeneity is challenging, as it would require repeating the analysis from Figure 2B across homogeneous population strata and refined disease definitions. Such data are typically not available; however, some complementary indirect evaluations are possible. Heterogeneity in GWAS design and population is expected to produce heterogeneity in variant-outcome associations, such that increasing heterogeneity with sample size should lead to reduced heritability and increased polygenicity. As shown in Figures S8 and S9, we did not observe any clear evidence of such effects across the GWAS used, nor any marked difference in the trends of genetic parameter estimates when compared to the aforementioned homogeneous UKB experiment (Figures S10A–S10C).

Maximal achievable prediction

The maximum achievable AUC from a polygenic model relies primarily on variant coverage—that is, the proportion of genetic variability—and concurrently, the proportion of heritability, captured by the variants used to derive the PRS. Existing PRSs are built entirely from genome-wide genotyping arrays complemented by imputation, which offer cost-effective coverage of common genetic variants[82] in very large cohorts. This implies that even with very large GWAS sample sizes, maximum prediction accuracy is still bounded by this sub-sampling of existing variants, as compared to using whole-genome sequencing data.[65,83–87] As shown in the theoretical models from Figure 3A, the proportion of total heritability captured by genotyped variants (h_G^2) can vary substantially depending on the relationship between effect sizes and MAFs, as parametrized by the so-called alpha model[65,66] (Figures S12–S14). Using all imputed variants, including those with modest or poor imputation quality—typically filtered out in GWAS studies—can recover a substantial share of total heritability. For example, assuming a random distribution of genetic effects across the genome, h_I^2 varies from 29% for $\alpha = -1.5$ to 96% for $\alpha = 0$. In comparison, h_G^2 varies from 5% for $\alpha = -1.5$ to 90% for $\alpha = 0$. Notably, previous studies have argued that the alpha model might overestimate the effect of rare variants.[66] To address this potential limitation, we devised an attenuated alpha model that implies a reduced contribution of rare variants (Figures S12B–S12D; supplementary methods). However, when comparing these attenuated models to the baseline alpha model using real data, we found no evidence for improved fit (Figures S15 and S16).

The proportion of heritability captured can be translated into maximum achievable AUC. As expected, this maximum increases with higher heritability and lower disease prevalence[14] (Figure 3B). We compared the expected maximum AUC in real data for each of the six diseases using three heritability estimates: twins study heritability ($AUC_{max\text{-}twin}$), GWAS-based heritability derived from five approaches ($AUC_{max\text{-}GWAS}$; Table S7), and heritability captured by all imputed variants, regardless of their imputation quality ($AUC_{max\text{-}Imputed}$). Figure 3C presents all estimates in a single panel. First, the $AUC_{max\text{-}GWAS}$ estimates are relatively close to the AUCs reported in recent studies (AUC_{curr}; Table S7) for CAD, breast cancer, AD, and obesity, confirming that increasing GWAS sample size for these outcomes is unlikely to dramatically improve prediction performance. Conversely, AUC_{curr} for T2D and asthma display sizable gaps compared to $AUC_{max\text{-}GWAS}$, suggesting that despite the trend observed in Figure 2B, future GWASs with larger sample sizes may still provide a slow but continuous improvement in prediction. Second, the gap between $AUC_{max\text{-}twin}$ and $AUC_{max\text{-}GWAS}$ is large for all outcomes except for breast cancer, suggesting that increasing variant coverage in future studies could dramatically improve the predictive power of PRSs for the former outcomes. Third, $AUC_{max\text{-}Imputed}$ is substantially higher than $AUC_{max\text{-}GWAS}$ for all outcomes except breast cancer. Hence, future PRSs using an increasing number of imputed variants, even those with poor imputation quality, have the potential to boost predictive power without requiring costly sequencing data. As a sensitivity analysis, we also re-estimated all maximum achievable AUC values using prevalence data from other sources and found no qualitative impact on the results (Figure S17).

Effect distributions and predicted performances

In previous analyses, we assumed that causal variants were randomly distributed across the genome and modeled only the relationship between effect sizes and MAFs. However, there is strong evidence that causal variants are highly enriched in certain functionally annotated regions.[88–91] Modeling this conditional distribution may affect both the convergence of the AUC and the expected maximum achievable AUC. Nonetheless, the precise relationship

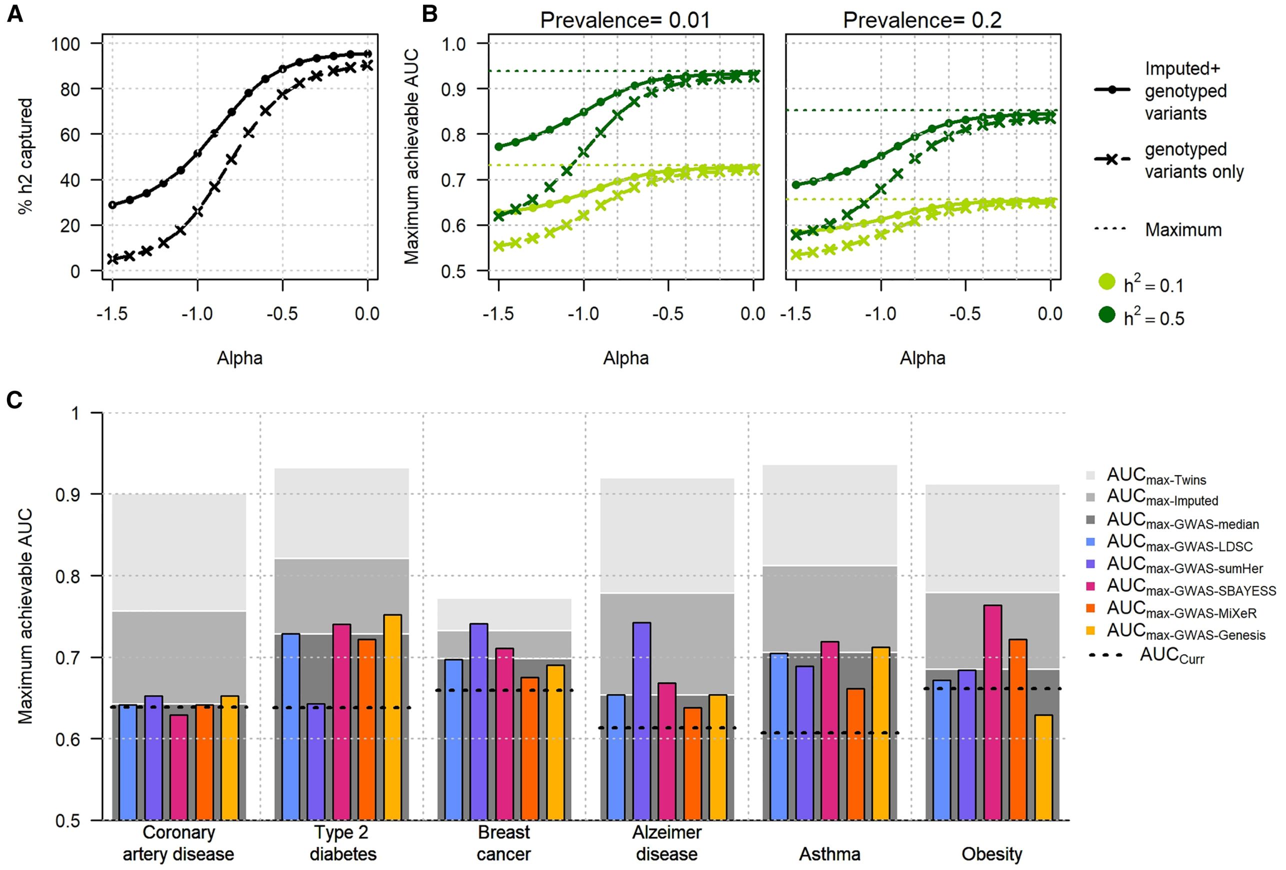

Figure 3. Heritability captured and maximum achievable AUC

(A) The expected proportion of heritability captured by genotyped variants only (dashed line) and by both genotyped and all imputed variants, regardless of their imputation quality (solid line), conditional on the minor allele frequency (MAF)-effect size relationship (alpha). Individual-level data from the UKB were used to estimate the squared correlation between sequenced variants and either genotyped variants (ρ^2) or imputed variants (r^2_{imput}). Genetic effects were assumed to be distributed following an alpha model.

(B) The corresponding maximum achievable AUC for heritability of 0.1 and 0.5 and disease prevalences of 0.01 and 0.2.

(C) Estimates of the maximum achievable AUC for six outcomes: coronary artery disease, type 2 diabetes, breast cancer, AD, asthma, and obesity (using body mass index GWAS). These AUCs were derived based on US disease prevalence and various heritability estimates: twins studies (AUC_{Twin}), heritability derived using five competitive approaches (LDSC regression, sumHer, SBayesS, GENESIS, and MiXer, labeled AUC_{GWAS}) applied to the largest GWAS available for each disease, and twins study heritability captured by imputed variants ($AUC_{Imputed}$). The black dashed lines indicate the most recent AUC estimates from real data in the literature, derived from approximately 1 million HapMap3 variants. For AD, heritability estimates and current AUC estimates exclude the *APOE* region.

between functional annotations and causal variants is not yet fully understood and likely depends on the specific outcome studied. Instead, we estimated the association between the quality of imputation and 1,099 annotations (Figure S18; Tables S8 and S9), including gene elements, DHSs, enhancers, and promoters. These annotations cover 0.001%–64.7% of the whole genome (Table S9).

The strongest associations with imputation quality were observed for gene elements (Figures 4A and S19A). CDSs were significantly negatively associated with r^2_{imput} ($p = 1.8 \times 10^{-307}$), with average r^2_{imput} values of 0.27 and 0.40 for CDSs and non-CDS variants, respectively. Exonic variants had lower imputation quality ($p = 2.0 \times 10^{-131}$), with average r^2_{imput} values of 0.37 and 0.40 for exonic and non-exonic variants, respectively. Other annotation categories exhibited weaker but statistically significant negative and positive associations (Figure 4B). Enhancers, super-enhancers, DHSs, and TFBSs generally showed reduced imputation quality compared to the average of the genome, while variants within promoters tended to have slightly higher imputation accuracy. No clear enrichment was observed for specific cell types or tissues among other top significant annotations (Figure S20). The observed reduction in imputation quality within certain annotations may partly reflect an enrichment for rare variants. For example, coding regions displayed a significantly higher proportion of rare variants ($p = 2.5 \times 10^{-270}$; Figure 4C; Table S10). As discussed in prior research, causal variants may have lower LD with neighboring variants because of selective pressure,[86,90] an assumption that is now commonly incorporated in disease parameters estimation tools.[92] Lower LD leads to lower r^2_{imput}, meaning the observed lower coverage in CDSs, enhancers, DHSs, and TFBSs may indicate an

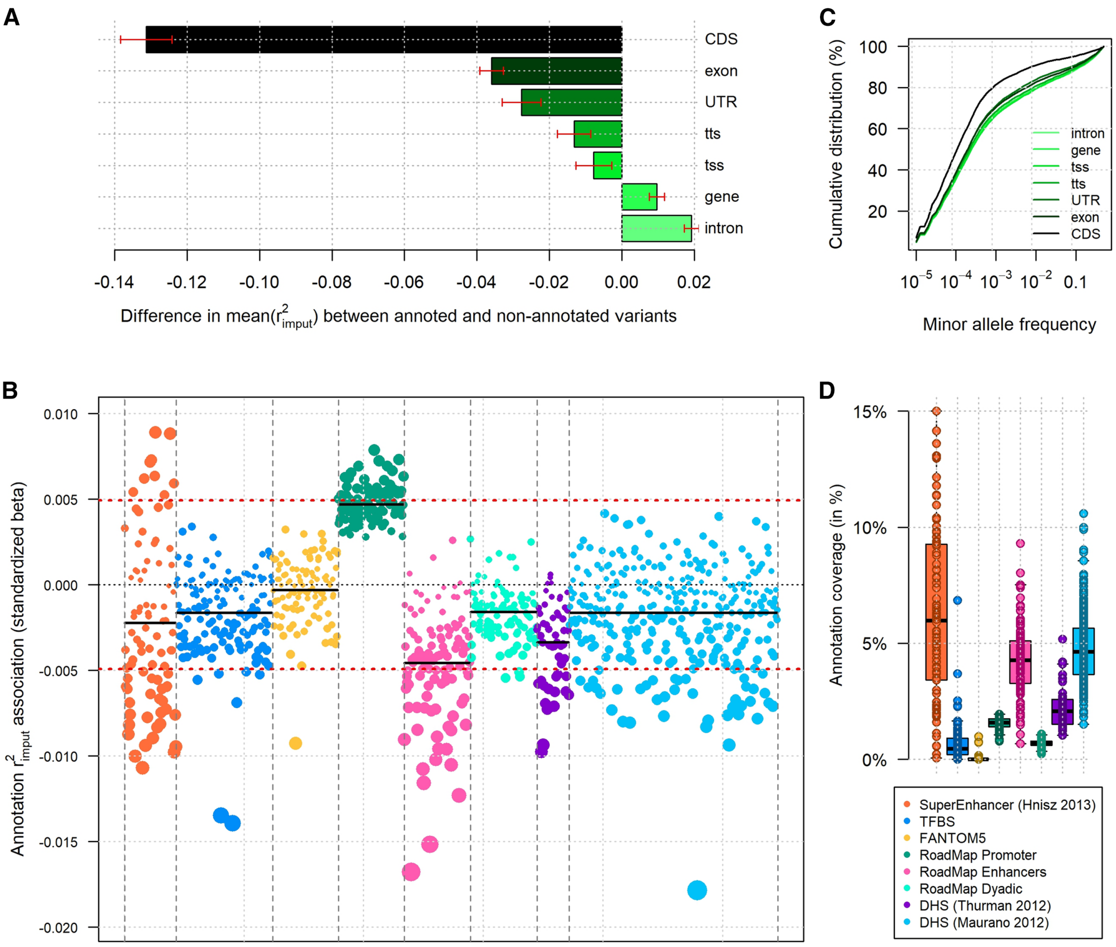

Figure 4. Imputation quality varies across functional annotations

(A–D) We computed the relationship between functional annotations and imputation quality, measured as the squared correlation (r^2_{imput}) between true and imputed genotypes.

(A) The difference in the average r^2_{imput} for chromosome 22 across GENCODE annotations: introns, genes, exons, CDS (coding DNA sequence), tss (transcription start site), tts (transcription termination site), and UTR (untranslated region). Red bars indicate ± 2 SDs from the mean, encompassing 95% of the annotated regions.

(B) Standardized regression coefficients from the univariate association between the measured r^2_{imput} and each of the 1,092 functional annotations across 8 categories: TFBS (transcription factor binding site), FANTOM5 (functional annotation of the mammalian genome version 5) regulatory regions, promoters, enhancers, and dyadic regions from Roadmap, DHS (DNase I hypersensitive sites) derived from two studies, and super-enhancers. Horizontal black lines represent the average per category, and the red dashed lines indicate the significance threshold after correction for multiple testing.

(C) The cumulative distribution of variants for each GENCODE category as a function of the MAF.

(D) The distribution of annotation frequencies, grouped by category.

enrichment for rare, poorly tagged causal variants in these regions. In such scenarios, our estimates of the maximum achievable AUC from imputed variants (Figure 3) could be slightly overestimated. These findings further suggest that improving variant coverage, especially in underrepresented functional regions, will be crucial for advancing PRS predictive accuracy in future studies (Figure S20).

Discussion

Polygenic risk prediction holds significant potential to transform the diagnosis, treatment, and prevention of many common human diseases. However, the timescale and magnitude of this transformation remain uncertain, and concerns persist within the scientific community regarding the practical relevance of the PRS. These concerns are partly due to inconsistencies in reported performances metrics. Many existing studies lack complete documentation, and while guidelines have been proposed,[17–19] adherence to these guidelines remains limited. Heterogeneity in methodology, study populations, and included covariates often impedes replication and complicates formal comparisons of PRS performance. As demonstrated in this study using six outcomes, even rigorous curation of published results may not be sufficient to clarify the relationship between prediction performance and study parameters.

Here, we demonstrate that the relationship between PRS prediction accuracy and GWAS sample size is unequivocal

and highly replicable when using harmonized data preprocessing and analysis pipelines. Our results show that the accuracy of PRSs derived from existing GWASs has begun to plateau for most studied diseases. As a consequence, expanding current efforts (i.e., solely increasing GWAS sample sizes) may result in only modest improvements in predictive power. For some outcomes, including CAD, breast cancer, and obesity, GWAS-based PRS performance appears to be nearing the expected maximum based on GWAS-derived heritability. For type 2 diabetes and asthma, a larger gap remains between current and theoretical maximum AUC values, suggesting that further gains are still achievable, albeit requiring very large GWAS sample sizes. Our analyses also suggest that without large-scale whole-genome sequencing data, the ceiling for prediction accuracy in GWAS-based PRS will remain constrained by the variant coverage of GWAS array. Importantly, this study supports previous findings[85] advocating for the inclusion of both rare and common imputed variants with modest imputation quality. These can serve as an effective intermediate strategy to enhance PRS performance, although this will require substantial methodological development.

Several other factors could further improve PRS predictive power and raise the ceiling on maximum achievable AUC. First, the observed plateau in AUC may partly reflect growing heterogeneity in large meta-analyses (e.g., looser disease definitions or more genetically diverse populations), undertaken to allow for a broader inclusion of participants. Although our results did not show strong evidence of such an effect across the studied outcomes, the influence of heterogeneity in genetic ancestry[61,79] and phenotype definition[80,93] is well documented. Second, we assumed homogeneity of genetic effects across individuals. Future PRSs may benefit from accounting for potential effect heterogeneity linked to demographic characteristics, basic health parameters, and lifestyle.[7,94,95] For instance, heritability has been shown to vary with age and sex.[96,97] Third, both the PRSs and their maximum expected AUCs depend on estimates of genetic architecture parameters. As illustrated in the present study, estimates of heritability, polygenicity, and the effect size-MAF relationship (alpha) vary substantially across methods and input GWASs. Resolving these inconsistencies will be key to improving both PRS construction and the modeling of their predictive ceilings. Additionally, we had to perform extensive preprocessing of GWASs summary statistics. Improved quality control and variants filtering[60,98] may also yield more reliable parameter estimates.

This study has several limitations in its design. First, we assumed that causal variants are randomly distributed across the genome and only modeled the relationship between MAF and effect size. This assumption is likely an oversimplification of the true underlying model, as prior research has shown enrichment of causal variants in specific LD patterns and functional annotations.[15,16,65,78,99,100] Rather than investigating specific models, we examined the relationship between variant coverage and functional annotations, observing both positive and negative enrichment across features. However, except for CDSs that show substantially poorer coverage, these differences did not qualitatively affect our conclusions. Second, we focused on PRSs derived from standard univariate GWASs and did not consider more recent, potentially more powerful methods, such as multi-ancestry[20–24] or multi-phenotype approaches.[25–27] These methods may increase power by improving effect size estimation, potentially mimicking an effective sample size increase. However, they are not likely to alter the fundamental convergence pattern toward the theoretical maximum AUC. Third, our models included PRSs only and did not incorporate additional non-genetic risk factors. Thus, the reported AUCs reflect only the marginal predictive power of polygenic risk models. In practice, the incremental value of a PRS depends on how much predictive power is already captured by conventional clinical risk factors and their correlation with the PRS.[101,102] Fourth, for real-data heritability estimates, we relied on twins studies as proxies for total additive genetic variance. These estimates may be biased due to shared environmental effects and non-additive interactions, such as gene-gene and gene-environment effects.[103] Fifth, we used prevalent cases for our evaluations. Recent research suggests that PRS accuracy may differ when predicting incident cases across various follow-up periods, potentially affecting PRS performance trends.[104]

This study also has limitations in scope. It includes only six diseases. Although we considered additional conditions (e.g., chronic obstructive pulmonary disease, Crohn disease, hypertension), data availability was insufficient to support rigorous analysis. Future works could investigate whether the trends observed here generalize to other diseases and quantitative traits. Finally, our analysis focused on PRSs derived from GWASs that included predominantly European ancestry participants. This limitation was dictated by the current imbalance in genetic study data across populations.[105] The extent to which our results apply to other ancestries remains uncertain. However, our analysis of UKB participants of non-European genetic ancestry (Figure 2B), along with recent work on PRS portability,[61,79] suggests that similar trends are likely. Nonetheless, assembling GWASs of large sample sizes in non-European populations remains essential. PRS performance is currently much lower in these groups, and increasing their representation is crucial for improving prediction.[105]

Data and code availability

Individual-level data from the UKB were accessed from the UKB Resource under application nos. 42260 and 66995. Individual-level data from FinnGen were conducted by coauthors from the University of Helsinki with privileged access.

All GWAS summary statistics have been downloaded from publicly available websites including dedicated page from consortia,

the NHGRI-EBI Catalog of human genome-wide association studies, and the FinnGen GWAS repository.

CDC disease prevalence: https://www.cdc.gov/datastatistics/index.html

FinnGen results: https://risteys.finngen.fi

Functional annotations: https://github.com/gkichaev/PAINTOR_V3.0/wiki/2b.-Overlapping-annotations

GWAS catalog: https://www.ebi.ac.uk/gwas

HapMap3: https://www.sanger.ac.uk/resources/downloads/human/hapmap3.html

All analyses were conducted using existing open-source software programs, which are freely available from the following URLs:

GCTA: https://yanglab.westlake.edu.cn/software/gcta/

GENESIS: https://github.com/yandorazhang/GENESIS

LDpred2: https://privefl.github.io/bigsnpr/articles/LDpred2.html

LDSC regression: https://github.com/bulik/ldsc

MiXeR: https://github.com/precimed/mixer

SBayesS: https://cnsgenomics.com/software/gctb/

sumHer: https://dougspeed.com/sumher

During the preparation of this work, the authors used GPT-4o to correct grammatical and syntax errors. After using this tool, the authors reviewed and edited the content as needed and take full responsibility for the content of the publication.

Acknowledgments

We want to acknowledge the participants and investigators of the FinnGen study and the UKB cohort. This research has been conducted using the UKB Resource under application nos. 42260 and 66995. We also would like to thank the authors of GENESIS, sumHer, and SBayesS for their helpful recommendations. This work has been conducted as part of the INCEPTION program (Investissement d'Avenir grant ANR-16-CONV-0005). This research was supported by the Agence Nationale de la Recherche (grant nos. ANR-20-CE36-0009-02 and ANR-20-CE15-0012-01).

Author contributions

H.A. and L.H. conceived and supervised the project. L.H., J.K., Z. Y., S.R., and G.P. carried out the primary analyses. C.A., C.B., H.J., A.F., and Y.S. conducted the secondary analyses. Z.Y. and A.G. led the analyses involving individual-level data from FinnGen. S.R., O.D., and L.H. led the analysis involving individual-level sequencing data from the UKB. L.H., F.P., B.V., C.A., and H.A. designed the LDpred2 pipeline for PRS analysis. H.A. and L.H. co-wrote the manuscript, with input from all other authors. All authors contributed to discussions.

Declaration of interests

B.V. serves on Allelica's international advisory board.

Supplemental information

Supplemental information can be found online at https://doi.org/10.1016/j.xhgg.2025.100457.

Received: April 3, 2025
Accepted: May 12, 2025

References

1. Kullo, I.J., Lewis, C.M., Inouye, M., Martin, A.R., Ripatti, S., and Chatterjee, N. (2022). Polygenic scores in biomedical research. Nat. Rev. Genet. *23*, 524–532.
2. Wald, N.J., and Old, R. (2019). The illusion of polygenic disease risk prediction. Genet. Med. *21*, 1705–1707.
3. Polygenic Risk Score Task Force of the International Common Disease Alliance (2021). Responsible use of polygenic risk scores in the clinic: potential benefits, risks and gaps. Nat. Med. *27*, 1876–1884.
4. Sud, A., Turnbull, C., and Houlston, R. (2021). Will polygenic risk scores for cancer ever be clinically useful? NPJ Precis. Oncol. *5*, 40.
5. Shendure, J., Findlay, G.M., and Snyder, M.W. (2019). Genomic Medicine-Progress, Pitfalls, and Promise. Cell *177*, 45–57.
6. Torkamani, A., Wineinger, N.E., and Topol, E.J. (2018). The personal and clinical utility of polygenic risk scores. Nat. Rev. Genet. *19*, 581–590.
7. Lewis, C.M., and Vassos, E. (2020). Polygenic risk scores: from research tools to clinical instruments. Genome Med. *12*, 44.
8. Hingorani, A.D., Gratton, J., Finan, C., Schmidt, A.F., Patel, R., Sofat, R., Kuan, V., Langenberg, C., Hemingway, H., Morris, J.K., and Wald, N.J. (2023). Performance of polygenic risk scores in screening, prediction, and risk stratification: secondary analysis of data in the Polygenic Score Catalog. BMJ Med. *2*, e000554.
9. Dudbridge, F. (2013). Power and predictive accuracy of polygenic risk scores. PLoS Genet. *9*, e1003348.
10. Chatterjee, N., Shi, J., and García-Closas, M. (2016). Developing and evaluating polygenic risk prediction models for stratified disease prevention. Nat. Rev. Genet. *17*, 392–406.
11. Privé, F., Arbel, J., and Vilhjálmsson, B.J. (2021). LDpred2: better, faster, stronger. Bioinforma. Oxf. Engl. *36*, 5424–5431.
12. Janssens, A.C.J.W., Aulchenko, Y.S., Elefante, S., Borsboom, G.J.J.M., Steyerberg, E.W., and van Duijn, C.M. (2006). Predictive testing for complex diseases using multiple genes: fact or fiction? Genet. Med. *8*, 395–400.
13. Witte, J.S., Visscher, P.M., and Wray, N.R. (2014). The contribution of genetic variants to disease depends on the ruler. Nat. Rev. Genet. *15*, 765–776.
14. Wray, N.R., Yang, J., Goddard, M.E., and Visscher, P.M. (2010). The genetic interpretation of area under the ROC curve in genomic profiling. PLoS Genet. *6*, e1000864.
15. Zhang, Y., Qi, G., Park, J.H., and Chatterjee, N. (2018). Estimation of complex effect-size distributions using summary-level statistics from genome-wide association studies across 32 complex traits. Nat. Genet. *50*, 1318–1326.
16. Zhang, Y.D., Hurson, A.N., Zhang, H., Choudhury, P.P., Easton, D.F., Milne, R.L., Simard, J., Hall, P., Michailidou, K., Dennis, J., et al. (2020). Assessment of polygenic architecture and risk prediction based on common variants across fourteen cancers. Nat. Commun. *11*, 3353.
17. Wand, H., Lambert, S.A., Tamburro, C., Iacocca, M.A., O'Sullivan, J.W., Sillari, C., Kullo, I.J., Rowley, R., Dron, J.S., Brockman, D., et al. (2021). Improving reporting standards for polygenic scores in risk prediction studies. Nature *591*, 211–219.

18. Choi, S.W., Mak, T.S.H., and O'Reilly, P.F. (2020). Tutorial: a guide to performing polygenic risk score analyses. Nat. Protoc. *15*, 2759–2772.
19. Janssens, A.C.J.W., Ioannidis, J.P.A., Bedrosian, S., Boffetta, P., Dolan, S.M., Dowling, N., Fortier, I., Freedman, A.N., Grimshaw, J.M., Gulcher, J., et al. (2011). Strengthening the reporting of genetic risk prediction studies (GRIPS): explanation and elaboration. Eur. J. Hum. Genet. *19*, 18–494.
20. Tsuo, K., Zhou, W., Wang, Y., Kanai, M., Namba, S., Gupta, R., Majara, L., Nkambule, L.L., Morisaki, T., Okada, Y., et al. (2022). Multi-ancestry meta-analysis of asthma identifies novel associations and highlights the value of increased power and diversity. Cell Genom. *2*, 100212.
21. Graham, S.E., Clarke, S.L., Wu, K.H.H., Kanoni, S., Zajac, G. J.M., Ramdas, S., Surakka, I., Ntalla, I., Vedantam, S., Winkler, T.W., et al. (2021). The power of genetic diversity in genome-wide association studies of lipids. Nature *600*, 675–679.
22. Zhang, H., Zhan, J., Jin, J., Zhang, J., Lu, W., Zhao, R., Ahearn, T.U., Yu, Z., O'Connell, J., Jiang, Y., et al. (2023). A new method for multiancestry polygenic prediction improves performance across diverse populations. Nat. Genet. *55*, 1757–1768.
23. Aragam, K.G., Jiang, T., Goel, A., Kanoni, S., Wolford, B.N., Atri, D.S., Weeks, E.M., Wang, M., Hindy, G., Zhou, W., et al. (2022). Discovery and systematic characterization of risk variants and genes for coronary artery disease in over a million participants. Nat. Genet. *54*, 1803–1815.
24. Ruan, Y., Lin, Y.F., Feng, Y.C.A., Chen, C.Y., Lam, M., Guo, Z., Stanley Global Asia Initiatives, He, L., Sawa, A., Martin, A.R., et al. (2022). Improving polygenic prediction in ancestrally diverse populations. Nat. Genet. *54*, 573–580.
25. Turley, P., Walters, R.K., Maghzian, O., Okbay, A., Lee, J.J., Fontana, M.A., Nguyen-Viet, T.A., Wedow, R., Zacher, M., Furlotte, N.A., et al. (2018). Multi-trait analysis of genome-wide association summary statistics using MTAG. Nat. Genet. *50*, 229–237.
26. Albinana, C., Zhu, Z., Schork, A.J., Ingason, A., Aschard, H., Brikell, I., Bulik, C.M., Petersen, L.V., Agerbo, E., Grove, J., et al. (2023). Multi-PGS enhances polygenic prediction by combining 937 polygenic scores. Nat. Commun. *14*, 4702.
27. Dahl, A., Thompson, M., An, U., Krebs, M., Appadurai, V., Border, R., Bacanu, S.A., Werge, T., Flint, J., Schork, A.J., et al. (2023). Phenotype integration improves power and preserves specificity in biobank-based genetic studies of major depressive disorder. Nat. Genet. *55*, 2082–2093.
28. Gao, B., Huang, Q., and Baudis, M. (2018). segment_liftover: a Python tool to convert segments between genome assemblies. F1000Res. *7*, 319.
29. Willer, C.J., Li, Y., and Abecasis, G.R. (2010). METAL: fast and efficient meta-analysis of genomewide association scans. Bioinformatics *26*, 2190–2191.
30. Coronary Artery Disease Genetics Consortium. (2011). A genome-wide association study in Europeans and South Asians identifies five new loci for coronary artery disease. Nat. Genet. *43*, 339–344.
31. Schunkert, H., König, I.R., Kathiresan, S., Reilly, M.P., Assimes, T.L., Holm, H., Preuss, M., Stewart, A.F.R., Barbalic, M., Gieger, C., et al. (2011). Large-scale association analysis identifies 13 new susceptibility loci for coronary artery disease. Nat. Genet. *43*, 333–338.
32. Nikpay, M., Goel, A., Won, H.-H., Hall, L.M., Willenborg, C., Kanoni, S., Saleheen, D., Kyriakou, T., Nelson, C.P., Hopewell, J.C., et al. (2015). A comprehensive 1,000 Genomes-based genome-wide association meta-analysis of coronary artery disease. Nat. Genet. *47*, 1121–1130.
33. Nelson, C.P., Goel, A., Butterworth, A.S., Kanoni, S., Webb, T.R., Marouli, E., Zeng, L., Ntalla, I., Lai, F.Y., Hopewell, J.C., et al. (2017). Association analyses based on false discovery rate implicate new loci for coronary artery disease. Nat. Genet. *49*, 1385–1391.
34. van der Harst, P., and Verweij, N. (2018). Identification of 64 Novel Genetic Loci Provides an Expanded View on the Genetic Architecture of Coronary Artery Disease. Circ. Res. *122*, 433–443.
35. Cai, L., Wheeler, E., Kerrison, N.D., Luan, J., Deloukas, P., Franks, P.W., Amiano, P., Ardanaz, E., Bonet, C., Fagherazzi, G., et al. (2020). Genome-wide association analysis of type 2 diabetes in the EPIC-InterAct study. Sci. Data *7*, 393.
36. Morris, A.P., Voight, B.F., Teslovich, T.M., Ferreira, T., Segrè, A.V., Steinthorsdottir, V., Strawbridge, R.J., Khan, H., Grallert, H., Mahajan, A., et al. (2012). Large-scale association analysis provides insights into the genetic architecture and pathophysiology of type 2 diabetes. Nat. Genet. *44*, 981–990.
37. Scott, R.A., Scott, L.J., Mägi, R., Marullo, L., Gaulton, K.J., Kaakinen, M., Pervjakova, N., Pers, T.H., Johnson, A.D., Eicher, J.D., et al. (2017). An Expanded Genome-Wide Association Study of Type 2 Diabetes in Europeans. Diabetes *66*, 2888–2902.
38. Xue, A., Wu, Y., Zhu, Z., Zhang, F., Kemper, K.E., Zheng, Z., Yengo, L., Lloyd-Jones, L.R., Sidorenko, J., Wu, Y., et al. (2018). Genome-wide association analyses identify 143 risk variants and putative regulatory mechanisms for type 2 diabetes. Nat. Commun. *9*, 2941.
39. Mahajan, A., Taliun, D., Thurner, M., Robertson, N.R., Torres, J.M., Rayner, N.W., Payne, A.J., Steinthorsdottir, V., Scott, R.A., Grarup, N., et al. (2018). Fine-mapping type 2 diabetes loci to single-variant resolution using high-density imputation and islet-specific epigenome maps. Nat. Genet. *50*, 1505–1513.
40. Michailidou, K., Beesley, J., Lindstrom, S., Canisius, S., Dennis, J., Lush, M.J., Maranian, M.J., Bolla, M.K., Wang, Q., Shah, M., et al. (2015). Genome-wide association analysis of more than 120,000 individuals identifies 15 new susceptibility loci for breast cancer. Nat. Genet. *47*, 373–380.
41. Michailidou, K., Hall, P., Gonzalez-Neira, A., Ghoussaini, M., Dennis, J., Milne, R.L., Schmidt, M.K., Chang-Claude, J., Bojesen, S.E., Bolla, M.K., et al. (2013). Large-scale genotyping identifies 41 new loci associated with breast cancer risk. Nat. Genet. *45*, 353–361, 361e1–2.
42. Michailidou, K., Lindström, S., Dennis, J., Beesley, J., Hui, S., Kar, S., Lemaçon, A., Soucy, P., Glubb, D., Rostamianfar, A., et al. (2017). Association analysis identifies 65 new breast cancer risk loci. Nature *551*, 92–94.
43. Zhang, H., Ahearn, T.U., Lecarpentier, J., Barnes, D., Beesley, J., Qi, G., Jiang, X., O'Mara, T.A., Zhao, N., Bolla, M. K., et al. (2020). Genome-wide association study identifies 32 novel breast cancer susceptibility loci from overall and subtype-specific analyses. Nat. Genet. *52*, 572–581.
44. Li, H., Wetten, S., Li, L., St Jean, P.L., Upmanyu, R., Surh, L., Hosford, D., Barnes, M.R., Briley, J.D., Borrie, M., et al. (2008). Candidate single-nucleotide polymorphisms from

a genomewide association study of Alzheimer disease. Arch. Neurol. *65*, 45–53.

45. Lambert, J.C., Ibrahim-Verbaas, C.A., Harold, D., Naj, A.C., Sims, R., Bellenguez, C., DeStafano, A.L., Bis, J.C., Beecham, G.W., Grenier-Boley, B., et al. (2013). Meta-analysis of 74,046 individuals identifies 11 new susceptibility loci for Alzheimer's disease. Nat. Genet. *45*, 1452–1458.
46. Kunkle, B.W., Grenier-Boley, B., Sims, R., Bis, J.C., Damotte, V., Naj, A.C., Boland, A., Vronskaya, M., van der Lee, S.J., Amlie-Wolf, A., et al. (2019). Genetic meta-analysis of diagnosed Alzheimer's disease identifies new risk loci and implicates Aβ, tau, immunity and lipid processing. Nat. Genet. *51*, 414–430.
47. Jansen, I.E., Savage, J.E., Watanabe, K., Bryois, J., Williams, D.M., Steinberg, S., Sealock, J., Karlsson, I.K., Hägg, S., Athanasiu, L., et al. (2019). Genome-wide meta-analysis identifies new loci and functional pathways influencing Alzheimer's disease risk. Nat. Genet. *51*, 404–413.
48. Shrine, N., Portelli, M.A., John, C., Artigas, M.S., Bennett, N., Hall, R., Lewis, J., Henry, A.P., Billington, C.K., Ahmad, A., et al. (2019). Moderate-to-severe asthma in individuals of European ancestry: a genome-wide association study. Lancet Respir. Med. *7*, 20–34.
49. Moffatt, M.F., Gut, I.G., Demenais, F., Strachan, D.P., Bouzigon, E., Heath, S., von Mutius, E., Farrall, M., Lathrop, M., Cookson, W.O.C.M., et al. (2010). A large-scale, consortium-based genomewide association study of asthma. N. Engl. J. Med. *363*, 1211–1221.
50. Zhu, Z., Lee, P.H., Chaffin, M.D., Chung, W., Loh, P.-R., Lu, Q., Christiani, D.C., and Liang, L. (2018). A genome-wide cross-trait analysis from UK Biobank highlights the shared genetic architecture of asthma and allergic diseases. Nat. Genet. *50*, 857–864.
51. Demenais, F., Margaritte-Jeannin, P., Barnes, K.C., Cookson, W.O.C., Altmüller, J., Ang, W., Graham Barr, R., Beaty, T.H., Becker, A.B., Beilby, J., et al. (2018). Multiancestry association study identifies new asthma risk loci that colocalize with immune-cell enhancer marks. Nat. Genet. *50*, 42–53.
52. Han, Y., Jia, Q., Jahani, P.S., Hurrell, B.P., Pan, C., Huang, P., Gukasyan, J., Woodward, N.C., Eskin, E., Gilliland, F.D., et al. (2020). Genome-wide analysis highlights contribution of immune system pathways to the genetic architecture of asthma. Nat. Commun. *11*, 1776.
53. Speliotes, E.K., Willer, C.J., Berndt, S.I., Monda, K.L., Thorleifsson, G., Jackson, A.U., Allen, H.L., Lindgren, C.M., Luan, J., Mägi, R., et al. (2010). Association analyses of 249,796 individuals reveal 18 new loci associated with body mass index. Nat. Genet. *42*, 937–948.
54. Locke, A.E., Kahali, B., Berndt, S.I., Justice, A.E., Pers, T.H., Day, F.R., Powell, C., Vedantam, S., Buchkovich, M.L., Yang, J., et al. (2015). Genetic studies of body mass index yield new insights for obesity biology. Nature *518*, 197–206.
55. Yengo, L., Sidorenko, J., Kemper, K.E., Zheng, Z., Wood, A. R., Weedon, M.N., Frayling, T.M., Hirschhorn, J., Yang, J., and Visscher, P.M. (2018). Meta-analysis of genome-wide association studies for height and body mass index in ∼700000 individuals of European ancestry. Hum. Mol. Genet. *27*, 3641–3649.
56. Elsworth, B., Lyon, M., Alexander, T., Liu, Y., Matthews, P., Hallett, J., Bates, P., Palmer, T., Haberland, V., Smith, G.D., et al. (2020). The MRC IEU OpenGWAS data infrastructure. Preprint at bioRxiv. https://doi.org/10.1101/2020.08.10.244293.
57. Wang, Y., Namba, S., Lopera, E., Kerminen, S., Tsuo, K., Läll, K., Kanai, M., Zhou, W., Wu, K.H., Favé, M.J., et al. (2023). Global Biobank analyses provide lessons for developing polygenic risk scores across diverse cohorts. Cell Genom. *3*, 100241.
58. Ge, T., Chen, C.Y., Ni, Y., Feng, Y.C.A., and Smoller, J.W. (2019). Polygenic prediction via Bayesian regression and continuous shrinkage priors. Nat. Commun. *10*, 1776.
59. International HapMap 3 Consortium, Altshuler, D.M., Gibbs, R.A., Peltonen, L., Altshuler, D.M., Gibbs, R.A., Peltonen, L., Dermitzakis, E., Schaffner, S.F., Yu, F., et al. (2010). Integrating common and rare genetic variation in diverse human populations. Nature *467*, 52–58.
60. Privé, F., Arbel, J., Aschard, H., and Vilhjálmsson, B.J. (2022). Identifying and correcting for misspecifications in GWAS summary statistics and polygenic scores. HGG Adv. *3*, 100136.
61. Privé, F., Aschard, H., Carmi, S., Folkersen, L., Hoggart, C., O'Reilly, P.F., and Vilhjálmsson, B.J. (2022). Portability of 245 polygenic scores when derived from the UK Biobank and applied to 9 ancestry groups from the same cohort. Am. J. Hum. Genet. *109*, 373.
62. Delaneau, O., Zagury, J.F., Robinson, M.R., Marchini, J.L., and Dermitzakis, E.T. (2019). Accurate, scalable and integrative haplotype estimation. Nat. Commun. *10*, 5436.
63. Zaitlen, N., and Kraft, P. (2012). Heritability in the genome-wide association era. Hum. Genet. *131*, 1655–1664.
64. Bycroft, C., Freeman, C., Petkova, D., Band, G., Elliott, L.T., Sharp, K., Motyer, A., Vukcevic, D., Delaneau, O., O'Connell, J., et al. (2018). The UK Biobank resource with deep phenotyping and genomic data. Nature *562*, 203–209.
65. Speed, D., Cai, N., UCLEB Consortium, Johnson, M.R., Nejentsev, S., and Balding, D.J. (2017). Reevaluation of SNP heritability in complex human traits. Nat. Genet. *49*, 986–992.
66. Schoech, A.P., Jordan, D.M., Loh, P.R., Gazal, S., O'Connor, L.J., Balick, D.J., Palamara, P.F., Finucane, H.K., Sunyaev, S. R., and Price, A.L. (2019). Quantification of frequency-dependent genetic architectures in 25 UK Biobank traits reveals action of negative selection. Nat. Commun. *10*, 790.
67. Marouli, E., Graff, M., Medina-Gomez, C., Lo, K.S., Wood, A. R., Kjaer, T.R., Fine, R.S., Lu, Y., Schurmann, C., Highland, H.M., et al. (2017). Rare and low-frequency coding variants alter human adult height. Nature *542*, 186–190.
68. Dai, X., Wiernek, S., Evans, J.P., and Runge, M.S. (2016). Genetics of coronary artery disease and myocardial infarction. World J. Cardiol. *8*, 1–23.
69. Willemsen, G., Ward, K.J., Bell, C.G., Christensen, K., Bowden, J., Dalgård, C., Harris, J.R., Kaprio, J., Lyle, R., Magnusson, P.K.E., et al. (2015). The Concordance and Heritability of Type 2 Diabetes in 34,166 Twin Pairs From International Twin Registers: The Discordant Twin (DISCOTWIN) Consortium. Twin Res. Hum. Genet. *18*, 762–771.
70. Lichtenstein, P., Holm, N.V., Verkasalo, P.K., Iliadou, A., Kaprio, J., Koskenvuo, M., Pukkala, E., Skytthe, A., and Hemminki, K. (2000). Environmental and heritable factors in the causation of cancer–analyses of cohorts of twins from Sweden, Denmark, and Finland. N. Engl. J. Med. *343*, 78–85.
71. Gatz, M., Pedersen, N.L., Berg, S., Johansson, B., Johansson, K., Mortimer, J.A., Posner, S.F., Viitanen, M., Winblad, B.,

and Ahlbom, A. (1997). Heritability for Alzheimer's disease: the study of dementia in Swedish twins. J. Gerontol. A Biol. Sci. Med. Sci. *52*, M117–M125.
72. Karlsson, I.K., Escott-Price, V., Gatz, M., Hardy, J., Pedersen, N.L., Shoai, M., and Reynolds, C.A. (2022). Measuring heritable contributions to Alzheimer's disease: polygenic risk score analysis with twins. Brain Commun. *4*, fcab308.
73. Thomsen, S.F. (2015). The contribution of twin studies to the understanding of the aetiology of asthma and atopic diseases. Eur. Clin. Respir. J. *2*, 27803.
74. Elks, C.E., den Hoed, M., Zhao, J.H., Sharp, S.J., Wareham, N.J., Loos, R.J.F., and Ong, K.K. (2012). Variability in the heritability of body mass index: a systematic review and meta-regression. Front. Endocrinol. *3*, 29.
75. Zeng, J., Xue, A., Jiang, L., Lloyd-Jones, L.R., Wu, Y., Wang, H., Zheng, Z., Yengo, L., Kemper, K.E., Goddard, M.E., et al. (2021). Widespread signatures of natural selection across human complex traits and functional genomic categories. Nat. Commun. *12*, 1164.
76. Speed, D., and Balding, D.J. (2019). SumHer better estimates the SNP heritability of complex traits from summary statistics. Nat. Genet. *51*, 277–284.
77. Bulik-Sullivan, B.K., Loh, P.R., Finucane, H.K., Ripke, S., Yang, J., Schizophrenia Working Group of the Psychiatric Genomics Consortium, Patterson, N., Daly, M.J., Price, A. L., and Neale, B.M. (2015). LD Score regression distinguishes confounding from polygenicity in genome-wide association studies. Nat. Genet. *47*, 291–295.
78. Holland, D., Frei, O., Desikan, R., Fan, C.C., Shadrin, A.A., Smeland, O.B., Sundar, V.S., Thompson, P., Andreassen, O.A., and Dale, A.M. (2020). Beyond SNP heritability: Polygenicity and discoverability of phenotypes estimated with a univariate Gaussian mixture model. PLoS Genet. *16*, e1008612.
79. Ding, Y., Hou, K., Xu, Z., Pimplaskar, A., Petter, E., Boulier, K., Privé, F., Vilhjálmsson, B.J., Olde Loohuis, L.M., and Pasaniuc, B. (2023). Polygenic scoring accuracy varies across the genetic ancestry continuum. Nature *618*, 774–781.
80. Escott-Price, V., and Hardy, J. (2022). Genome-wide association studies for Alzheimer's disease: bigger is not always better. Brain Commun. *4*, fcac125.
81. Frei, O., Holland, D., Smeland, O.B., Shadrin, A.A., Fan, C. C., Maeland, S., O'Connell, K.S., Wang, Y., Djurovic, S., Thompson, W.K., et al. (2019). Bivariate causal mixture model quantifies polygenic overlap between complex traits beyond genetic correlation. Nat. Commun. *10*, 2417.
82. Nelson, S.C., Romm, J.M., Doheny, K.F., Pugh, E.W., and Laurie, C.C. (2017). Imputation-Based Genomic Coverage Assessments of Current Genotyping Arrays: *Illumina HumanCore, OmniExpress, Multi-Ethnic global array and sub-arrays, Global Screening Array, Omni2.5M, Omni5M, and Affymetrix UK Biobank*. Preprint at bioRxiv. https://doi.org/10.1101/150219.
83. Wainschtein, P., Jain, D., Zheng, Z., TOPMed Anthropometry Working Group, NHLBI Trans-Omics for Precision Medicine TOPMed Consortium, Cupples, L.A., Shadyab, A. H., McKnight, B., Shoemaker, B.M., Mitchell, B.D., et al. (2022). Assessing the contribution of rare variants to complex trait heritability from whole-genome sequence data. Nat. Genet. *54*, 263–273.
84. Yang, J., Benyamin, B., McEvoy, B.P., Gordon, S., Henders, A.K., Nyholt, D.R., Madden, P.A., Heath, A.C., Martin, N. G., Montgomery, G.W., et al. (2010). Common SNPs explain a large proportion of the heritability for human height. Nat. Genet. *42*, 565–569.
85. Yang, J., Bakshi, A., Zhu, Z., Hemani, G., Vinkhuyzen, A.A. E., Lee, S.H., Robinson, M.R., Perry, J.R.B., Nolte, I.M., van Vliet-Ostaptchouk, J.V., et al. (2015). Genetic variance estimation with imputed variants finds negligible missing heritability for human height and body mass index. Nat. Genet. *47*, 1114–1120.
86. Weiner, D.J., Nadig, A., Jagadeesh, K.A., Dey, K.K., Neale, B. M., Robinson, E.B., Karczewski, K.J., and O'Connor, L.J. (2023). Polygenic architecture of rare coding variation across 394,783 exomes. Nature *614*, 492–499.
87. Manolio, T.A., Collins, F.S., Cox, N.J., Goldstein, D.B., Hindorff, L.A., Hunter, D.J., McCarthy, M.I., Ramos, E.M., Cardon, L.R., Chakravarti, A., et al. (2009). Finding the missing heritability of complex diseases. Nature *461*, 747–753.
88. Finucane, H.K., Bulik-Sullivan, B., Gusev, A., Trynka, G., Reshef, Y., Loh, P.R., Anttila, V., Xu, H., Zang, C., Farh, K., et al. (2015). Partitioning heritability by functional annotation using genome-wide association summary statistics. Nat. Genet. *47*, 1228–1235.
89. Won, H.H., Natarajan, P., Dobbyn, A., Jordan, D.M., Roussos, P., Lage, K., Raychaudhuri, S., Stahl, E., and Do, R. (2015). Disproportionate Contributions of Select Genomic Compartments and Cell Types to Genetic Risk for Coronary Artery Disease. PLoS Genet. *11*, e1005622.
90. Gazal, S., Finucane, H.K., Furlotte, N.A., Loh, P.R., Palamara, P.F., Liu, X., Schoech, A., Bulik-Sullivan, B., Neale, B.M., Gusev, A., and Price, A.L. (2017). Linkage disequilibrium-dependent architecture of human complex traits shows action of negative selection. Nat. Genet. *49*, 1421–1427.
91. Roadmap Epigenomics Consortium, Kundaje, A., Meuleman, W., Ernst, J., Bilenky, M., Yen, A., Heravi-Moussavi, A., Kheradpour, P., Zhang, Z., Wang, J., et al. (2015). Integrative analysis of 111 reference human epigenomes. Nature *518*, 317–330.
92. Speed, D., Hemani, G., Johnson, M.R., and Balding, D.J. (2012). Improved heritability estimation from genome-wide SNPs. Am. J. Hum. Genet. *91*, 1011–1021.
93. Cai, N., Revez, J.A., Adams, M.J., Andlauer, T.F.M., Breen, G., Byrne, E.M., Clarke, T.K., Forstner, A.J., Grabe, H.J., Hamilton, S.P., et al. (2020). Minimal phenotyping yields genome-wide association signals of low specificity for major depression. Nat. Genet. *52*, 437–447.
94. Mostafavi, H., Harpak, A., Agarwal, I., Conley, D., Pritchard, J.K., and Przeworski, M. (2020). Variable prediction accuracy of polygenic scores within an ancestry group. eLife *9*, e48376.
95. Duncan, L., Shen, H., Gelaye, B., Meijsen, J., Ressler, K., Feldman, M., Peterson, R., and Domingue, B. (2019). Analysis of polygenic risk score usage and performance in diverse human populations. Nat. Commun. *10*, 3328.
96. Bernabeu, E., Canela-Xandri, O., Rawlik, K., Talenti, A., Prendergast, J., and Tenesa, A. (2021). Sex differences in genetic architecture in the UK Biobank. Nat. Genet. *53*, 1283–1289.
97. Ge, T., Chen, C.Y., Neale, B.M., Sabuncu, M.R., and Smoller, J.W. (2017). Phenome-wide heritability analysis of the UK Biobank. PLoS Genet. *13*, e1006711.
98. Grotzinger, A.D., Fuente, J.d.l., Privé, F., Nivard, M.G., and Tucker-Drob, E.M. (2023). Pervasive Downward Bias in Estimates of Liability-Scale Heritability in Genome-wide

Association Study Meta-analysis: A Simple Solution. Biol. Psychiatry *93*, 29–36.

99. O'Connor, L.J. (2021). The distribution of common-variant effect sizes. Nat. Genet. *53*, 1243–1249.

100. Orliac, E.J., Trejo Banos, D., Ojavee, S.E., Läll, K., Mägi, R., Visscher, P.M., and Robinson, M.R. (2022). Improving GWAS discovery and genomic prediction accuracy in Biobank data. Proc. Natl. Acad. Sci. USA *119*, e2121279119.

101. Riveros-Mckay, F., Weale, M.E., Moore, R., Selzam, S., Krapohl, E., Sivley, R.M., Tarran, W.A., Sørensen, P., Lachapelle, A.S., Griffiths, J.A., et al. (2021). Integrated Polygenic Tool Substantially Enhances Coronary Artery Disease Prediction. Circ. Genom. Precis. Med. *14*, e003304.

102. Mars, N., Koskela, J.T., Ripatti, P., Kiiskinen, T.T.J., Havulinna, A.S., Lindbohm, J.V., Ahola-Olli, A., Kurki, M., Karjalainen, J., Palta, P., et al. (2020). Polygenic and clinical risk scores and their impact on age at onset and prediction of cardiometabolic diseases and common cancers. Nat. Med. *26*, 549–557.

103. Zaitlen, N., Kraft, P., Patterson, N., Pasaniuc, B., Bhatia, G., Pollack, S., and Price, A.L. (2013). Using extended genealogy to estimate components of heritability for 23 quantitative and dichotomous traits. PLoS Genet. *9*, e1003520.

104. Tcheandjieu, C., Zhu, X., Hilliard, A.T., Clarke, S.L., Napolioni, V., Ma, S., Lee, K.M., Fang, H., Chen, F., Lu, Y., et al. (2022). Large-scale genome-wide association study of coronary artery disease in genetically diverse populations. Nat. Med. *28*, 1679–1692.

105. Martin, A.R., Kanai, M., Kamatani, Y., Okada, Y., Neale, B. M., and Daly, M.J. (2019). Clinical use of current polygenic risk scores may exacerbate health disparities. Nat. Genet. *51*, 584–591.

HGGA, Volume *6*

Supplemental information

Polygenic risk score prediction accuracy convergence

Léo Henches, Jihye Kim, Zhiyu Yang, Simone Rubinacci, Gabriel Pires, Clara Albiñana, Christophe Boetto, Hanna Julienne, Arthur Frouin, Antoine Auvergne, Yuka Suzuki, Sarah Djebali, Olivier Delaneau, Andrea Ganna, Bjarni Vilhjálmsson, Florian Privé, and Hugues Aschard

Contents

Supplementary Methods 2
Existing AUC from the literature 2
Additional GWAS within UK Biobank 2
Phenotype selection in UK Biobank and FinnGen 2
Correlation between European and FinGenn GWAS 3
Specificity of the genetic model for obesity 3
Specificity of the genetic model for Alzheimer disease 3
Parameters estimation from GWAS using existing tools 4
Simulations to study the expected convergence of PRS 5
Estimation of alpha for common diseases using UK Biobank individual-level data 6
Attenuated alpha model 6
Supplementary Figures 7
Figure S1. European and FinnGen GWAS 7
Figure S2. AUC as a function of sample size for 6 UK Biobank populations 8
Figure S3. Variability in Alzheimer prediction conditional on APOE 9
Figure S4. AUC based on PRS derived from alternative methods 10
Figure S5. Prediction accuracy measured by alternative metrics 11
Figure S6. Sample overlap and overfitting 12
Figure S7. AUC convergence using simulated causal variants 13
Figure S8. AUC increase, heritability and number of causal variants derived from GENESIS 14
Figure S9. SBayesS disease parameters estimates across the six outcomes and GWAS 15
Figure S10. Assessing the impact of GWAS heterogeneity on disease parameter estimation and AUC . 16
Figure S11. AUC across CAD phenotypes 17
Figure S12. Baseline and attenuated alpha models and proportion of heritability captured 18
Figure S13. Variance of untyped variants captured by genotyped variants 19
Figure S14. Variance explained by MAF bins 20
Figure S15. Estimation of alpha using UK Biobank individual-level data 21
Figure S16. Validation of the GRM-GCTA pipeline using simulated data 22
Figure S17. Impact of prevalence on estimated maximum achievable AUC 23
Figure S18. Correlation between functional annotations 24
Figure S19. Distribution of imputation quality across GENCODE annotations 25
Figure S20. Top annotations associated with imputation quality 26
References 27

Supplementary Methods

Existing AUC from the literature

We conducted in October 2023 a *PubMed* search for studies reporting the prediction accuracy of genetic risk score for six outcomes, coronary artery disease (CAD), breast cancer (BRCA), type 2 diabetes (T2D), Alzheimer disease (AD), asthma, and obesity using the disease name and the terms "*AUC*" and "*genetic risk score*", which we completed with *ad hoc* search terms. We conducted a first screening and filtered out irrelevant studies not reporting AUC. For the remaining ones, we carefully checked the analysis pipeline, and kept studies using i) at least 300 cases (the number of controls was always larger than the number of cases), ii) using mostly participants of European ancestry, and iii) where the AUC was derived using the genetic risk score only, excluding other covariates (*e.g.,* age, sex, etc). After this careful quality control, a total of 10, 23, 26, 6, 3 and 8 studies remained for CAD, T2D, BRCA, AD, asthma, and obesity, respectively. For each study we extracted the following parameters: the number of cases and controls of the training set used to derive the polygenic risk score, the number of genetic variants used, the reported AUC in the validation set, and the publication year. All collected information are reported in **Table S1**. Trends in AUC increase as a function of sample size was tested using a weighted linear regression as implemented in the R *lm()* function, where weights were defined as the logarithm of the effective sample size ($N_{eff} = 4\, N_{cases} \times N_{controls}\, /(N_{cases} + N_{controls})$).

Additional GWAS within UK Biobank

Consortium-based GWAS provided results for large sample size for each disease considered in this study (**Table S2**). However, we found only limited publicly available GWAS results with intermediate sample size for multiple outcomes. To complete our prediction accuracy analyses, we conducted five additional GWAS in the UK Biobank using unrelated participants of European ancestry, and cases sampled from the entire cohort: breast cancer (N_{cases}=5,000; $N_{controls}$=50,000); coronary artery disease (N_{cases}=5,000; $N_{controls}$=50,000); asthma (N_{cases}=1,000; $N_{controls}$=10,000); and BMI (N=20,000 and N=80,000). We also conducted a GWAS for obesity from the whole sample, where obesity was defined as BMI>30 (N_{cases}= 74,660 ; $N_{controls}$=238,048) for comparison purposes. All analyses were conducted using Plink2.0's --glm option and adjusted for the top 15 principal components[1], age, and sex. The GWAS only included the ~1M variants from HapMap3 used in the construction of the PRS scores. For breast cancer, we selected female cases and controls and adjusted for 15 PCs and age. For each study, we conducted a QC filtering using the default value of LDpred2 (https://privefl.github.io/bigsnpr/reference/snp_plinkQC.html). We removed all variants with a minor allele frequency (MAF) below 0.01, or Hardy-Weinberg equilibrium exact test p-value below 10^{-50}, and filtered out all SNPs and individuals with missing data over 10%.s

Phenotype selection in UK Biobank and FinnGen

For all analyses involving the UK Biobank and the FinnGen cohorts, we had to select a specific variable from all available fields for each of the six outcomes considered: type 2 diabetes (T2D), breast cancer (BRCA), coronary artery disease (CAD), Alzheimer disease (AD), asthma (AS), and body mass index (BMI)/obesity. In the FinnGen (https://risteys.finngen.fi/), we used the broadest definition of prevalent disease cases available. For type 2 diabetes, we used *Type 2 diabetes, definitions combined* (ID=T2D). For breast cancer, we used *Malignant neoplasm of breast* (ID=C3_BREAST). For CAD, we used *Major coronary heart disease event* (ID=I9_CHD). For Alzheimer, we used *Alzheimer disease* (ID=G6_ALZHEIMER). For asthma, we used *Asthma* (ID=J10_ASTHMA). For obesity, we used *Obesity* (ID=E4_OBESITY). In the UK Biobank (https://biobank.ndph.ox.ac.uk/), phenotypes were defined based on clinical ICD10 diagnosis codes (variable ID=41270) for five outcomes: breast cancer (ICD10 code=C50), coronary artery diseases (ICD10 codes=I21-I25), obesity (ICD10 code=E66), T2D (ICD10 codes=E10-E14), and asthma (ICD10 codes=E10-E14). For Alzheimer disease, we used AD-by-proxy (code 10 for variable ID=20110 from the mother and variable ID=20107 from the father), as proposed by Janssen et al[2], because the sample size for the Alzheimer disease

status was too small. Counts of cases and controls for each outcome and each population considered are provided in **Table S3.**

Correlation between European and FinGenn GWAS

The GWASs we used to derive the PRS include a vast majority of individuals of European descent (**Table S2**). Although the Finns population from the FinGenn cohort are also of European descent, they have a slight north Asian admixture[3]. To explore possible PRS portability issues[4], we compared top association results from the largest GWAS[5-8] from **Table S2** with those from the FinnGen GWAS, publicly available on their website (https://www.finngen.fi/en/access_results). For the latter, we used summary statistics from release 5, using IDs listed in the previous section. For each outcome we extracted from the corresponding European and Finns GWAS pair, all SNPs with a *p*-value below 1e-6 in either study. We created a merged list of variants and kept only those for which a *p*-value was available in both GWAS. We next clumped this subset using the *ld_clump()* function from the R package "ieugwasr" (https://rdrr.io/github/MRCIEU/ieugwasr/man/ld_clump.html) with an R^2 threshold of 0.05 and default options, and the 1000 genomes[9] as a reference panel. Using either the European *p*-values or the FinnGen *p*-values for clumping produced qualitatively similar results. Overall, when plotting the estimated regression coefficient from each pair of studies against each other, we found highly consistent results between FinnGen and European GWAS (**Figure S1**).

Specificity of the genetic model for obesity

Along the study we use body mass index (BMI) GWAS to build polygenic risk scores and estimate disease parameters for obesity (defined as BMI ≥ 30 kg/m2), because the sample size available for this phenotype is substantially higher than for GWAS of obesity. In theory, the heritability of BMI should equal the heritability of obesity on the liability scale, and the disease parameters should be fairly similar. Indeed, obesity follows the definition of the liability threshold model (LTM) that is commonly used to report the heritability of binary outcomes[10], with BMI being the liability. Briefly, the liability describes the combined risk of genetic and environmental factors that contribute to the development of a disease, with the disease status being determined by the liability reaching a threshold. We did not conduct a comparison between the two outcomes because of the lack of data for obesity, however, we did notice differences in some parameter estimations when using either BMI or obesity (e.g. **Fig. S15**), that might be investigated in future studies.

Note that for the derivation of the predicted AUC of obesity using GENESIS (see next section), we used the BMI GWAS, while the approach is expecting z-score derived from a logistic regression. We investigated the possible impact of using BMI GWAS as a proxy for obesity GWAS through simulations. We generated series of replicates where a normally distributed outcome Y depends on a single predictor X, and applied for each replicate a linear model $Y \sim \beta_0 + \beta_Y X$ and a logistic regression $logit(\Pr(D)) \sim \alpha + \beta_D X$, where D is a dichotomized version of Y, defined as $D = 1$ if Y is larger than the 70th percentile of Y and $D = 0$ otherwise (mimicking the BMI-obesity relationship). We compared the z-scores from the two models $z_Y = \hat{\beta}_Y / \sigma_{\hat{\beta}_Y}$ and $z_D = \hat{\beta}_D / \sigma_{\hat{\beta}_D}$ and found strong correlation ($r^2 > 0.9$ across the scenarios considered), but with z_D being systematically smaller than z_Y, with on average $z_D = 0.73\, z_Y$, corresponding approximately to a two-fold decrease in sample size for the dichotomized outcome. We used this *ad hoc* correction factors when plotting the obesity GENESIS results (**Fig. S8**).

Specificity of the genetic model for Alzheimer disease

Genetic variants within the Apolipoprotein E (ApoE) gene encode for three haplotypes, ε2, ε3 and ε4 that are critical determinants of Alzheimer disease risk[11]. Those variants with large effect deviate from the standard polygenic model and are commonly assessed separately from the effect of other variants. The causal APOE variants were available in the three largest Alzheimer disease GWAS (Lambert et al. 2013, Kunkle et al. 2019 and Jansen et al. 2019), but absent from the smallest one (Li et al. 2008), thus potentially impacting the comparison of the predictive performance. Moreover, the LDpred2[12] approach used to derive the polygenic risk score (PRS) can be sensitive to such deviation of the polygenic model. To address this possible limitation,

in complement to the standard pipeline, we derived three additional predictive models: a PRS excluding the APOE region (PRS.noAPOE), a risk score based on APOE ε2 and ε4 allele genotypes (rs429358 and rs7412) with effect estimates pulled from Kunkle et al[13] (the largest GWAS for AD status) (PRS.APOE), and a PRS including PRS.noAPOE and PRS.APOE as two distinct components. The predictive performances from these alternative PRSs are presented in **Figure S3**. Also, note that when using the PRS excluding APOE in **Figure 3**, we used the twin heritability estimates after subtracting the contribution of APOE variants, which has been reported to be approximately 9% of the total phenotypic variance[14].

Another challenge in the analysis of Alzheimer disease is the use of the so-called AD-by-proxy instead of the AD status in the largest GWAS (Jansen et al[2]). AD-by-proxy is a score determined by the Alzheimer status of the parents' participants. It has been proposed to virtually increase the available sample size in AD GWAS and therefore increase statistical power. In our estimation of the AUC in the FinnGen cohort, we use the true AD status. This might potentially explain the limited increase in AUC in that cohort when using the Jansen et al[2] PRS. Conversely, in the estimation of the AUC in the non-European UKB participants, we used the AD-by-proxy as the primary outcome because of a limited sample size for the true AD status (N=961 for AD-by-proxy cases, and N=65 for AD case). For that cohort, the AUC show a substantial increase for the Jansen et al PRS, likely due to the outcome correspondence. While a full investigation of the impact of using AD-b-proxy is out of the scope of this study, we derived the predictive power of the APOE variants (PRS.APOE) in the six non-European UK Biobank populations as a qualitative marker of this proxy phenotype. We obtained AUC=0.65 (SD=0.58) and AUC=0.54 (SD=0.52), for the AD status and the AD-by-proxy status, respectively. This is in line with the expectation that a loose disease definition can impact the predictive power of genetic risk score.

Parameters estimation from GWAS using existing tools

We used multiple tools to estimate disease genetic parameters from GWAS summary statistics. We applied GENESIS[15], SBayesS[16], sumHer[17], LDSC[18], and MiXeR[19,20] to all GWAS from **Table S2** except for Saxena et al 2007 and Li et al 2008 because of modest sample size (N_{eff}=2,931 and 1,489, respectively) (**Table S6** and **Figs. S8-S10**). All methods estimate heritability. GENESIS, SBayesS, and MiXeR additionally estimate the number of causal variant and the polygenicity. SBayesS and sumHer also provide estimates of α, the MAF-effect size relationship parameter. GENESIS output was also used to derive the expected trend of the AUC as a function of sample size (**Fig. S8**). We parametrized the method following the available tutorials and direct recommendations from the authors. Note that those methods sometimes refer to parameters that have different definition or labels (e.g. α is sometimes referred to as S, effective sample size is defined differently across methods, the output heritability scale is heterogeneous, etc). When possible, we harmonized the output estimates to allow for a direct comparison across methods. Below is a brief description of the overall analysis pipeline and specific parametrization of each method.

All analyses were conducted using GWAS variants overlapping with a reference set of 1,054,330 variants from HapMap3[21]. Most of these methods assume the original GWAS have not been corrected for chi-squared inflation using genomic control (GC)[22]. We carefully read each GWAS paper to assess whether GC correction was applied. The correction strategy across GWASs was heterogeneous (**Table S2**), sometimes applied at the study level (by each study, before the meta-analysis), or at the meta-analysis stage, at both, or neither. The information was also sometimes missing, or partial (a correction was reported, but the actual correction factor was not provided). We identified three studies were the inflation factor λ_{GC} use for correction was fairly large (Nikpay et al. 2015, Speliotes et al. 2010, and Locke et al. 2015) and we back-corrected the GWAS results before the analysis.

<u>GENESIS</u> produces estimates of the SNP heritability, the number of causal variants, and allows to generate expected AUC as a function of the sample size. Following recommendations from the authors, we ran a three-component mixture model, which assumes that the effect sizes for susceptibility variants can be described by two distinct normal distributions (small and large effects) and a third component corresponding to non-associated variants. We use as input the effective sample size derived as $N_{eff} = N_{cases} \times N_{controls} / (N_{cases} + N_{controls})$, but report the results using the alternative derivation $N_{eff} = 4\, N_{cases} \times N_{controls} /$

$(N_{cases} + N_{controls})$. The heritability estimated in GENESIS is on the log-odds scale and was transformed to the liability scale using the formula $h_l^2 = h_{log}^2 K^2(1-K)^2/z^2$, where K is the disease prevalence in the population and z the height of the standard normal probability density function at the liability threshold[10].

SBayesS produces estimates of the number of causal variants, the SNP heritability and α. We used the shrunk LD matrices proposed along the software. It requires the frequency of the coded allele, which was not available for several GWAS. Instead, we used frequencies derived from participants of European ancestry in the 1KG reference panel[9]. For all analyses we used the options *--exclude-mhc* to remove the MHC region from the derivation, and *--impute-n* that re-derive the sample size, as the per-variant sample size is likely heterogeneous for many of the GWAS analysed. This option also filters out variants that have sample size 3 standard deviation away from the expected. For the sample size, we used the total sample: $N = N_{cases} + N_{controls}$. Heritability on the liability scale was derived in a second step using the formula[10]: $h_l^2 = h_{obs}^2 \left(K(1-K)\right)^2/(P(1-P)z^2)$, where K and P are the population and in-sample prevalence, respectively.

MiXeR[19,20] produces estimates of the fraction and number of causal SNPs and the SNP heritability, based on GWAS summary statistics and assuming a Gaussian mixture model. MiXeR takes into account SNP heterozygosity, LD structure, and residual inflation of z-scores due to variance distortion (which can arise from cryptic relatedness in the sample). We used the default reference panel, the 1000 Genomes Phase3 data and the MHC region was excluded from the analysis. As recommended by the authors, we used the standard definition of the effective sample size: $N_{eff} = 4\, N_{cases} \times N_{controls} /(N_{cases} + N_{controls})$. Heritability on the liability scale was derived in a second step using the formula[10]: $h_l^2 = h_{obs}^2 \left(K(1-K)\right)^2/(P(1-P)z^2)$, where K and P are the population and in-sample prevalence, respectively.

LDSC regression[18] was used to derive estimates of SNP heritability. Here we used the original model proposed in 2015 by Bulik-Sulivan et al[18], which has been widely used in the field. Heritability is derived from a regression between the chi-squared for association and the LDscore, a metric that quantifies the strength of the correlation between each variant and its neighbours. The standard model implicitly assumes an infinitesimal model with an equal per-variant contribution to the heritability. For the sample size, we used the total sample: $N = N_{cases} + N_{controls}$. Heritability on the liability scale was derived in a second step using the formula[10]: $h_l^2 = h_{obs}^2 \left(K(1-K)\right)^2/(P(1-P)z^2)$, where K and P are the population and in-sample prevalence, respectively.

sumHer[17] is an extension of the LDAK model[23] for summary statistics. As compared to LDSC, sumHer uses a model were per-SNP heritability varies with both linkage disequilibrium (LD) and minor allele frequency (MAF). We used the options *--cutoff* 0.01 to remove variants that explain more than 1% of phenotypic variance, and specified the in-sample and population prevalence with *--prevalence* and *--ascertainment*, so that the heritability is directly derived on the liability scale. For the sample size we used $N = N_{cases} + N_{controls}$. For the estimation of α we used the solution proposed within sumHer that consists in testing a range of α, and estimating the best fit (i.e. the highest log-Likelihood) using the *--find-gaussian* option.

Simulations to study the expected convergence of PRS

To characterize the convergence of PRS prediction as a function of the disease parameters and sample size, we simulated series of matched case/control (i.e. $n = n_{case} = n_{control}$) replicates varying the disease prevalence (K = [0.01; 0.25], the disease heritability (h^2 = [0.2; 0.7], the number of causal genetic variants (M = [100, 1000, 5000, 10,000], and the sample size used in the GWAS (n = [250; 2,500; 25,000; 250,000]) (**Fig. S7**). Independent causal single nucleotide polymorphisms (SNP) were generated under a binomial distribution with minor allele frequency randomly drawn in [0.05, 0.5]. The disease status was generated under a liability threshold model. The effect of the SNPs on the liability were drawn from a gaussian distribution $\beta \sim \mathcal{N}(0, h^2/M)$, and the threshold defined as $\mathrm{T} = \Phi^{-1}(1-k)$ where Φ is the cumulative distribution function of the normal distribution. Each replicate was split in two subsets. The first subset was used to estimate genetic effect $\hat{\beta}$ for each of the $G_{i=1\ldots M}$ variants using univariate logistic regression. A PRS was then computed in the second subset as: $PRS = \sum_{i=1\ldots M} \hat{\beta}_i\, G_i$. The predictive performances of the PRS for each

model was derived as the average over 10 simulations of the Area under the ROC (AUC) curve in this second subset.

Estimation of alpha for common diseases using UK Biobank individual-level data

We derived the α parameter for the six outcomes using individual-level genetic and phenotypic data from the UK Biobank using the approach described by Schoech et al[24] (**Fig. S15-S16**). In brief, we derived $\mathbf{A}_\alpha$, the genetic correlation matrices (GRM) conditional on a pre-specific α values in [-1, 0], and defined as $\mathbf{A}_\alpha = \mathbf{X}\mathbf{D}_\alpha\mathbf{X}^t$, where $\mathbf{X}$ is the genotype matrix and $\mathbf{D}_\alpha$ is a diagonal matrix with element $D_{ii} = [2p_i(1-p_i)]^\alpha$. For each outcome Y, profile likelihoods $L(\alpha)$ of the multivariate normal model $Y \sim \mathcal{N}(0, \mathbf{A}_\alpha\sigma^2_{g,\alpha} + \mathbf{I}\sigma^2_\varepsilon)$, where σ^2_ε is the residual variance, were derived for a range of α value using the GCTA software[25]. The final $\hat{\alpha}$ estimate was derived as $\hat{\alpha} = argmax(L(\alpha))$. Estimation was done using up to 261,028 unrelated self-reported "white British" participants with genetic data for 17,211,988 variants with minor allele frequency (MAF) equal or larger than 0.01%, and *info_score* larger than 0.8.

In practice, the estimation of this parameter from individual-level data poses severe computational challenges, and the GRM-MAF-LD package proposed by Schoech et al[24] was not runnable in these data. We followed the same procedure, but re-implemented each step and optimized the parameters for computational purposes. For the derivation of $\mathbf{A}_\alpha$, we modified the GRM function from PLINK 2.0[26], to include the α weighting. Due to memory constraints, GCTA could not be run using a GRM of several hundred thousand individuals at once. To address this limitation, we divided the dataset into eight equally sized random sub-samples, and estimated $L(\alpha)_s$ for each subsample s using GCTA. The overall $L(\alpha)$ was then derived as the sum of the resulting log likelihoods: $L(\alpha) = \sum_{s=1\dots8} L(\alpha)_s$. We considered a range of α values in [-1.3 ; 0], with 0.1 steps. Data points were then interpolated using a Locally Weighted Least Squares Regression as implemented in the R *loess()* function to obtain the final estimate of $\hat{\alpha}$. We validated the procedure using real genotype data from the UK Biobank cohort and simulated phenotypes (**Fig. S16a**). We also attempted to estimate alpha across the MAF strata. However, this estimation was unreliable, displaying slow convergence because of the limited variance within each MAF bin (**Fig. S16b**).

Attenuated alpha model

The alpha model is defined as $\beta_i | p_i \sim \mathcal{N}(0, \sigma^2_{g,\alpha} \cdot [2p_i(1-p_i)]^\alpha)$, where p_i is the minor allele frequency of variant i, and $\sigma^2_{g,\alpha}$ is a constant constraining the outcome heritability. For small value of alpha, it implies the per-allele effect of rare variants can be several orders of magnitude larger than for common variants (**Fig. S12a**). As noted in Schoech et al[24], the fit of the alpha model in evolutionary forward simulations is expected to hold above a fairly low MAF threshold (MAF>0.6% in the reported example), but might overestimate the effect of variants with MAF below that threshold. Let's denote θ the scaling factor of effect size coefficient, so that $\theta_i = [2p_i(1-p_i)]^\alpha$. To account for possible deviations of the alpha model, we considered attenuated effect size θ_i^* for very rare variants. We defined the attenuation using an inverse logit function with a tipping point at the aforementioned MAF threshold T=0.6%: $\pi = 1/[1 + exp(\log(MAF) - \log(T))]$. The attenuation was applied recursively to the beta coefficient ordered by decreasing MAF: $\theta_i^* = \theta_{i-1}^* + (\theta_i - \theta_{i-1})(1 - \pi w)$, where w is a weight in [0, 1] to set the strength of the attenuation. **Figure S12b** illustrates the effect of the proposed attenuation on the MAF-effect size relationship for $\alpha = -0.3$. We varied the weight w over [0.1, 0.3, 0.5, 0.7, 0.85, 0.95, 0.99] and repeated the experiment from **Figure 3a**, deriving the expected proportion of heritability captured across this range of attenuated alpha model using either r^2, the squared correlation between the sequence and imputed variants (**Fig. S13b**), or ρ^2, the squared correlation between the untyped variants j and the genotyped ones (**Fig. S13a**). When comparing those attenuated models against the baseline alpha model in real data, we did not find any evidence for an increased fit (**Fig. S15**).

Supplementary Figures

Figure S1. European and FinnGen GWAS

Beta coefficients at top associated independent variants from FinnGen GWASs and European ancestry GWASs for six outcomes: obesity (a), breast cancer (b), asthma (c), type 2 diabetes (d), Alzheimer disease (e), and coronary artery disease (f). Across all variants, squared-correlation equal 0.43 (obesity), 0.56 (breast cancer), 0.50 (asthma), 0.67 (type 2 diabetes), 0.55 (Alzheimer disease), and 0.66 (coronary artery disease). Note that for obesity the European GWAS results were extracted from a BMI GWAS, explaining the scaling difference in regression coefficient. For Alzheimer disease (AD), the European GWAS was derived using AD-by-proxy, inducing a larger variance in the outcome, and again difference in the scaling of the regression coefficient.

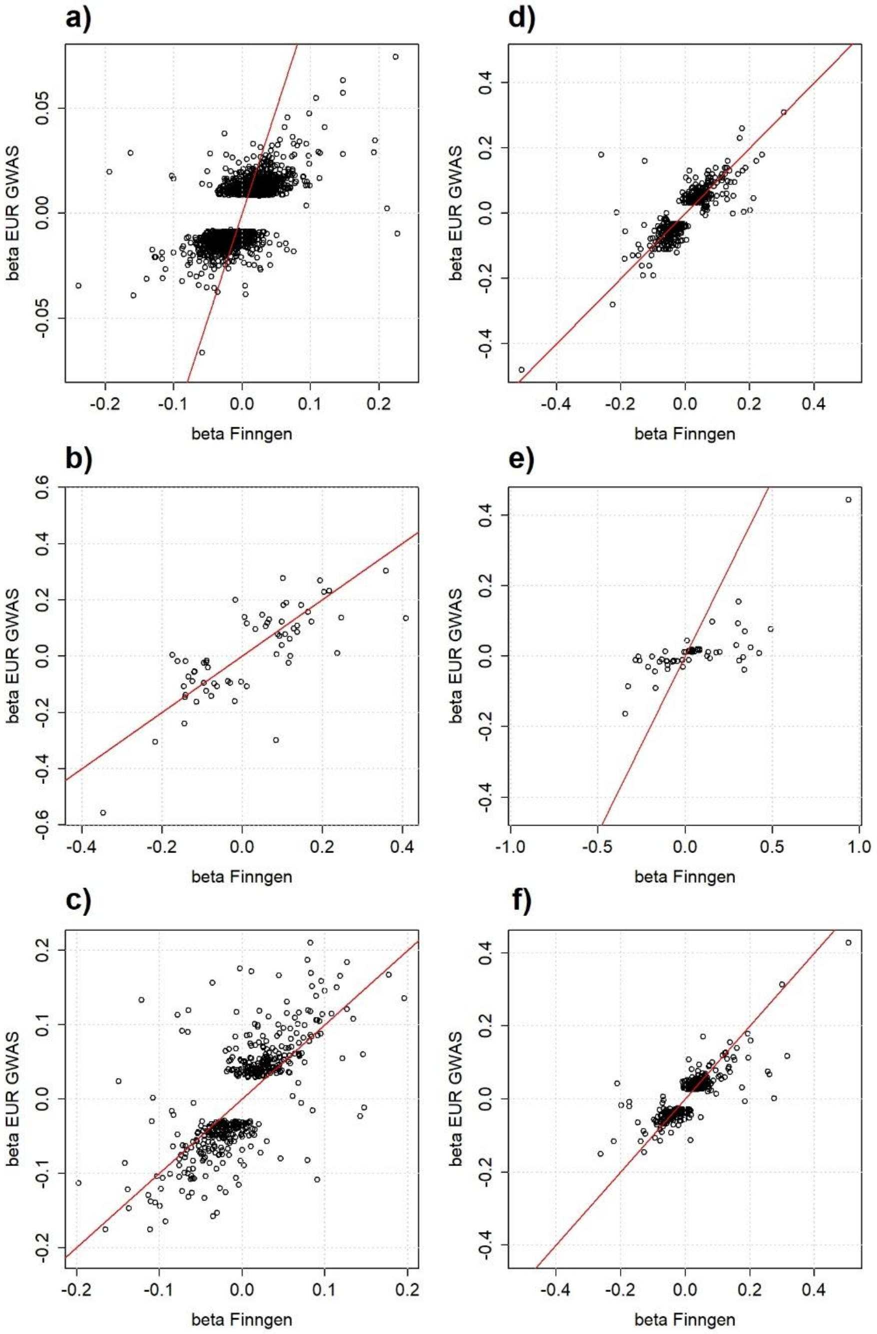

Figure S2. AUC as a function of sample size for 6 UK Biobank populations

AUC as a function of the effective sample size for each of the 6 ancestries analysed in the *UK Biobank* (Ashkenazi, Iranian, Indian, Chinese, Caribbean, and Nigerian). Grey areas correspond to the 95% confidence interval. AUC was derived for 6 outcomes: coronary artery disease (CAD), type 2 diabetes (T2D), breast cancer (BRCA), Alzheimer disease (AD), asthma, and obesity. Obesity was predicted using BMI GWASs. For AD, the outcome predicted was AD-by-proxy because of a limited number of AD cases within each ancestry. The PRS for this outcome was derived after excluding the APOE region.

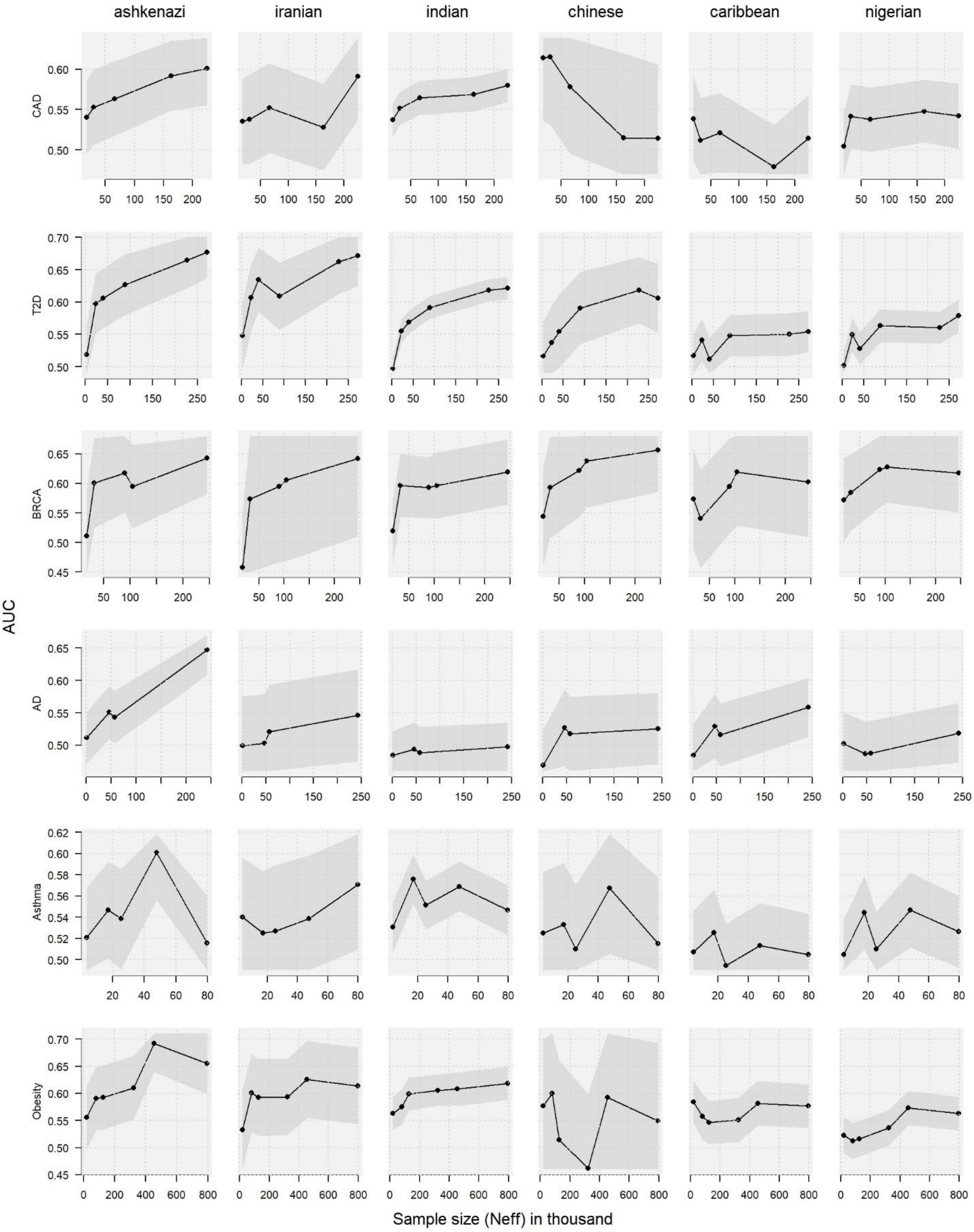

Figure S3. Variability in Alzheimer prediction conditional on APOE

For Alzheimer disease we considered three alternative genetic models conditional on the APOE region: i) a PRS derived after excluding the APOE region ("PRS excluding APOE", plain line), which was used as the main analysis, ii) a PRS derived using the same pipeline as for other diseases treating the APOE region as the rest of the genome ("*PRS including APOE region*", dash line), and iii) the combination of a PRS derived excluding the APOE region and score for the ε_1 and ε_2 variants as a distinct predictors ("*PRS and APOE treated separately and merged*", dotted line). The panels present the results of each of the three genetic scores in the FinnGen participants, and the non-European UK biobank participants per population and after a meta-analysis ("*UKB Combined*").

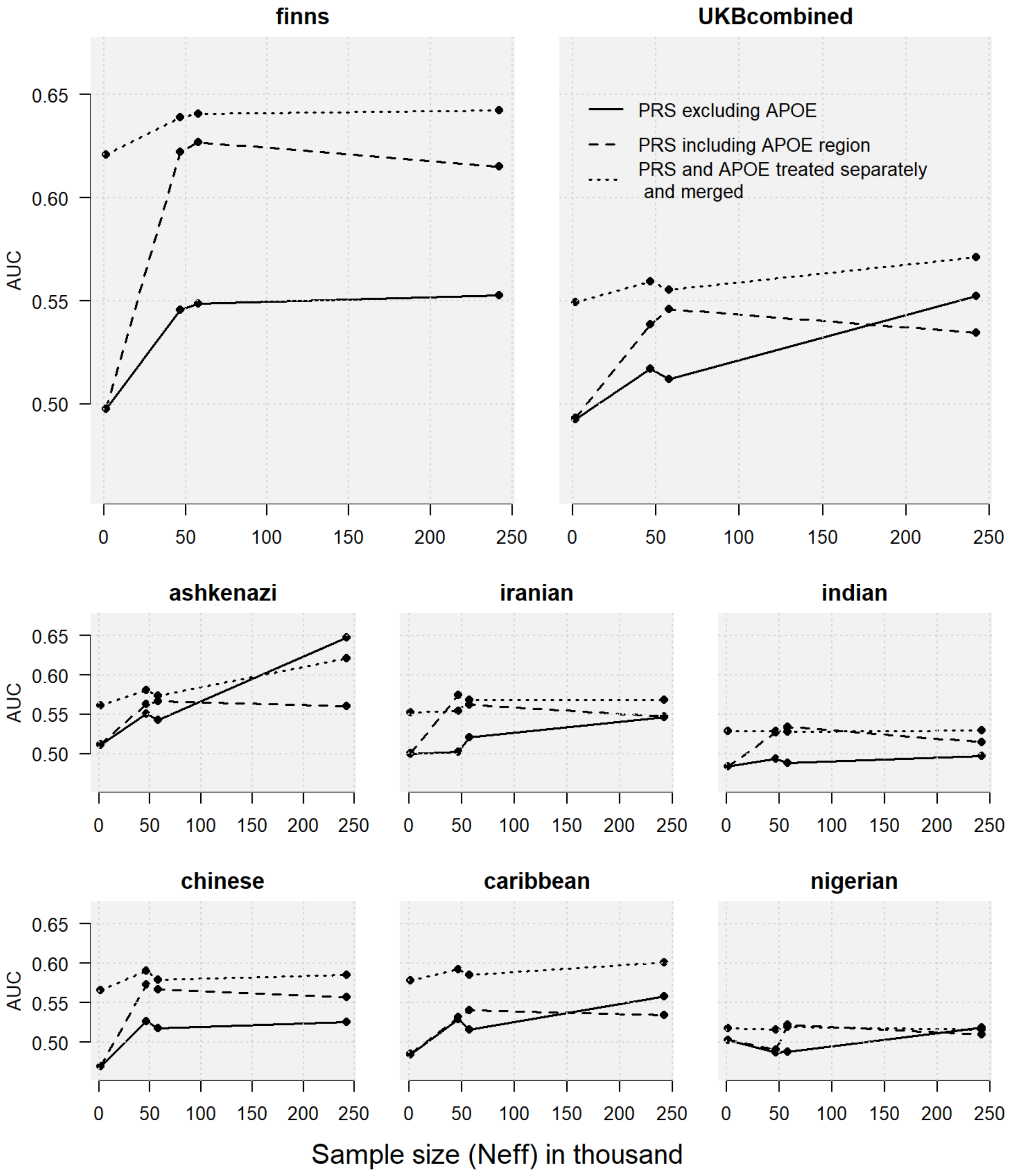

Figure S4. AUC based on PRS derived from alternative methods

AUC for the six outcomes derived using the same pipeline as in Figure 1b and alternative polygenic risk scores (PRS). PRS were trained from the 35 genome-wide association study (GWAS) using LDpred2, SBayesR, and a clumping + thresholding using a P-value threshold of 0.05, 10^{-4} and 10^{-8}. The PRS were tested in individual-level data from the FinnGen cohort. The AUCs are plotted as a function of the effective sample size of the corresponding GWAS.

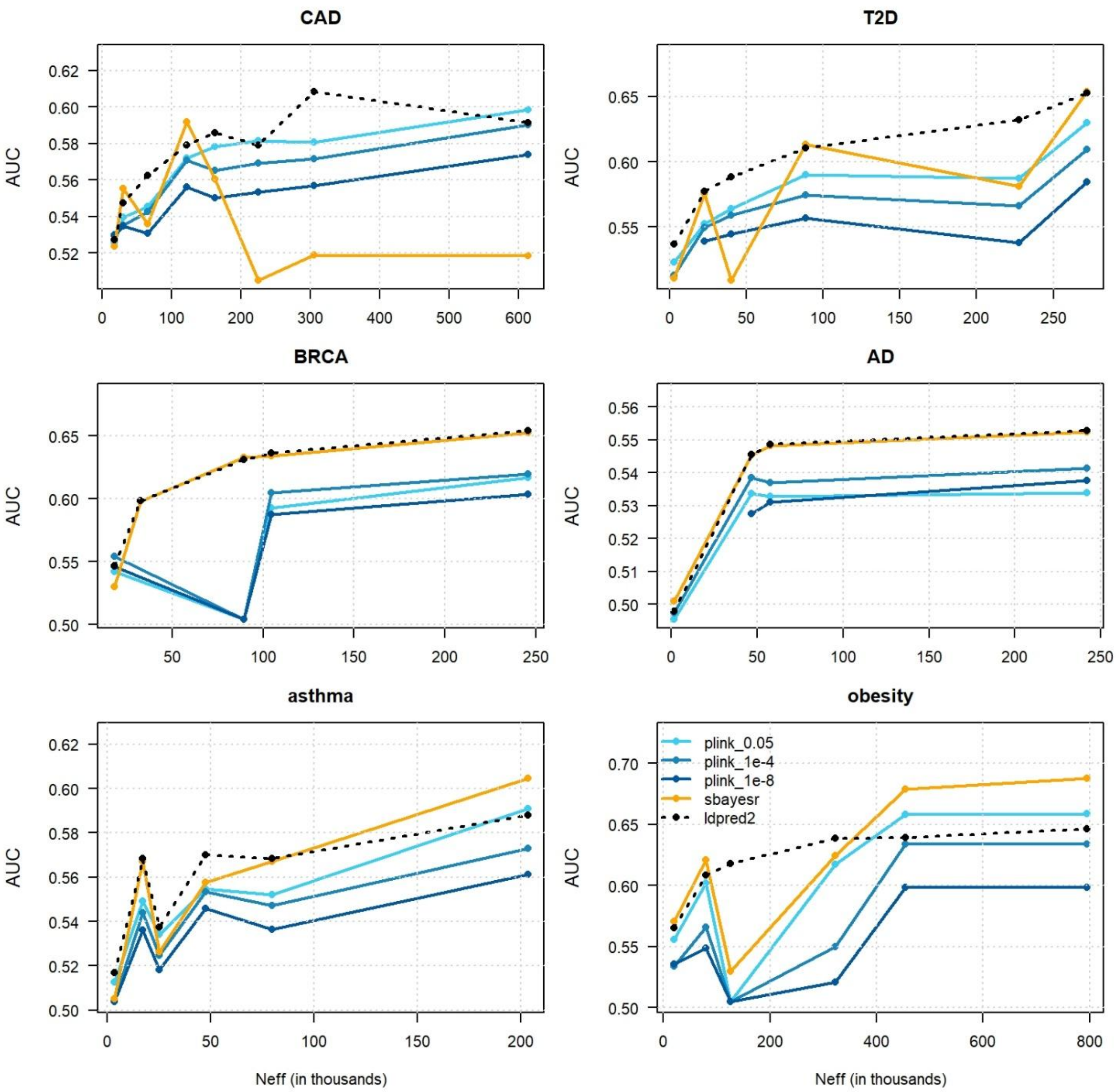

Figure S5. Prediction accuracy measured by alternative metrics

Prediction accuracy for the six outcomes measured by AUC, R2, and odds ratios (OR) of the disease comparing the top 5% and top 1% PRS stratum and the general population. Polygenic risk scores were trained from 35 genome-wide association study (GWAS) using LDpred2 and tested in individual-level data from the FinnGen cohort. All accuracy metrics are plotted as a function of the effective sample size of the corresponding GWAS. To facilitate the comparison of the trend across metrics, within each panel, we aligned the scale of each metric in order to match the maximum and minimum values. The three corresponding ordinates are presented for AUC (black), R2 (yellow), and OR (blue).

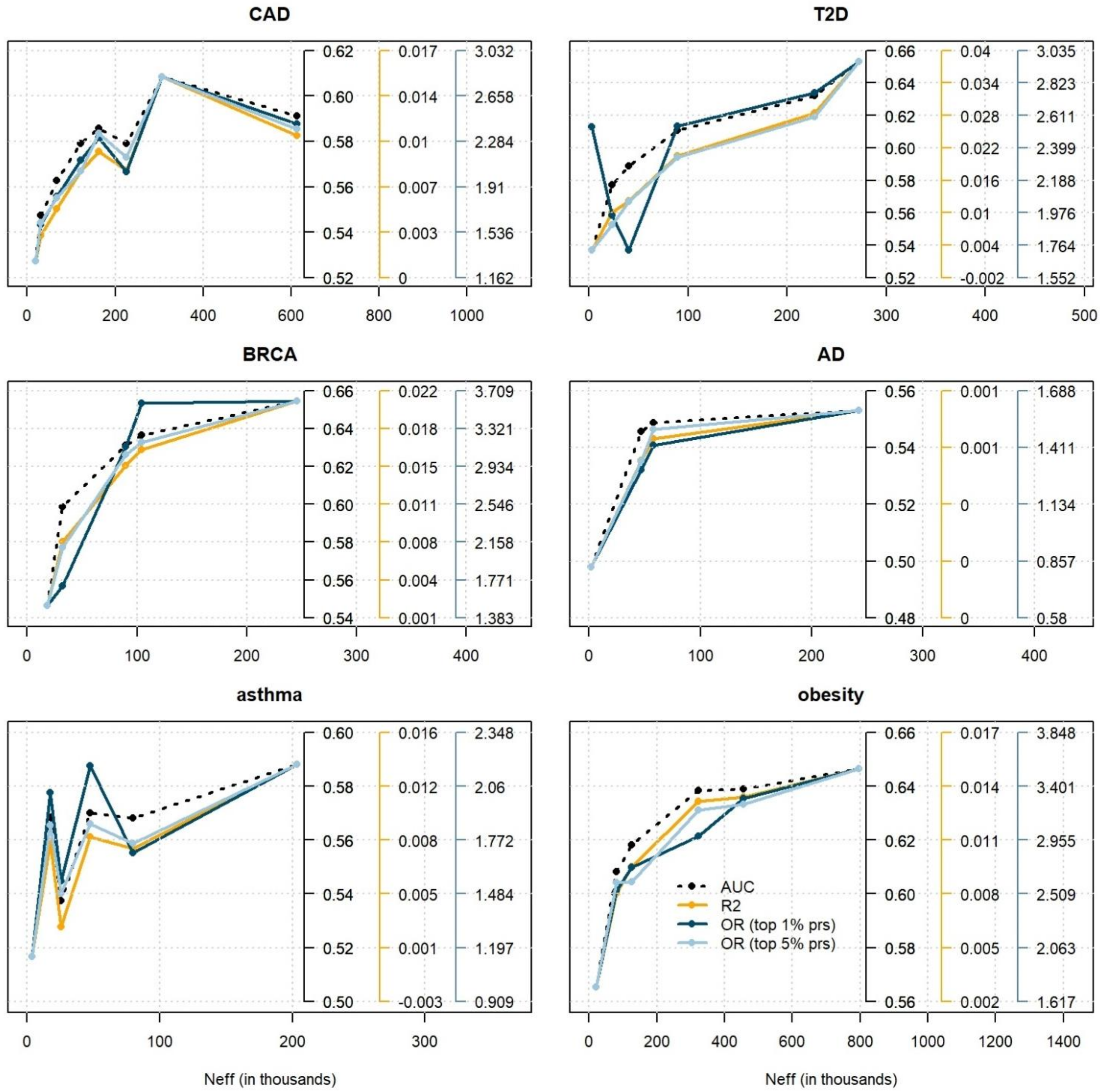

Figure S6. Sample overlap and overfitting

We investigated the impact of sample overlap in the prediction accuracy of PRS, as measured by the AUC, using data from the UK Biobank and BMI. We used a training set of 200,000 unrelated participants of European ancestry to build a polygenic risk score for BMI using LDpred2. We applied this PRS to predict obesity in 10,000 unrelated participants of European ancestry, while sampling the test set either independently from the training set, or considering a partial overlap with the training (20%, 40%, 60%, 80 and 100%). The left panel presents the AUC derived in the test set as a function of the sample overlap. The grey area represents the 95% confidence interval of the AUC. The right panel illustrates the sample overlap.

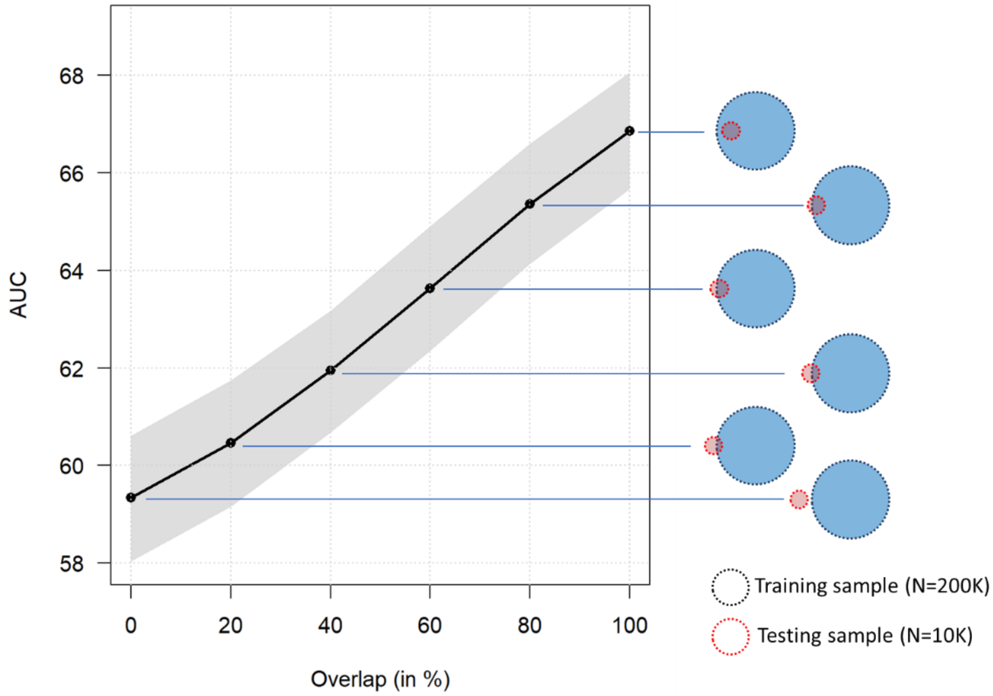

Figure S7. AUC convergence using simulated causal variants

Average predictive power as measured by the Area under the ROC (AUC) in simulated matched case/control replicates. Disease status was drawn under a liability threshold model using 100 to 10K independent causal variants. For each replicate, the effect of causal variants was estimated using univariate logistic regression using an increasing sample size (n = [250; 2,500; 25,000; 250,000]). A PRS was then computed in an independent subset using those estimates and its performance derived as the average AUC over 10 simulations. Results across low and high heritability (h_A^2) and disease prevalence (K) are presented in panel a) (h_A^2=0.2, K=0.25), b) (h_A^2=0.2, K=0.01), c) (h_A^2=0.7, K=0.25), and d) (h_A^2=0.7, K=0.01). The black dash line represents the expected maximum achievable AUC derived based on heritability and prevalence.

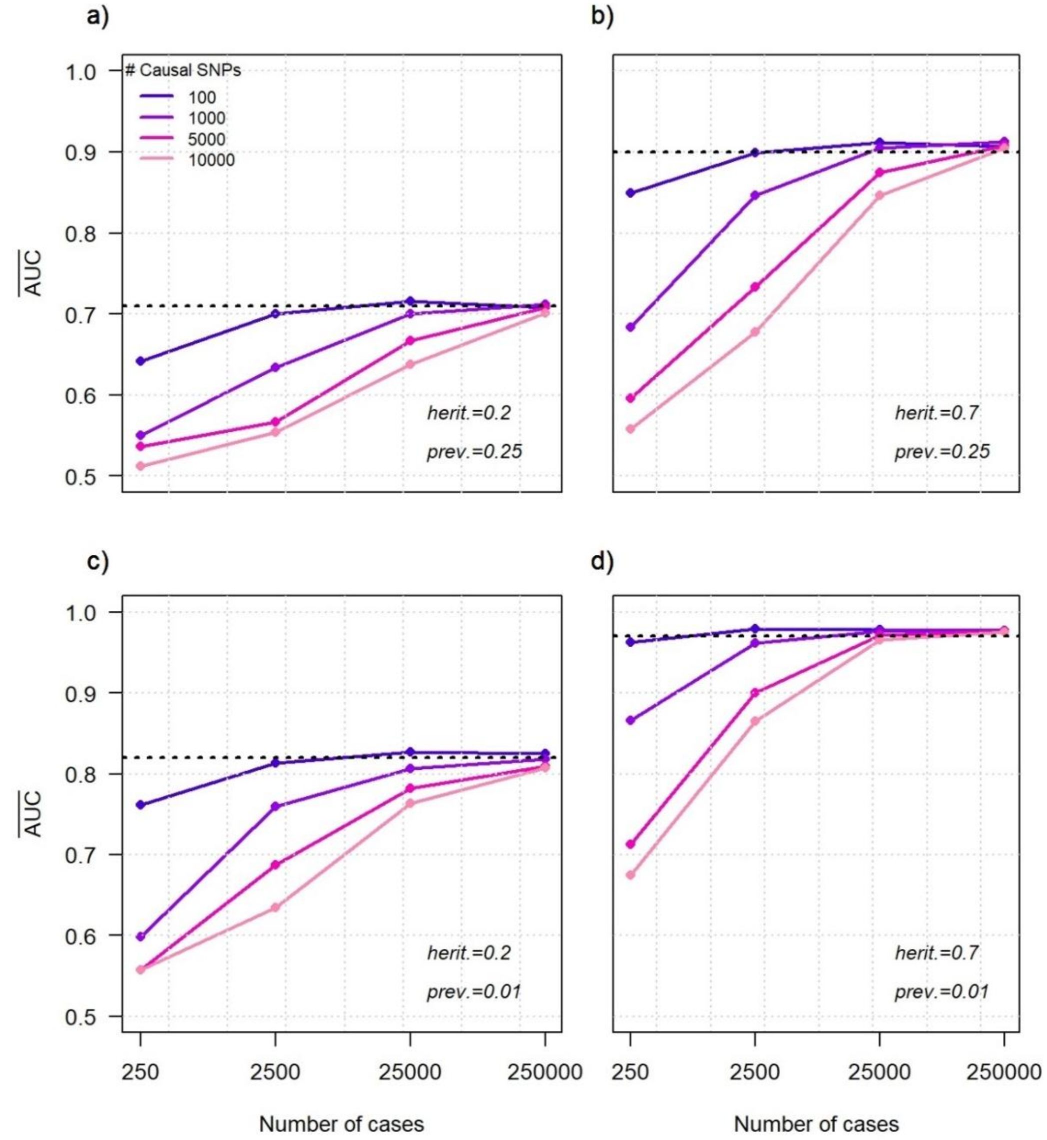

Figure S8. AUC increase, heritability and number of causal variants derived from GENESIS

Predicted AUC as a function of sample size derived from the GENESIS package assuming a 3-components genetic model, where variants are classified as either non-causal, causal with low effect, or causal with large effect. GENESIS was applied to all GWAS data form each of the six outcomes, coronary artery disease, breast cancer, type 2 diabetes, Alzheimer disease, asthma, and obesity. Note that for obesity, we used body mass index (BMI) GWAS and applied an *ad hoc* correction of the sample size, expected to match power of a binarized version of BMI. AUC derived in FinnGen and the UK Biobank are indicated in black and red, respectively(a). The predicted AUC are based on estimates of heritability (b) and the number of causal variants (c) estimated within GENESIS. Error bars indicate the 95% confidence interval. The shape of the predicted AUC as a function of sample size is reasonably close to the ones observed in FinnGen and the UK Biobank**,** however, the increase rate and expected maximum AUC varied substantially conditional on the input GWAS used.

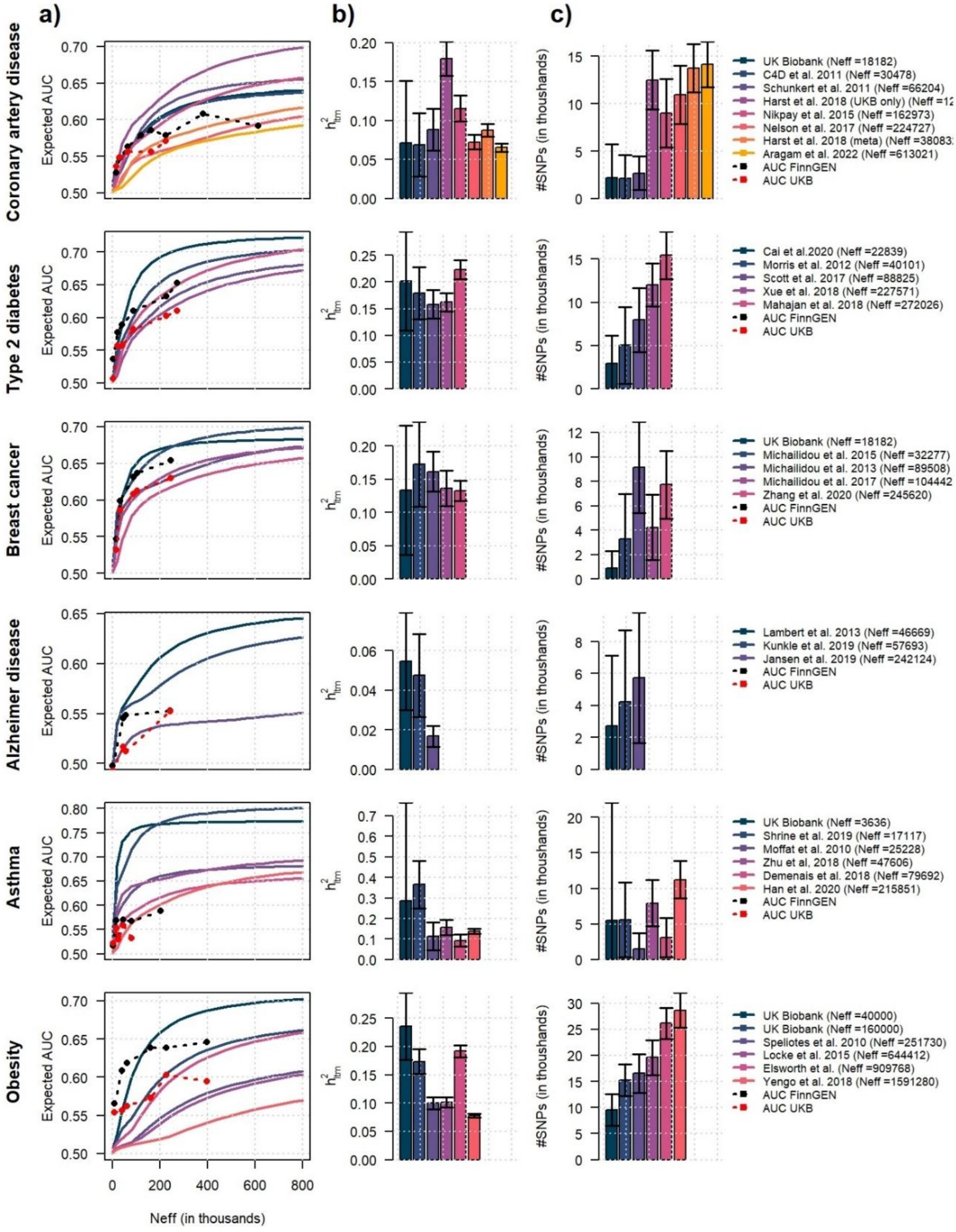

Figure S9. SBayesS disease parameters estimates across the six outcomes and GWAS

Estimates and 95% confidence interval of polygenicity (the proportion of causal variants), heritability and alpha derived using SBayesS across 31 GWAS summary statistics from six outcomes: coronary artery disease (CAD), breast cancer (BRCA), type 2 diabetes (T2D), Alzheimer disease (AD), asthma (AS), and body mass index (BMI). We only include GWAS with an effective sample size larger than 5,000. All analysis were conducted using a subset of approximately 1M hapmap3 variants.

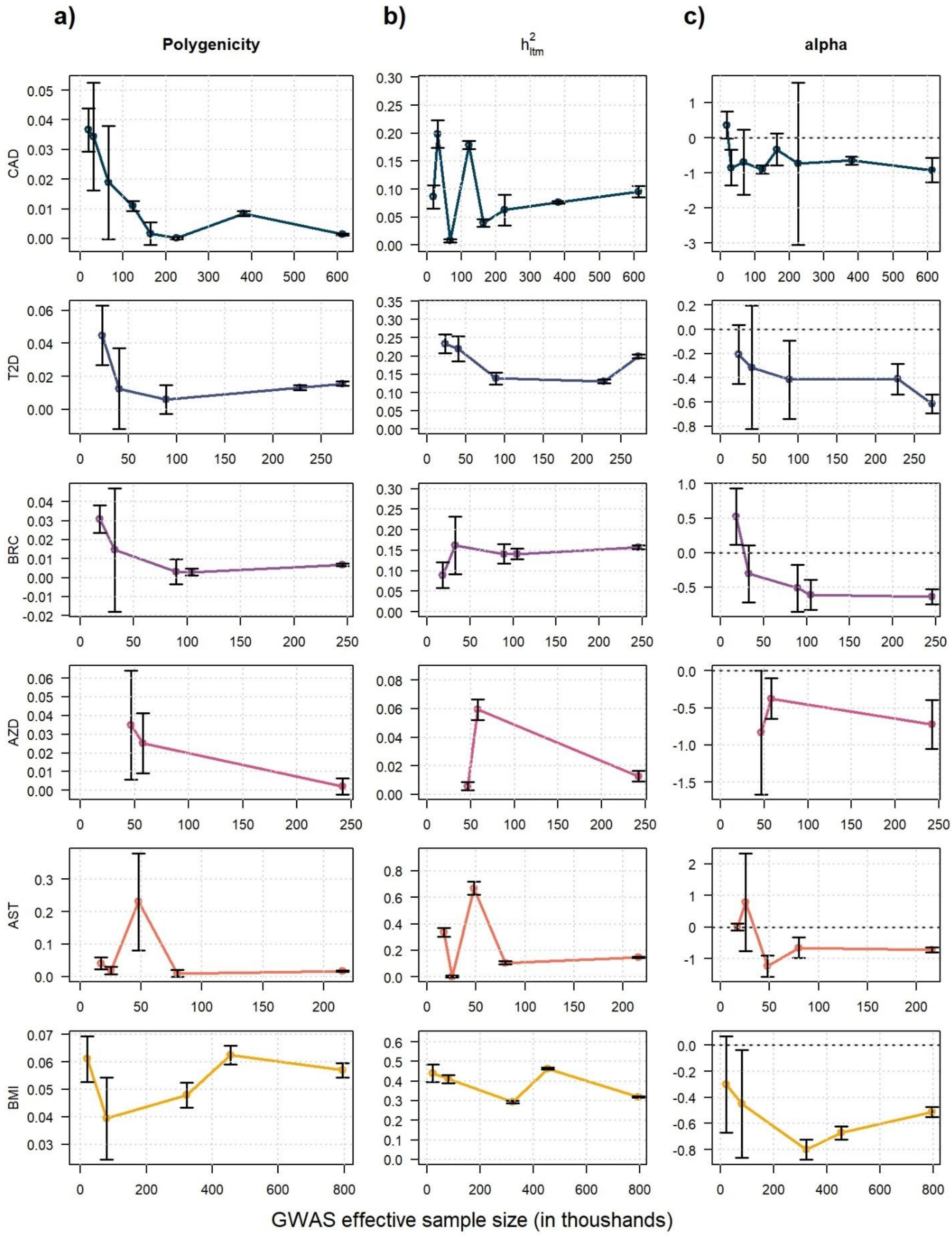

Figure S10. Assessing the impact of GWAS heterogeneity on disease parameter estimation and AUC

We conducted GWAS for body mass index (BMI) in random subsamples of the UK biobank with sample size N increasing from 20K to 300K. For each GWAS, we applied SBayesS to estimate polygenicity (the percentage of causal variants), alpha, and heritability under the liability threshold model. For each resulting GWAS, we applied LDpred2 to derived PRS following the same pipeline as for Figure 1, and applied these PRSs to derive the AUC in a holdout test dataset from the UK Biobank not used in the GWAS. We repeated the analysis five times with a different holdout subset for each analysis. Panel a), b) and c) present estimates of polygenicity, alpha and heritability across the five experiments as a function of sample size. Panel d) presents the average AUC derived in the UK Biobank holdout (blue). Results AUC from Figure 1b (obesity), derived in FinnGen ("Finns external") and in non-European participants from UKB ("UKB external") using PRS from external GWAS are presented in red and black, respectively.

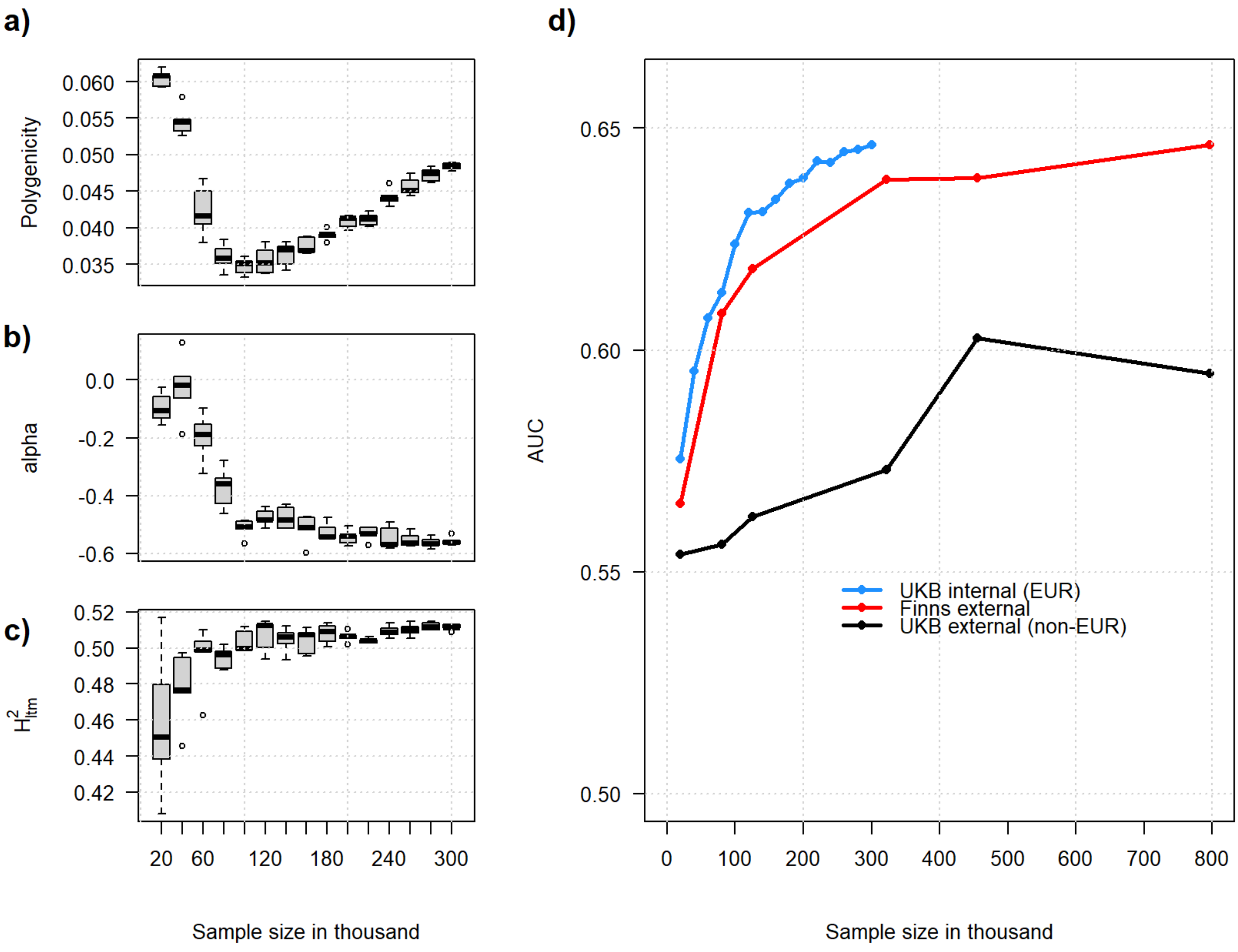

Figure S11. AUC across CAD phenotypes

AUC for seven CAD phenotypes derived using the CAD PRS in FinnGens: myocardial infarction (I9_MI), myocardial infarction, strict (I9_MI_STRICT), coronary revascularization (I9_REVASC), ischemic heart diseases (I9_ISCHHEART), coronary atherosclerosis (I9_CORATHER), coronary angioplasty (I9_ANGIO), angina pectoris (I9_ANGINA). Right panel presents the number of cases in the FinnGen cohort for each phenotype

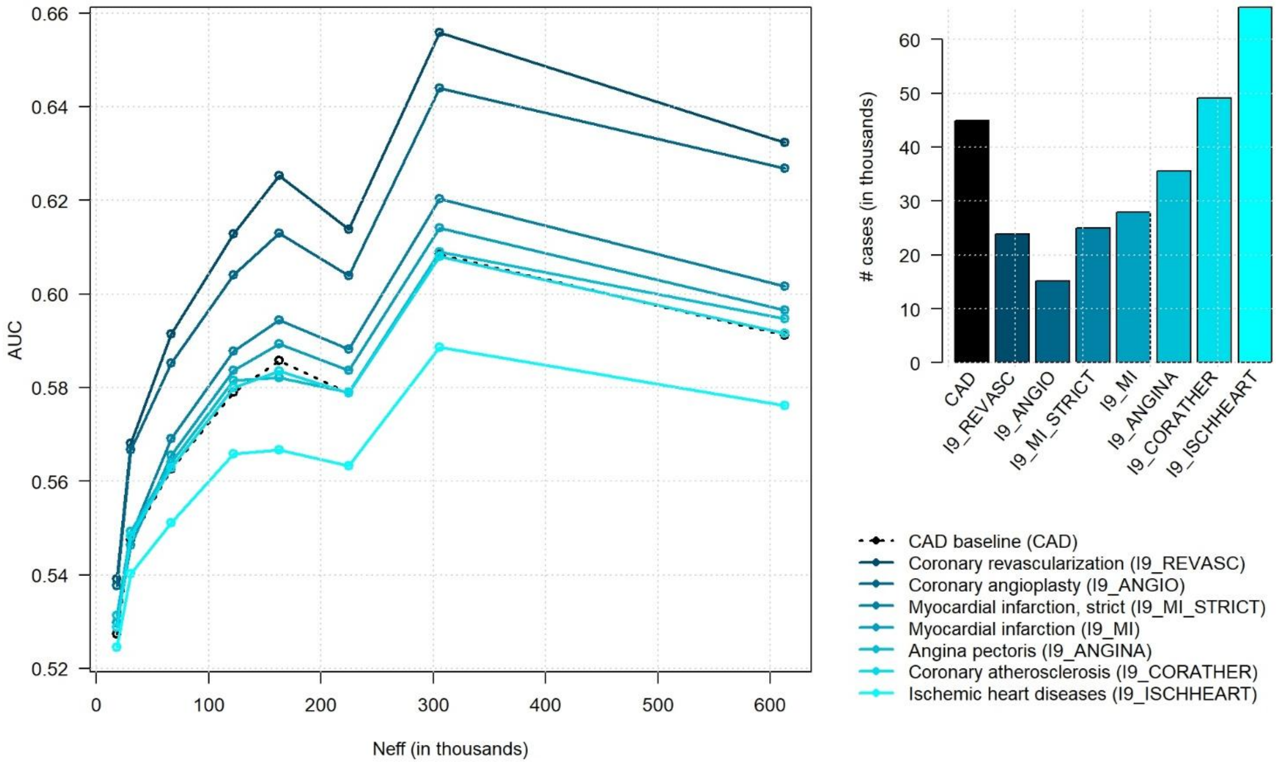

Figure S12. Baseline and attenuated alpha models and proportion of heritability captured

Panel a) shows the relationship between the minor allele frequency (MAF) and the per-allele effect relative to a base of 1 for a MAF of 0.5 as defined in the α model. Panel b) shows the same relationship for $a = -0.3$ after applying the proposed *ad hoc* attenuated model and using various weights. Panel c) shows the heritability captured by imputed variants and derived based on r^2_{imput}, the squared-correlation between the sequenced variants and the imputed ones, as a function of the value of alpha. Panel d) shows the heritability captured by genotyped variants and derived based on ρ^2, the variance of sequenced variants captured by genotyped variants through linkage disequilibrium as a function of the value of alpha. For both estimations we assumed the genetic effect are distributed randomly on the genome based on the α model after applying the attenuation described in panel b.

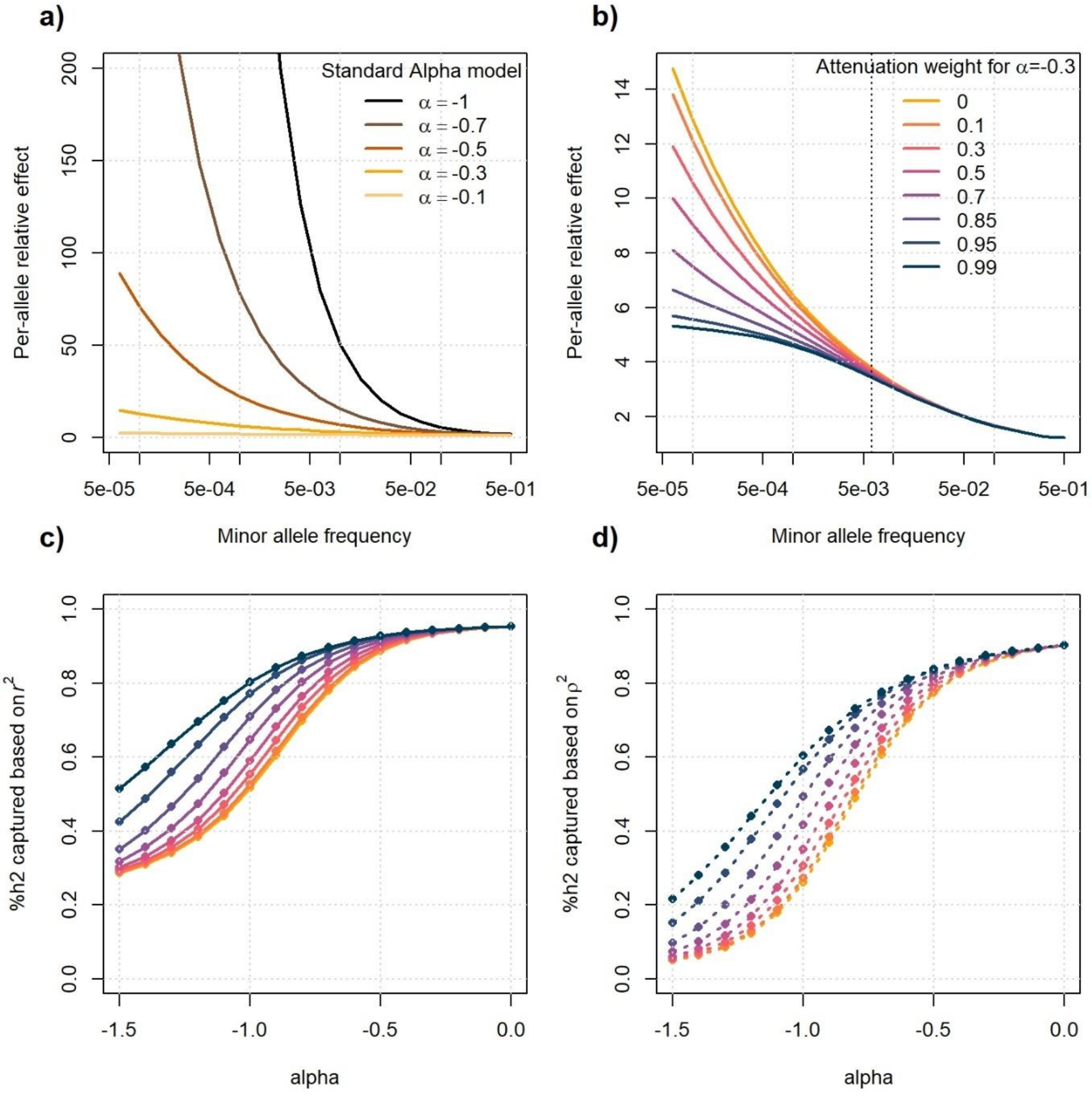

Figure S13. Variance of untyped variants captured by genotyped variants

We derived ρ_j^2 the variance of each sequenced variant j from the UK Biobank dataset captured by genotyped variants it its vicinity. We first conducted a pilot analysis used genetic data from chromosome 22 to determine the size of the windows around each variant j to be used for an optimal estimation of ρ_j^2. For this pilot, we predicted the best guess value from imputed genotypes (referred as the index variant) using genotyped variants as predictors while varying the size of the window in ±[100Kb, 3Mb] around the index. For each index we derived: i) the adjusted r-squared from a multiple regression derived in the entire sample; and ii) the squared-correlation between the predicted index and the true index using a train-test approach, were 66% (N=100K) of the sample was used as a train set and the remaining 33% (N=50K) as a test set. Panel a) shows the average ρ_j^2 over all variants of the two metrics for each windows size considered in the pilot study, highlighting an optimal at ±1.5Mb. This optimal window was used in the sequenced data to derive ρ^2. Panel b) compares the ρ^2 against r_{imput}^2, the squared-correlation between the sequenced variants and the imputed ones in the full chromosome 22 data. The density of the data points is highlighted by a gradient of colours.

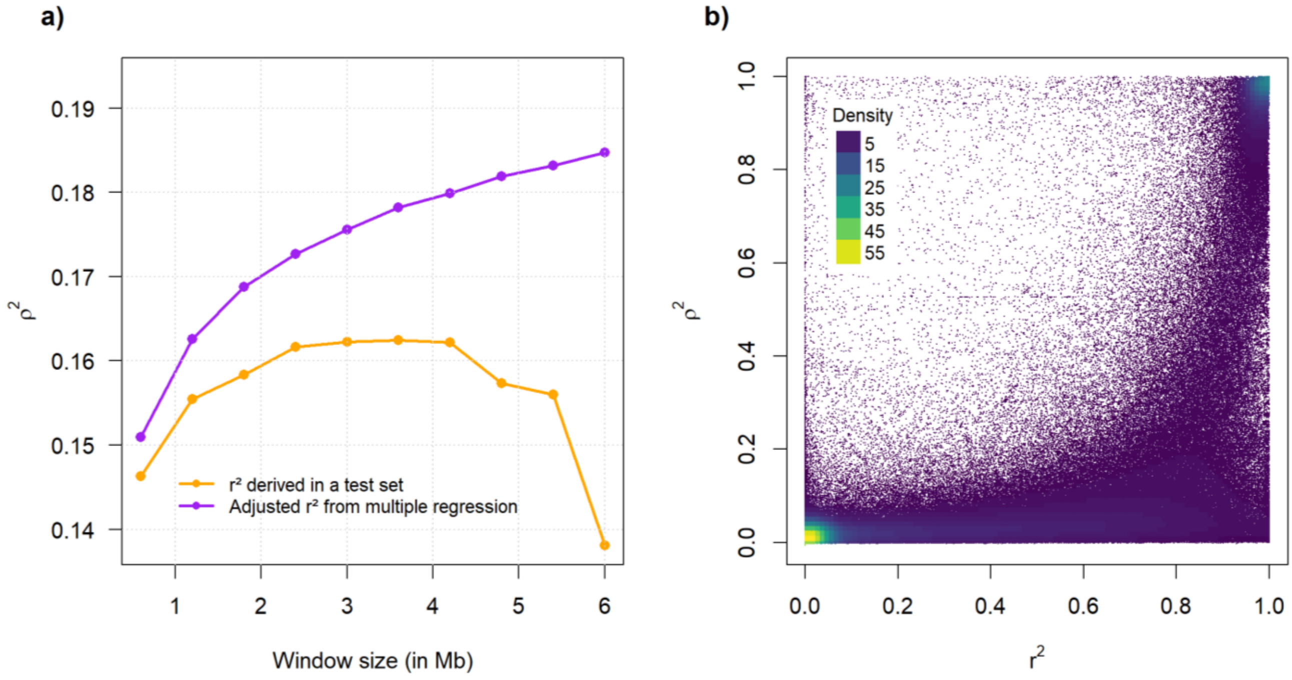

Figure S14. Variance explained by MAF bins

Expected variance explained across minor allele frequency (MAF) bins assuming the genetic effects are distributed following an α model, with α equals -1.3 (panel a)), -0.9 (panel b)), and -0.5 (panel c)). Simulations were conducted using MAF and estimated squared correlation between imputed and sequenced variants derived from 503,195 variants on chromosome 22 measured in 125,152 participants of European ancestry from the UK Biobank study. MAF was split into 20 percentiles bins. Upper panel shows the proportion of heritability per bin under the α model from the full set of 503,195 variants (grey bar), the percentage of heritability captured by the 12,968 genotyped variants only (red bars), and the percentage of heritability captured by the genotyped variants and the imputed variants (blue bars). Lower panels show the cumulative proportion of heritability.

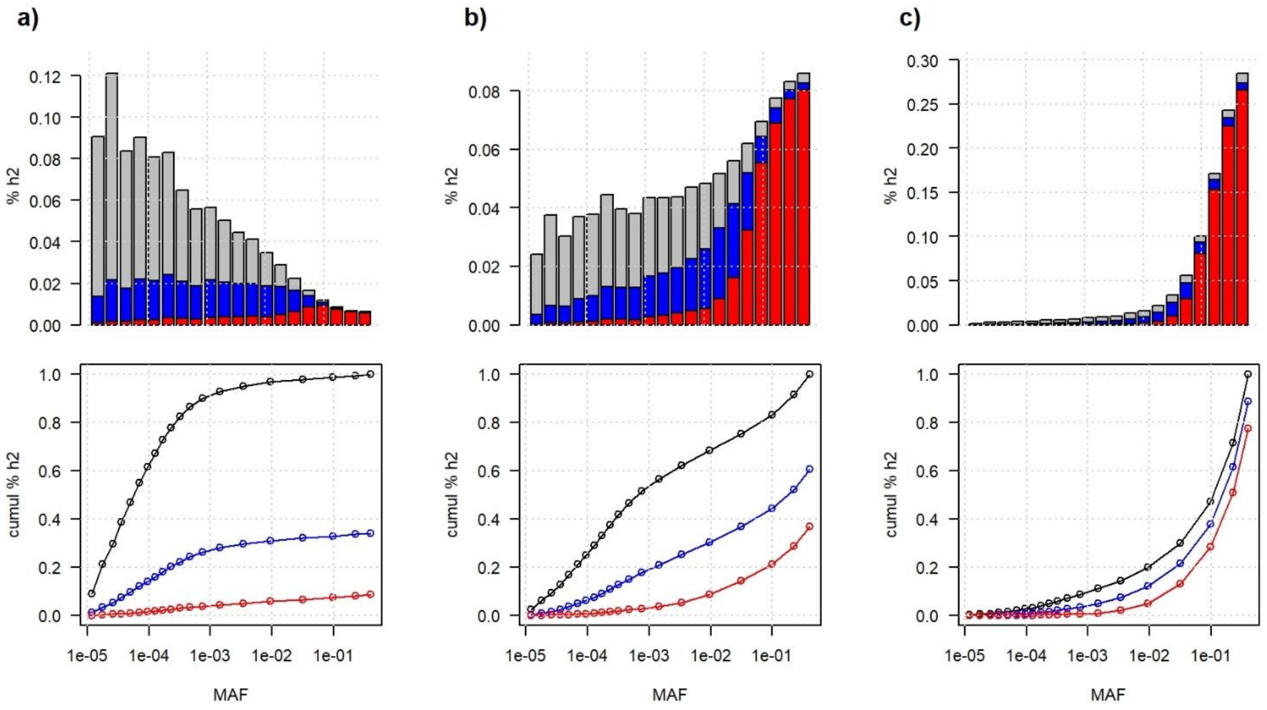

Figure S15. Estimation of alpha using UK Biobank individual-level data

Alpha was estimated using individual-level data from UK Biobank participants for the six outcomes: coronary artery disease (CAD), type 2 diabetes (T2D), breast cancer (BRCA), Alzheimer disease (AZ), asthma (AS), and obesity, but also for body mass index for comparison purposes. We considered three models: the standard alpha model (blue plots), the alpha model after applying an attenuation with a weight factor of $w = 0.6$ (red plots), and $w = 0.95$ (green plots), as described in the method section and Figure S9. The panels present the -log(likelihood) as a function of the alpha use to build the GRM (genetic relationship matrix). Panel a) shows the full data, and panel b) a zoom on the y-abscise to highlight the maximum after applying the R *loss()* smoothing function.

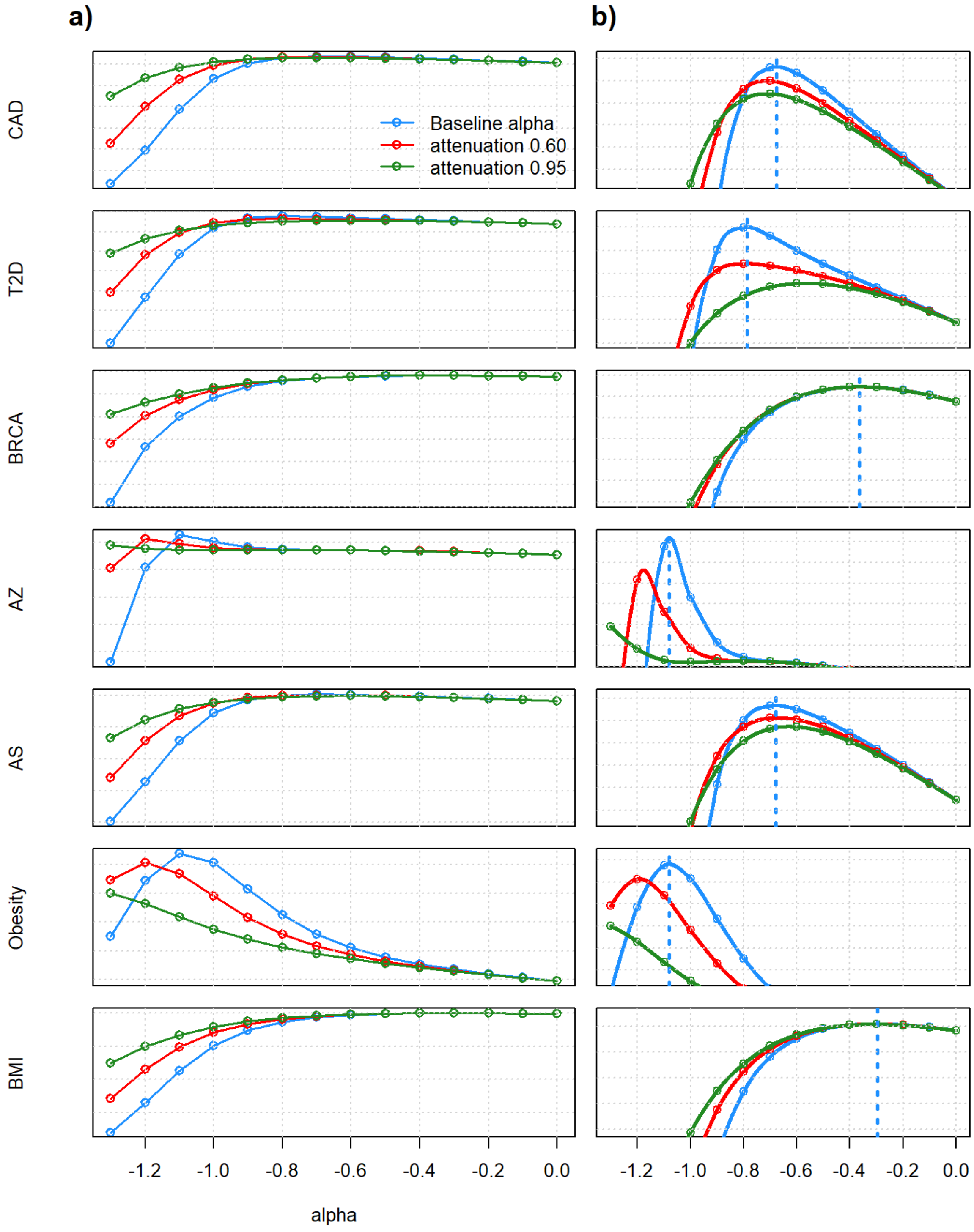

Figure S16. Validation of the GRM-GCTA pipeline using simulated data

Estimate of alpha in simulated data using restricted maximum likelihood method as implemented in GCTA. Using 10,000 UK Biobank participants and variants with MAF over 0.1%, we simulated phenotypes with heritability of 0.5, assuming 1% of the variants to be causal, and drawing effect using alpha values in the range [-1,0] with a step of 0.1. For each simulated phenotype, we computed the log-likelihood of 11 candidate alpha values in [-1,0] and retain the alpha displaying the maximum likelihood. Panel a) presents alpha estimates from models where causal variants were drawn from all variants with a MAF > 0.1%. Panel b) presents alpha estimated from models where causal variants were drawn from variants with a MAF in [0.1% and 1%] and using the corresponding genetic relationship matrix.

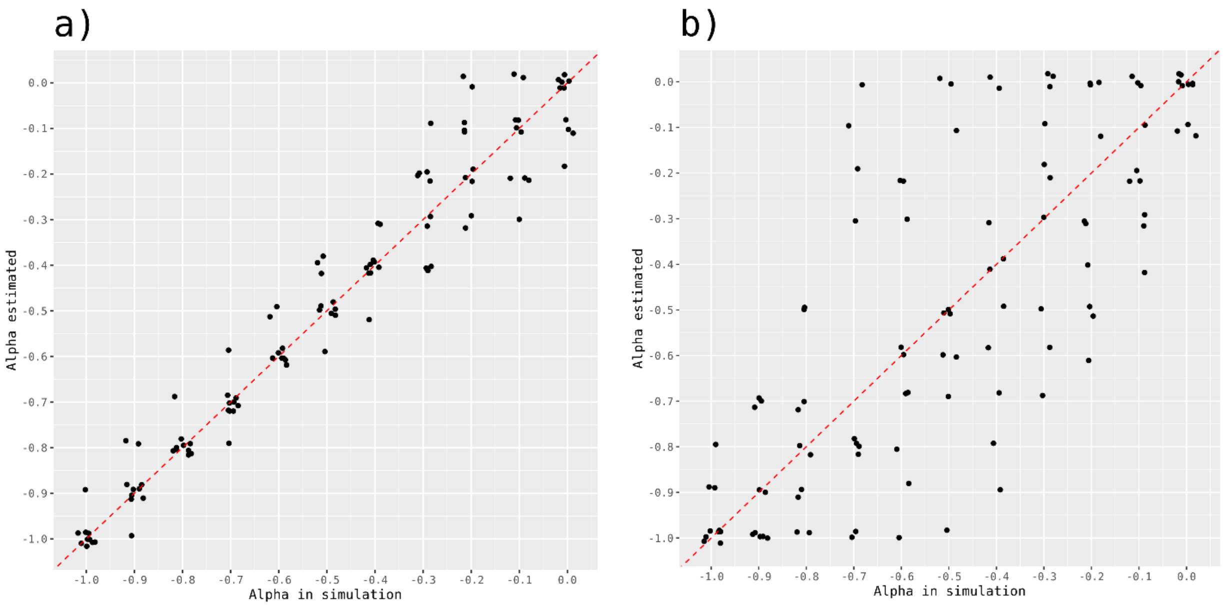

Figure S17. Impact of prevalence on estimated maximum achievable AUC

Estimates of the maximum achievable AUC for six outcomes: coronary artery disease, type 2 diabetes, breast cancer, Alzheimer disease, asthma, and obesity (using body mass index GWAS). Those AUCs were derived based on various estimates of heritability: twin studies (AUC_{Twin}), heritability derived using five competitive approaches: LDSC regression, sumHer, SBayesS, Genesis, and MiXer (AUC_{GWAS}) applied to the largest GWAS available for each disease, and twin study heritability captured by imputed variants ($AUC_{Imputed}$). For each disease, the four columns correspond to four prevalence estimates: US prevalence pulled from the CDC database (used in Figure 3c), and prevalence for US, the Uk and Finland extracted from other sources. Prevalence for asthma, CAD, T2D and Alzheimer disease were extracted from the Institute for Health Metrics and Evaluation (IHME) website (https://vizhub.healthdata.org/gbd-results/?params=gbd-api-2021-permalink/da654804ed46ce98fa58705f8713e236). Prevalence for breast cancer were extracted from Zheng et al 2023[27]. Prevalence for obesity were extracted from the World Health Organisation (WHO) website (https://apps.who.int/gho/data/node.main.A900A). The black dashed lines correspond to the most recent estimates of AUC in real data from the literature, derived based on approximately 1M hapmap3 variants.

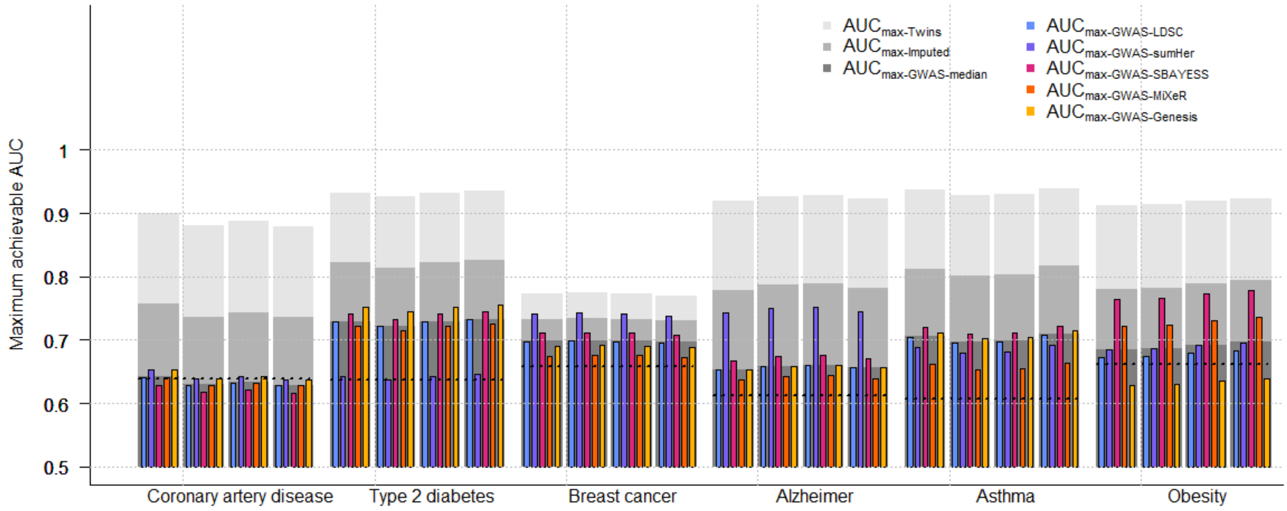

Figure S18. Correlation between functional annotations

Correlation between 1,099 functional annotations from nine categories: GENCODE, TFBS (transcription factor binding site), FANTOM5 (functional annotation of the mammalian genome version 5), promoters, enhancers, and dyadic from Roadmap, DHS (DNase I hypersensitive sites) derived from two studies[28], and super enhancer.

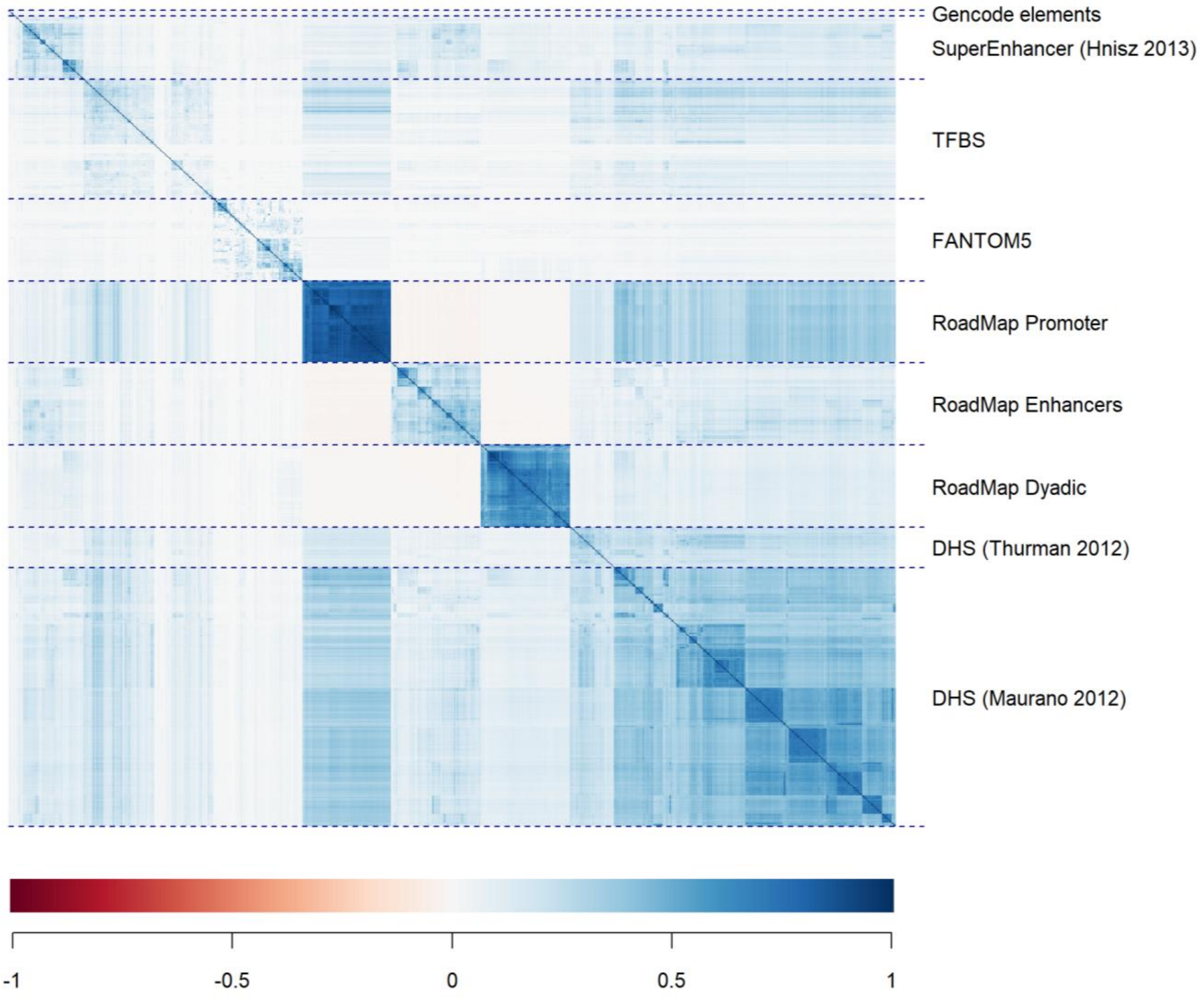

Figure S19. Distribution of imputation quality across GENCODE annotations

Panel a) shows the averaged quality of imputation measured as the squared-correlation (r^2_{imput}) derived between true and imputed genotypes in the UK Biobank for each of the seven GENCODE annotations: intron, gene, exon, CDS (coding DNA sequence), tss (transcription start site), tts (transcription termination site), and UTR (untranslated regions). Panel b) shows the proportion of heritability captured by imputed variants based on r^2_{imput} and assuming genetic effects are distributed following an alpha model. The relationship was derived assuming the causal variants are randomly distributed (red curve), or assuming the causal variants fall only into each of the seven annotations (green to black gradient).

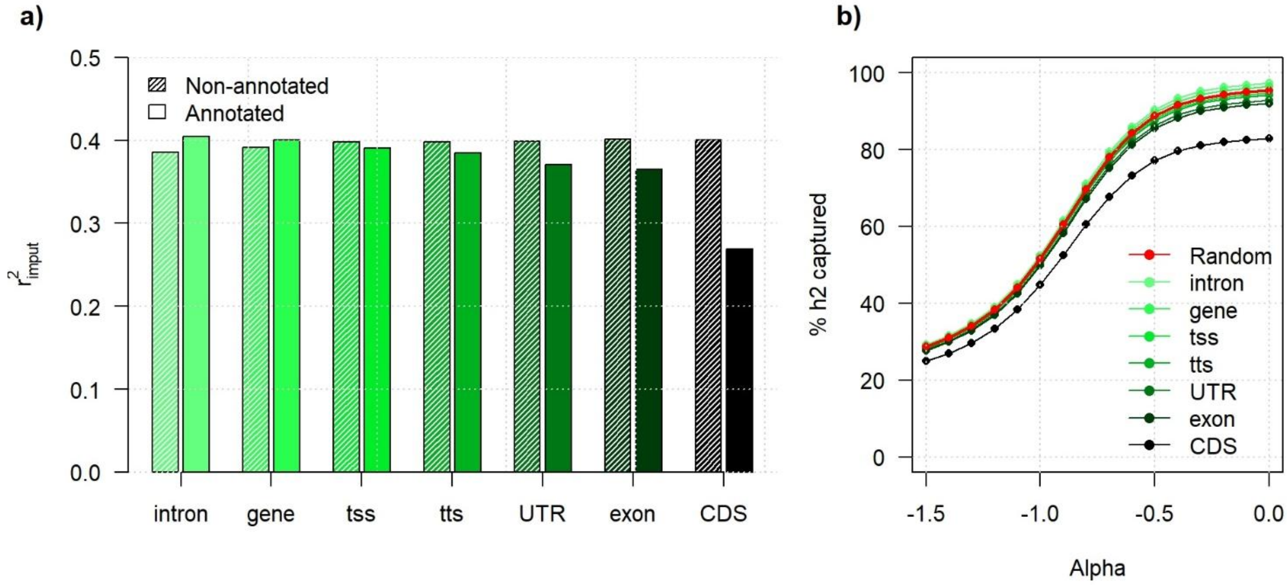

Figure S20. Top annotations associated with imputation quality

Relationship between top functional annotations and the quality of imputation measured as the squared-correlation (r^2_{imput}) between true and imputed genotypes. The barplot displays the change in the average r^2_{imput} between annotated and non-annotated variants for annotation reaching a significance p-value of 1 x10^{-8}. The category of each annotation is indicated by a color code: TFBS (transcription factor binding site), FANTOM5 (functional annotation of the mammalian genome version 5), promoters, enhancers, and dyadic from Roadmap, DHS (DNase I hypersensitive sites) derived from two studies, and super enhancer.

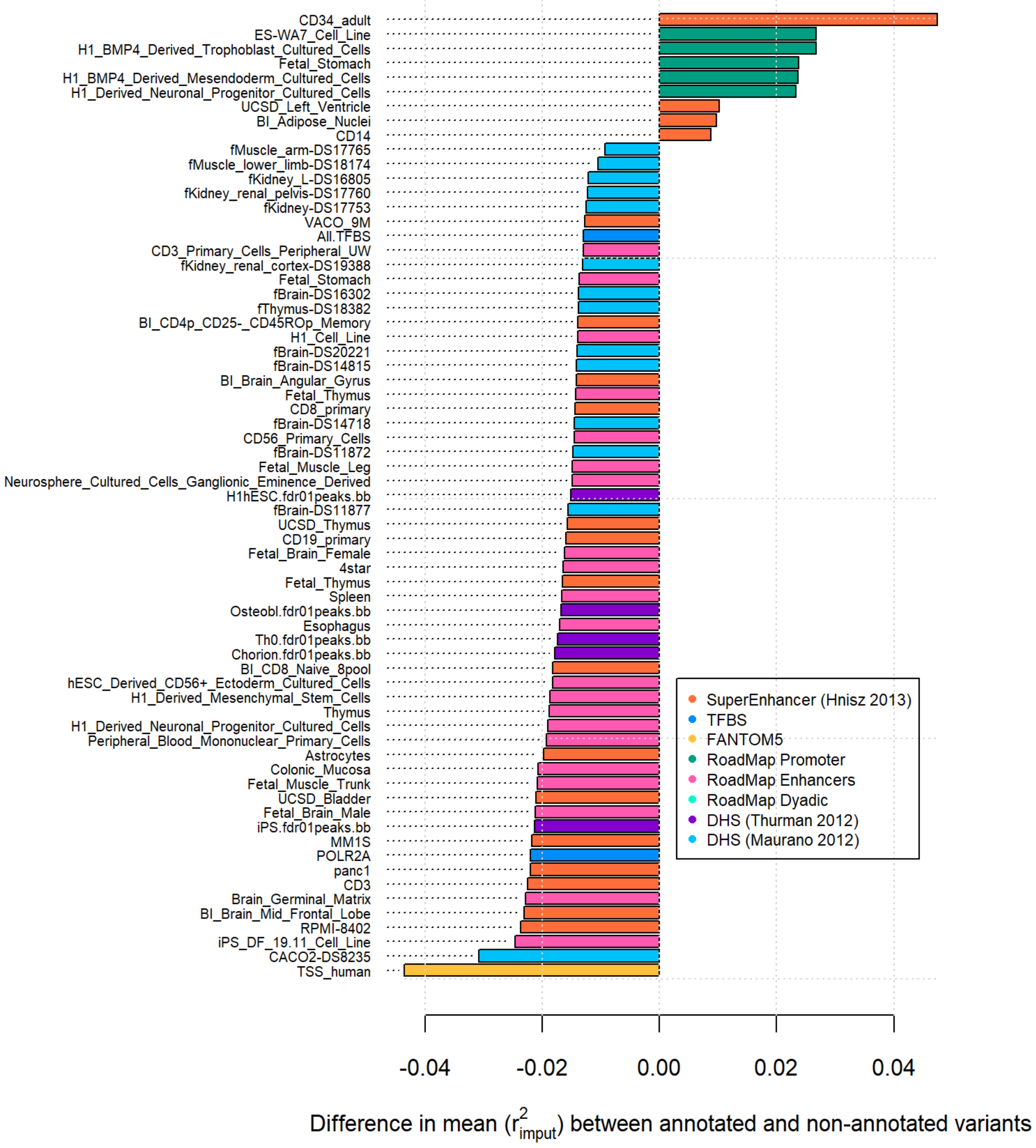

References

1. Prive, F., Luu, K., Blum, M.G.B., McGrath, J.J. & Vilhjalmsson, B.J. Efficient toolkit implementing best practices for principal component analysis of population genetic data. *Bioinformatics* **36**, 4449-4457 (2020).
2. Jansen, I.E. *et al.* Genome-wide meta-analysis identifies new loci and functional pathways influencing Alzheimer's disease risk. *Nat Genet* **51**, 404-413 (2019).
3. Lamnidis, T.C. *et al.* Ancient Fennoscandian genomes reveal origin and spread of Siberian ancestry in Europe. *Nat Commun* **9**, 5018 (2018).
4. Prive, F. *et al.* Portability of 245 polygenic scores when derived from the UK Biobank and applied to 9 ancestry groups from the same cohort. *Am J Hum Genet* **109**, 373 (2022).
5. Han, Y. *et al.* Genome-wide analysis highlights contribution of immune system pathways to the genetic architecture of asthma. *Nat Commun* **11**, 1776 (2020).
6. Mahajan, A. *et al.* Fine-mapping type 2 diabetes loci to single-variant resolution using high-density imputation and islet-specific epigenome maps. *Nat Genet* **50**, 1505-1513 (2018).
7. Zhang, H. *et al.* Genome-wide association study identifies 32 novel breast cancer susceptibility loci from overall and subtype-specific analyses. *Nat Genet* **52**, 572-581 (2020).
8. Yengo, L. *et al.* Meta-analysis of genome-wide association studies for height and body mass index in approximately 700000 individuals of European ancestry. *Hum Mol Genet* **27**, 3641-3649 (2018).
9. Genomes Project, C. *et al.* A global reference for human genetic variation. *Nature* **526**, 68-74 (2015).
10. Lee, S.H., Wray, N.R., Goddard, M.E. & Visscher, P.M. Estimating missing heritability for disease from genome-wide association studies. *Am J Hum Genet* **88**, 294-305 (2011).
11. Liu, C.C., Liu, C.C., Kanekiyo, T., Xu, H. & Bu, G. Apolipoprotein E and Alzheimer disease: risk, mechanisms and therapy. *Nat Rev Neurol* **9**, 106-18 (2013).
12. Prive, F., Arbel, J. & Vilhjalmsson, B.J. LDpred2: better, faster, stronger. *Bioinformatics* (2020).
13. Kunkle, B.W. *et al.* Genetic meta-analysis of diagnosed Alzheimer's disease identifies new risk loci and implicates Abeta, tau, immunity and lipid processing. *Nat Genet* **51**, 414-430 (2019).
14. Karlsson, I.K. *et al.* Measuring heritable contributions to Alzheimer's disease: polygenic risk score analysis with twins. *Brain Commun* **4**, fcab308 (2022).
15. Zhang, Y., Qi, G., Park, J.H. & Chatterjee, N. Estimation of complex effect-size distributions using summary-level statistics from genome-wide association studies across 32 complex traits. *Nat Genet* **50**, 1318-1326 (2018).
16. Zeng, J. *et al.* Widespread signatures of natural selection across human complex traits and functional genomic categories. *Nat Commun* **12**, 1164 (2021).
17. Speed, D. & Balding, D.J. SumHer better estimates the SNP heritability of complex traits from summary statistics. *Nat Genet* **51**, 277-284 (2019).
18. Bulik-Sullivan, B.K. *et al.* LD Score regression distinguishes confounding from polygenicity in genome-wide association studies. *Nat Genet* **47**, 291-5 (2015).
19. Holland, D. *et al.* Beyond SNP heritability: Polygenicity and discoverability of phenotypes estimated with a univariate Gaussian mixture model. *PLoS Genet* **16**, e1008612 (2020).
20. Frei, O. *et al.* Bivariate causal mixture model quantifies polygenic overlap between complex traits beyond genetic correlation. *Nat Commun* **10**, 2417 (2019).
21. International HapMap, C. *et al.* Integrating common and rare genetic variation in diverse human populations. *Nature* **467**, 52-8 (2010).
22. Devlin, B. & Roeder, K. Genomic control for association studies. *Biometrics* **55**, 997-1004 (1999).
23. Speed, D., Hemani, G., Johnson, M.R. & Balding, D.J. Improved heritability estimation from genome-wide SNPs. *Am J Hum Genet* **91**, 1011-21 (2012).
24. Schoech, A.P. *et al.* Quantification of frequency-dependent genetic architectures in 25 UK Biobank traits reveals action of negative selection. *Nat Commun* **10**, 790 (2019).
25. Yang, J., Lee, S.H., Goddard, M.E. & Visscher, P.M. GCTA: a tool for genome-wide complex trait analysis. *Am J Hum Genet* **88**, 76-82 (2011).
26. Chang, C.C. *et al.* Second-generation PLINK: rising to the challenge of larger and richer datasets. *Gigascience* **4**, 7 (2015).

27. Zheng, R. *et al.* Global, regional, and national lifetime probabilities of developing cancer in 2020. *Sci Bull (Beijing)* **68**, 2620-2628 (2023).
28. Thurman, R.E. *et al.* The accessible chromatin landscape of the human genome. *Nature* **489**, 75-82 (2012).
